# Peptides from Animal Origin: A Systematic Review on Biological Sources and Effects on Skin Wounds

**DOI:** 10.1155/2020/4352761

**Published:** 2020-10-23

**Authors:** Raul Santos Alves, Levy Bueno Alves, Luciana Schulthais Altoé, Mariáurea Matias Sarandy, Mariella Bontempo Freitas, Nelson José Freitas Silveira, Rômulo Dias Novaes, Reggiani Vilela Gonçalves

**Affiliations:** ^1^Department of General Biology, Federal University of Viçosa, Viçosa, 36570-900 Minas Gerais, Brazil; ^2^Laboratory of Molecular Modeling and Computer Simulation-MolMod-CS, Institute of Chemistry, Federal University of Alfenas, Alfenas, 37130-001 Minas Gerais, Brazil; ^3^Department of Animal Biology, Federal University of Viçosa, Viçosa, 36570-900 Minas Gerais, Brazil; ^4^Institute of Biomedical Sciences, Department of Structural Biology, Federal University of Alfenas, Alfenas, 37130-001 Minas Gerais, Brazil

## Abstract

**Background:**

Skin wounds are closely correlated with opportunistic infections and sepsis risk. Due to the need of more efficient healing drugs, animal peptides are emerging as new molecular platforms to accelerate skin wound closure and to prevent and control bacterial infection.

**Aim:**

The aim of this study was to evaluate the preclinical evidence on the impact of animal peptides on skin wound healing. In addition, we carried out a critical analysis of the studies' methodological quality. *Main Methods*. This systematic review was performed according to the PRISMA guidelines, using a structured search on the PubMed-Medline, Scopus, and Web of Science platforms to retrieve studies published until August 25, 2020 at 3 : 00 pm. The studies included were limited to those that used animal models, investigated the effect of animal peptides with no association with other compounds on wound healing, and that were published in English. Bias analysis and methodological quality assessments were examined through the SYRCLE's RoB tool.

**Results:**

Thirty studies were identified using the PRISMA workflow. In general, animal peptides were effective in accelerating skin wound healing, especially by increasing cellular proliferation, neoangiogenesis, colagenogenesis, and reepithelialization. Considering standardized methodological quality indicators, we identified a marked heterogeneity in research protocols and a high risk of bias associated with limited characterization of the experimental designs.

**Conclusion:**

Animal peptides show a remarkable healing potential with biotechnological relevance for regenerative medicine. However, rigorous experimental approaches are still required to clearly delimit the mechanisms underlying the healing effects and the risk-benefit ratio attributed to peptide-based treatments.

## 1. Introduction

Due to the disruption of innate defense mechanisms, skin wounds are a serious risk factor for opportunistic infections, bacteremia, and sepsis [1–3]. In the United States, recent estimates indicate that at least US$25 billion are spent annually in the treatment of 6.5 million patients with chronic wounds [4]. The treatment of skin wounds is a challenging task, especially considering that the available treatments have limited spectrum of action on cellular and molecular mechanisms involved in tissue repair [5–9]. Skin wound healing requires a series of cellular and molecular interdependent events in order to restore tissue integrity after trauma [5]. This process is mediated by growth factors, cytokines, and resident and transitory cells and is organized in phases involving inflammation, cell proliferation, and tissue remodeling/maturation [6]. In the inflammatory phase, immune cells such as neutrophils and macrophages migrate to the lesion area to remove tissue debris, promote antimicrobial defenses, and trigger cell proliferation [7]. The proliferative phase is marked by intense cellular activity and different cell migration to the wound bed. At this stage, fibroblasts form the granulation tissue, composed of cells and a network of blood vessels, reestablishing regional circulation [8]. The remodeling phase corresponds mainly to changes in the extracellular matrix of the scar tissue, where most type III collagen fibers are progressively replaced by type I fibers, which are more resistant and abundant in intact skins [9]. Two subsets of macrophages (M1 or M2) are commonly identified in this process, exerting complementary effects in early and late stages of tissue repair [10]. M1 macrophages are activated by interferon-gamma (IFN-*γ*), exerting potent nitric oxide-mediated antimicrobial effects and proinflammatory responses in the initial stages of tissue repair [11]. As an overlapping event between proliferative and remodeling phases, M2 macrophages are activated by cytokines such as IL-4, IL-10, or IL-13 [11]. These cells play an essential role on the effective resolution of the inflammation, mainly through angiogenesis and extracellular matrix resorption and remodeling [10, 11].

Wound healing is a complex and time-sensitive process often impaired by several factors such as infections, metabolic comorbidities (i.e., diabetes, dyslipidemia, malnutrition, and circulation disorders such as thrombosis, atherosclerosis, and hemorrhage), as well as the presence of foreign bodies that may delay wound healing by stimulating a chronic inflammatory response [1]. In a continuous effort to improve the pharmacological management of skin wounds, the screening of natural molecules capable of modulating the biological processes involved in tissue repair is proposed as a rational and promising strategy for the biotechnological development of more efficient healing drugs [12]. In order to achieve greater therapeutic efficacy, the search for new molecules also is aimed at overcoming current limitations of healing drugs, especially the technical difficulty in obtaining the active metabolites, the high cost of drug production, the formation of hypertrophic scars, and the risk of selecting treatment-resistant microorganisms, an aspect that represents a global concern [13, 14].

Due to its antimicrobial, immunomodulatory, promitotic, colagenogenic, and neoangiogenic potential, animal peptides are suggested as promising agents for new therapeutic approaches in skin wound treatment [1, 12, 15, 16]. Besides, their molecular abundance, low cost of isolation techniques, high molecular stability, and their broad spectrum of biological properties are also encouraging characteristics. However, the main animal peptides, physicochemical characteristics of the bioactive molecules, effective doses, and routes of administration are not completely understood. Considering that current evidence is based on fragmented data, it is unclear whether and to what extent animal peptides are effective in the skin wound treatment. In addition, it is currently difficult to understand the metabolic pathways and mechanisms of actions activated by these peptides during skin repair. Thus, we used the systematic review framework to evaluate preclinical evidence on the impact of animal peptides on skin wound healing. In addition to characterize the biological sources of these peptides and its chemical sequences, the methodological quality of all studies reviewed was critically evaluated.

## 2. Methodology

### 2.1. Retrieval of Research Records

This systematic review followed the Preferred Reporting Items for Systematic Reviews and Meta-Analyses (PRISMA) workflow [17], which is used as a guide for study selection, screening, and eligibility. Studies were selected through an advanced search on the platforms PubMed-Medline (https://www.ncbi.nlm.nih.gov/pubmed), Scopus (https://www.scopus.com/home.uri) and Web of Science (https://www.webofknowledge.com), on August 25, 2020, at 3 : 00 pm. We used a comprehensive search strategy for retrieving all relevant studies, with a primary search in electronic databases and a secondary search in the reference lists from all relevant studies identified in the primary search. For all databases, the search filters were based on three complementary levels: (i) intervention: animal peptides; (ii) biological process: wound healing; and (iii) target organ: skin. The PubMed-Medline platform filters were built using the hierarchical distribution of MeSH (Medical Subject Headings) terms to retrieve the indexed studies. Non-MeSH descriptors were characterized by the TIAB algorithm (Title and Abstract). To identify preclinical studies, a standardized experimental animal filter was applied [18]. The search filters used for the PubMed-Medline search platform were adapted to Scopus and Web of Science databases, except for the experimental animal filter used in Scopus, which was provided by the site. The complete search strategy is shown in the supplementary file (S1 Table).

### 2.2. Selection of Relevant Studies

Only studies that met all the inclusion criteria as described below were selected: (i) *in vivo* studies using animal models; (ii) studies that investigated the effect of animal peptides with no association with other compounds on wound healing; and (iii) original studies published in English. The following studies were excluded: (i) nonanimal peptides; (ii) unreported origin of peptides; (iii) investigations of other organs, pathologies, or therapies; (iv) sutured wounds; (v) *in vitro* and *ex vivo* studies; (vi) unreachable studies; (vii) secondary research (i.e., literature reviews, comments, letters, and editorials); and (viii) gray literature (i.e., video-audio media). When it was difficult to obtain the full-text papers, the authors were requested to provide it by email.

### 2.3. Data Extraction and Management

Two independent reviewers (RSA and LSA) conducted the literature search, removed duplicated articles, and screened titles and abstracts with respect to eligibility criteria. After initial screening, full-text articles of potentially relevant studies were independently assessed for eligibility by two reviewers (RSA and LSA). The kappa test was done for the selection and data extraction (kappa = 0.922). Selections were then compared, and inconsistencies were resolved in consultation with three other reviewers (MMS, RDN, and RVG). Data from each study were extracted using well-defined data as follows: (i) publications characteristics (author, year of publication, and country of origin); (ii) animal models (animal, strain, sex, age, weight, and associated pathology); (iii) cutaneous wounds (type of lesion, site, initial area, number, and presence of infection); (iv) peptide characteristics (name, origin, and amino acid sequence); (v) intervention characteristics (route of administration, concentration, vehicle, frequency, and duration); (vi) primary outcome (wound closure); and (vii) secondary outcomes (cell proliferation and differentiation, synthesis of extracellular matrix components, recruitment of inflammatory cells, neoangiogenesis, inflammatory mediators, and oxidative markers). Quantitative data related to the wound area were directly collected from the tables or the main text provided in each study. When these data were graphically represented, the values of the wound area were obtained using the Image-Pro Plus 4.5 image analysis software (Media Cybernetics, MD, USA). The wound area was compared amongst experimental groups, and the results were expressed in percentage of wound closure.

### 2.4. Bias Analysis

The risk of bias was analyzed using the SYstematic Review Centre for Laboratory animal Experimentation (SYRCLE) Risk of Bias (RoB) tool [19]. This instrument is based on the Cochrane Collaboration RoB Tool, which is adjusted for aspects of bias that play a specific role in animal intervention studies. The goal was to avoid discrepancies in the assessment of methodological quality in the field of animal experimentation. To increase transparency and applicability, signaling questions were answered to facilitate judgment based on the following domains: (i) sequence generation; (ii) baseline characteristics; (iii) allocation concealment; (iv) random housing; (v) blinding; (vi) random outcome assessment; (vii) incomplete outcome data; (viii) selective outcome; and (ix) other sources of bias. Two reviewers (RSA and RVG) independently assessed the risk of bias for each study; any disagreements were resolved by discussion and consensus with two other reviewers among the authors (MMS and RDN). The SYRCLE chart was built using the Review Manager 5.3 program (Copenhagen: The Nordic Cochrane Centre, The Cochrane Collaboration).

## 3. Results

### 3.1. Included Studies

**Figure 1:**
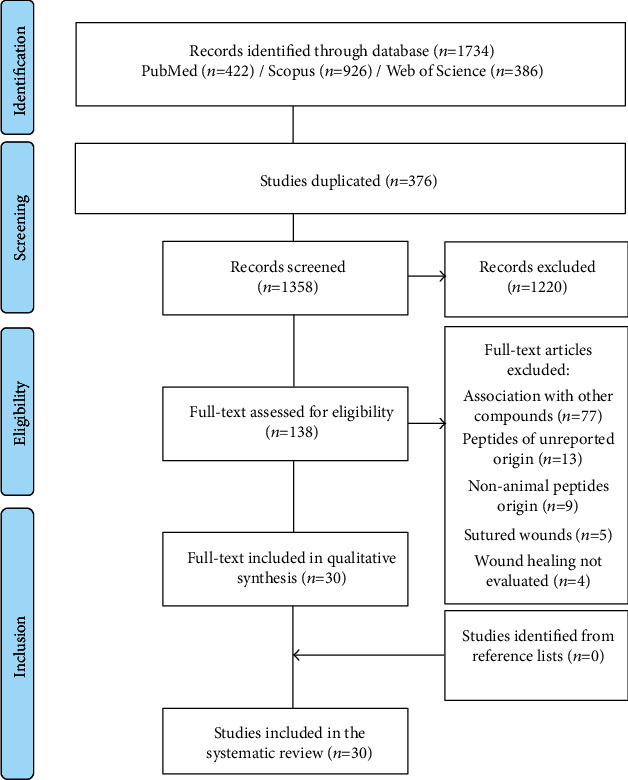
PRISMA (Preferred Reporting Items for Systematic Reviews and Meta-Analyses) flow diagram. The flowchart indicates the research records obtained at all standardized stages of the search process required for the development of systematic reviews and meta-analyses. Based on the PRISMA statement (http://www.prisma-statement.org).

We found 1734 articles, of which 376 were duplicated and 1220 studies were excluded due to inadequate research theme. Among the excluded studies, 502 did not use peptides; 458 were related to other tissues, pathologies, or therapies; 108 were reviews; 68 did not evaluate the wound healing process; 57 used peptides of nonanimal origin; 10 were unreachable; 9 were not written in English;3 were studies *in vitro*; 2 were comments; 1 was an *ex vivo* study; 1 was a letter; 1 was a video-audio media. The remaining 138 articles were carefully analyzed, of which 108 were excluded for not meeting the eligibility criteria (S2 Table). Thus, 30 relevant articles were selected. After reading the reference list of all selected articles, no relevant article was found. PRISMA diagram indicates the study selection process (Figure 1). The selected studies were conducted in 7 different countries, mainly China (*n* = 18, 60%), followed by Taiwan and United States of America (*n* = 4, 13% each), Portugal, Korea, India, and Saudi Arabia (*n* = 1, 3% each).

### 3.2. Characteristics of Preclinical Models

The most used animal model was mice (*n* = 20, 67%), followed by rat (*n* = 8, 27%), pig, and rabbit (*n* = 1, 3% each). The most used strain was Balb/C for mice (*n* = 6, 30%) and Sprague-Dawley for rat (*n* = 6, 75%), but 30% of the studies did not report this information (*n* = 9). Most studies included only males (*n* = 19, 63%), 17% used only females (*n* = 5), 3% used both (*n* = 1), and 17% did not report this data (*n* = 5). The age of the animals ranged from 6 to 12 weeks for mice, 6 to 43 weeks for rats, and 6 weeks for pigs. This information was not reported in 57% of the studies (*n* = 17). Animal weight ranged from 20 to 26 g for mice, 150 to 600 g for rats, and 10 to 13 kg for pigs. This information was underreported in most studies (*n* = 14, 47%). Most studies were performed on health animals (*n* = 26, 87%), 10% used diabetic models (*n* = 3), and 3% used ischemic model (*n* = 1). The main characteristics related to animal models are described in detail in S3 Table.

### 3.3. Characteristics of Skin Wounds

Most studies investigated excisional wounds (*n* = 27, 90%), followed by burns (*n* = 2, 7%) and incisional wounds (*n* = 1, 3%). The most used site for wounds was the back of the animal (*n* = 29, 97%) and 3% performed the injury on the abdomen (*n* = 1). The initial wound area was reported in all studies (*n* = 30, 100%). The number of wounds ranged from 1 to 6 per animal (*n* = 27, 90%), and 10% did not report this information (*n* = 3). *Staphylococcus aureus* and *Escherichia coli* were the microorganisms used in experiments with infected wounds (*n* = 5, 17%), and bacterial load concentration ranged from 2 × 10^5^ to 10^10^ Colony Forming Unit (CFU). The main characteristics related to skin wounds are detailed in S4 Table.

### 3.4. Characteristics of Animal Peptides and Treatments

The name and origin of the peptides used were reported in all studies (*n* = 30, 100%). Most of the peptides originated from amphibians (*n* = 11, 37%), followed by mammals and fishes (*n* = 8, 27% each), jellyfish, mollusk, and insect (*n* = 1, 3% each). The amino acid sequences in these peptides were described in 63% of the studies (*n* = 19). The most commonly used route of peptide administration was topical (*n* = 20, 67%), followed by oral (*n* = 6, 20%), subcutaneous (*n* = 2, 7%), intravenously (*n* = 1, 3%), and 3% evaluated two routs (topic and intraperitoneal) (*n* = 1). The most used vehicle was saline solution (*n* = 13, 43%), followed by phosphate-buffered saline (*n* = 10, 33%), Dulbecco's phosphate-buffered (*n* = 2, 7%), water (*n* = 2, 7%), and 10% did not report this information (*n* = 3). Most studies applied the intervention twice a day (*n* = 10, 33%), followed by once a day (*n* = 8, 27%), single application (*n* = 2, 7%), three times per day, continuous intervention, every three days, and twice or every two days (*n* = 1, 3% each). In 20% of the studies, this information was underreported (*n* = 6). Duration of intervention ranged from 5 to 11 days in 27% of studies (*n* = 8), 12 to 16 days in 10% of studies (*n* = 3), 22 to 26 days in 3% of the studies (*n* = 1), 27 to 31 days in 3% of the studies (*n* = 1), and 57% did not report this data (*n* = 17). The peptide-related characteristics and treatment protocols are described in S5-S6 Tables, respectively. Main outcome (reduction in wound size) in the treatment of skin wounds using peptides of animal origin is described in Table 1.

### 3.5. Main Biological Outcomes

In general, studies identified in this review support the evidence that animal peptides exert healing properties on skin wound. Although the reports are heterogeneous, all studies (*n* = 30, 100%) show that animal peptides are effective in accelerating wound closure. Most studies that performed histological analysis (*n* = 23; 77%) reported improvement in the processes of reepithelialization and dermal regeneration, inflammatory cell recruitment, and blood vessel and collagen fiber formation. Immunohistochemical analyses were performed in 50% of the studies (*n* = 15), which showed the effects of peptides in the quantitative increase of myofibroblasts, inflammatory cells, blood vessel density, and growth factors such as factors *β*-fibroblast growth factor (*β*-FGF), vascular endothelial growth factor (VEGF), and transforming growth factor-*β*1 (TGF-*β*1), as well as the reduction of proinflammatory cytokines such as interleukin-1*β* (IL-1*β*), interleukin-6 (IL-6), and tumor necrosis factor-*α* (TNF-*α*). Enzyme-linked immunosorbent assay (ELISA) was performed in 33% of the studies (*n* = 10), which reported a reduction in proinflammatory cytokines such as IL-6 and TNF-*α*, as well as an increase in growth factor VEGF and TGF-*β*1. Reverse transcription-polymerase chain reaction (RT-PCR) was performed in 10% of the studies (*n* = 3), which highlighted the influence of peptides on the upregulation of growth factor-related genes such as epidermal growth factor (EGF), transforming growth factor-*β* (TGF-*β*), and VEGF, and also on the gene related to macrophage migration inhibition factor (MIF), and downregulation of genes related to proinflammatory cytokines such as IL-6 and TNF-*α*, as well as the expression of the CXCL5 gene. The Western blot technique was performed in 7% of the studies (*n* = 2), which highlighted the increased expression of angiogenic proteins such as hypoxia-inducible factor-1*α*, endothelial nitric oxide synthase, and inducible nitric oxide synthase, as well as VEGF and TGF-*β*1. Oxidative stress analysis was performed in 7% of the studies (*n* = 2), in which peptides increased glutathione (GSH) level and the activity of antioxidant enzymes such as superoxide dismutase (SOD) and catalase (CAT); as well as reduced the level of malondialdehyde (MDA), a lipid peroxidation marker. All relevant results involving the use of animal peptides in the treatment of skin wounds are described in Table 2.

### 3.6. Reporting Bias

**Figure 2:**
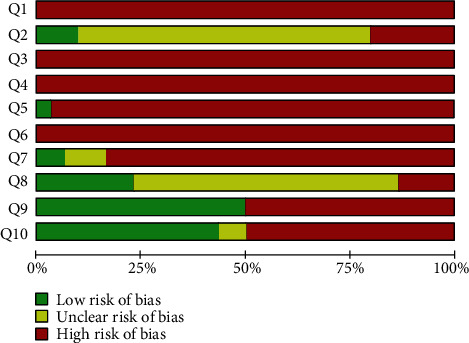
Results of the risk of bias for all studies included in the systematic review. The items in the SYstematic Review Centre for Laboratory animal Experimentation (SYRCLE) Risk of Bias assessment (Q1–Q10) were scored with “yes” indicating low risk of bias, “no” indicating high risk of bias, or “unclear” indicating an unclear risk of bias. Q1–Q3 consider selection bias, Q4–Q5 consider performance bias, Q6–Q7 consider detection bias, Q8 considers attrition bias, Q9 considers reporting bias, and Q10 considers other biases. Q: Question. Q1: Was the allocation sequence adequately generated and applied?; Q2: Were the groups similar at baseline or were they adjusted for confounders in the analysis?; Q3: Was the allocation adequately concealed?; Q4: Were the animals randomly housed during the experiment?; Q5: Were the caregivers and/or investigators blinded from knowledge which intervention each animal received during the experiment?; Q6: Were animals selected at random for outcome assessment?; Q7: Was the outcome assessor blinded?; Q8: Were incomplete outcome data adequately addressed?; Q9: Are reports of the study free of selective outcome reporting?; and Q10: Was the study apparently free of other problems that could result in high risk of bias?

Regarding the analysis of bias obtained with SYRCLE's RoB tool, the highest risks of bias found in the studies were related to the methods used in the generation and application of the animal allocation sequence, housing procedures, and animal selection for outcome assessment. Regarding baseline similarities, 10% of the studies reported sufficient information to conclude that the distribution was balanced among the intervention and control groups at the beginning of the experiment (*n* = 3), and 90% did not report sufficient information on the homogeneity of the experimental models (*n* = 27). Regarding the measures used to blind caregivers and/or investigators, only 3% reported this information (*n* = 1). Considering the evaluators, two studies (7%) reported that the outcomes were collected in a blind manner, 10% reported that the evaluation was performed by independent researchers, but does not provide information on blinding (*n* = 3), and 83% did not report this information at all (*n* = 25). Regarding incomplete results adequately addressed, 77% did not report or showed unclear information (*n* = 23). Considering the item that evaluates whether the study is free of selective outcome reports, 50% did not make clear the expected results (*n* = 15). Other potential risks of bias that could compromise the evidence (i.e., additional treatment or drugs and interventions applied to different parts of the body within one participant) were found in 50% of the studies (*n* = 15). Results from bias analysis are shown in Figure 2.

## 4. Discussion

In order to meet a comprehensive interpretation of the evidence reported in this systematic review, in addition to the research outcomes, we conducted an analysis of the experimental models used in the selected studies to investigate the impact of animal peptides on skin wound healing. In our view, mapping these peptides and selecting well-designed animal models are critical for assessing the effectiveness of new molecules with healing potential. These aspects can contribute to clarify the potential biotechnological applicability of peptide-based strategies in regenerative medicine, an essential assumption to support clinical trials [45].

### 4.1. Relevance of Animal Models in Studies on Skin Wound Healing

Although pigs were used in only one study identified in the systematic review, this is the animal model whose skin is more similar to humans, which makes them an interesting model for preclinical studies on wound healing [46]. However, as these animals demand high husbandry costs and more restrictive ethical issues, their use has been increasingly limited. In contrast, mice and rats were the most used animal models, an aspect potentially associated with its greater availability, low cost, and easy handling. In addition, mice, rats, and humans exhibit the same stages of wound healing, with immunoinflammatory and microstructural convergences mainly based on similar profiles of regulatory molecules (i.e., cytokines and growth factors) and composition of extracellular matrix (i.e., glycosaminoglycan's, collagen and noncollagen proteins) [47].

Rodents, especially mice and rats, are also often useful to investigate the effect of healing agents in pathological conditions such as diabetes [28, 31, 32], which was the associated disease most investigated in the studies reviewed. While streptozotocin was used to induce type I diabetes [31, 32], type II diabetes was studied using *db/db* mice model [28]. Although diabetes develops from different physiopathological mechanisms in streptozotocin-induced and *db/db* animals, both models are valid to investigate the human disease. In this sense, induced-animals and diabetic humans share similar metabolic abnormalities, especially hyperglycemia, vasculopathy, and neuropathy [48]. As these are disturbances associated with delayed wound healing in diabetes [49], chemically-induced and genetic models represent robust and realistic experimental constructs, which exhibits marked relevance and applicability in studies on healing products [28, 31, 32].

### 4.2. Relevance of Wound Models

The frequent use of rodents, excision wounds were consistently investigated in the studies reviewed. However, the number and size of the wounds were highly variable. Due to the complete skin removal, all phases of tissue repair are more pronounced in excisional than in incisional wounds [50]. Thus, excisional injuries are widely used in second intention healing models [22, 26, 31, 40]. In these cases, the intense inflammatory process and the marked tissue remodeling favor the analysis of the effectiveness of healing products [26]. In addition to the type (first vs. second intention), the number of wounds exerts a relevant impact on the therapeutic outcome. Although most studies evaluated the healing potential of animal peptides on 1 or 2 wounds produced in each animal, 4 and 6 wounds were also reported. The main limitations of models based on multiple wounds are related to repeated biopsies on nearby wounds [22, 51]. As wound tissue collection creates additional damage to the skin, the acute inflammatory process is reactivated [51]. In this case, the upregulation of cytokine and growth factors might influence the adjacent wound repair [52]. Thus, it would be ideal to investigate changes in only 1 wound per animal, to reduce the construct bias and its impact on the evidence. However, as models with 2 or more wounds are often required in time-dependent analysis of the healing process, the selection of these models should be carefully considered.

Regarding investigations on infected wounds, *S. aureus* was consistently used to induce wound infection. As *S. aureus* is an important human pathogen often associated to bacterial skin infections [53], preclinical models based on this bacteria are relevant and realistic. The emergence of multidrug-resistant microorganisms stimulates an important challenge in regenerative medicine: the development of more efficient products to treat infected wounds [14]. Efficient antimicrobial products are also relevant since the colonization of wounds by microorganisms amplifies inflammation and oxidative tissue damage, slowing or inhibiting the progression of the healing process [12, 26, 34]. Thus, studies on the treatment of infected wounds are urgent, especially considering that controlling infection is essential to reduce the risk of developing chronic wounds [54].

### 4.3. Relevance of Therapeutic Protocols

Although most studies used a diluted aqueous solution and applied the peptides topically, the number of applications and the treatment period was highly variable. The use of water, saline, or sodium phosphate buffer as a vehicle indicated that animal peptides exhibit an interesting hydrophilic characteristic. These vehicles are relevant to avoid the development of cytotoxicity, which can occur with the use of organic solvents such as ethanol and dimethyl sulfoxide [55]. Unlike recommendations for different types of vehicles, there is no consensus on the dose and duration of treatment. Essentially, these aspects of dosimetry depend on the biological effect and the organic tolerability of each molecule. Thus, although the therapeutic effects are influenced by the dose and time of treatment, generalizations cannot be established for molecules with potentially different chemical and biological properties.

### 4.4. Effect of Animal Peptides on Wound Healing

Currently, identifying animal peptides with healing properties opens a new perspective for the treatment of skin wounds [16, 32, 34]. In general, reviewed studies indicate that peptides originating from mammals, amphibians, fishes, jellyfish, mollusk, and insect exert beneficial effects in stimulating wound closure. However, peptides obtained from the fish species *Pardachirus marmoratus* [26] and *Oreochromis niloticus* [12, 30] demonstrated positive effects only in infected wounds, suggesting that some peptides facilitate wound recovery by exerting antimicrobial effects and controlling opportunistic infections. This feature might be associated with the peptides' biochemical characteristics, since the peptides tilapia piscidin 3 (TP3) and tilapia piscidin 4 (TP4), both originating from *Oreochromis niloticus*, have similar amino acids sequence and share the same antibacterial action. Studies that evaluated mainly the antibacterial characteristics of the animal peptides showed a reduction of bacterial load on the wound area after treatment [12, 26].

Animal peptides have been shown to act on the activation and proliferation of different cells involved in the wound healing process. The increase in fibroblasts, myofibroblasts, and endothelial cells potentiate the processes of dermal regeneration and wound closure, acting on the formation, contraction, and nutrition of granulation tissue, respectively [25, 27, 36, 41]. Several peptides were found to increase blood vessel density, in order to adequate nutrient and oxygen delivery to newly formed tissue [20, 21, 24, 33]. There is evidence that animal peptides may increase VEGF biosynthesis and stimulate neoangiogenesis, which is essential for a more efficient healing due to the influx of molecules required for the proper morphofunctional organization of the scar tissue [26, 30, 33, 36]. TGF-*β*1 was additionally increased in response to animal peptides, which is a growth factor effective in stimulating cell proliferation, differentiation, and migration; as well as colagenogenesis in the granulation tissue [13, 27, 36, 39]. In addition, peptides obtained from mammals, amphibians, fishes, and insect increased the recruitment of inflammatory cells, which contributes to the removal of damaged cells and matrix debris in the injured tissue and protects against local infections during the inflammatory phase, accelerating wound closure [13, 21, 23, 26, 27, 34]. Several cells of the immune system are involved in wound healing, such as neutrophils, monocytes/macrophages, mast cells, and lymphocytes [21]. Most studies have evaluated the effect of peptides on the recruitment of macrophages due to their critical roles in the healing process, coordinating complex processes of cell proliferation and extracellular matrix biosynthesis [12, 13, 21, 26, 27]. However, the peptide TP3, originating from the fish *Oreochromis niloticus*, was effective in reducing the number of inflammatory cells in infected wounds, an effect related to the peptide's antimicrobial action in directly attenuating tissue bacterial load, reducing the antigenic load and immunological activation [12]. Although it remains poorly understood, these results help to clarify potential mechanisms of action of the peptides and their modulating action in skin wound healing.

Some studies included in this review have evaluated the skin healing effect of animal peptides when comorbidities also occur, such as ischemia and diabetes. In the ischemic animal model, TP508 peptide from human thrombin significantly accelerated wound closure by stimulating anti-inflammatory processes and increasing tissue vascularization [22]. Diabetes was a condition widely studied, since the skin wound healing is often interrupted or delayed by abnormal glycation products and microvascular disturbances, contributing to the development of chronic wounds [56–59]. In addition, diabetic wounds often remain in the inflammatory stage for a long time, impairing the healing process due to the release of proinflammatory cytokines such as IL-6 and TNF-*α* [60]. Thus, studies evaluating the relevance of animal peptides on skin wound healing in diabetic animals are required, especially considering essential parameters such as immunological effectors, neoangiogenesis, and wound closure to characterize the effect of the treatments. In diabetic models, most peptides identified significantly stimulated wound closure compared to untreated animals, increasing vascularization [31, 32], colagenogenesis, and dermal regeneration [32]. In addition, the human proinsulin C peptide reversed the increase of inflammatory cells in diabetic wounds, preventing an excessive inflammatory response and extensive secondary tissue damage, and consequently stimulating the rapid progression from the inflammatory to the proliferative phase [31]. These effects were associated with decreased proinflammatory cytokine production. Camel milk peptide increased the activity of antioxidant enzymes such as SOD, CAT, and GST, reducing the negative effects of excessive reactive oxygen species formation and lipid peroxidation [32]. However, a study testing CW49 peptide originating from the amphibian *Odorrana grahami* indicated a moderate effect on the healing process in a diabetic model [28]. In this study, wound closure was improved only in the early stages in the healing progression, when increased reepithelialization and dermal regeneration rates, blood vessel density, proangiogenic proteins, and reduced recruitment of inflammatory cells and proinflammatory cytokines were reported.

### 4.5. Limitations

Systematic reviews are essential tools for summarizing evidence accurately and reliably, assisting risk assessment, and providing evidence of the benefits of health-related interventions [61]. However, the methodological quality of the studies included in this review was predominantly classified as high risk or unclear risk of bias, indicating that most features needed for a bias study evaluation were not sufficiently reported. Incomplete characterization of animal models, peptide acquisition and characterization, treatment protocols, outcome measures, and mechanisms involved in the healing process all contributed to the increased risk of bias. Along with these limitations, results were presented only as graphics in most studies, which made it difficult to assess the absolute values related to the wound area. We hope that our critical analysis helps accelerating preclinical research and reducing methodological bias by improving experimental control and accuracy of research reports.

## 5. Conclusion

In general, we identified that the evidence on the healing potential of animal peptides is mainly based on valid and realistic preclinical models that share similar tissue repair phases with those observed in humans. From studies using these models, we identified that animal peptides are potentially effective in accelerating the skin wound healing. For most of the identified peptides, the beneficial effect is mainly associated with cell proliferation stimulation, neoangiogenesis, colagenogenesis, reepithelization, and wound contraction. However, the healing property of a small group of tilapia-derived peptides (TP3 and TP4) is potentially related to the antibacterial effects of these molecules. Despite the beneficial healing effects, the risk of bias and methodological divergences observed in some studies make the current evidence limited to the experimental contexts applied to the animal models analyzed. Considering that research papers on animal peptides promoting wound healing are relatively recent, there is a growing need to increase the number of investigations and improve the experimental protocols and research reports. We hope that our critical analysis helps accelerating preclinical research and reducing methodological bias by improving experimental control and accuracy of the research reports in this area.

## Figures and Tables

**Table 1:** Main outcome in the treatment of skin wounds using peptides of animal origin.

		Intervention			Main outcome∗									
					Normal wound		Infected wound		Diabetic wound		Ischemic wound		Radiation + wound	
RF	P	R	A	C	RWS (%)	DA (PI)	RWS (%)	DA (PI)	RWS (%)	DA (PI)	RWS (%)	DA (PI)	RWS (%)	DA (PI)
[20]	Thymosin *β*4	Topic	Twice	5 *μ*g/50*μ*l	62%	7	?	?	?	?	?	?	?	?
		I.p.	Every two days	60 *μ*g/300*μ*l	61%									
[21]	TP508	Topic	Single	0.03 *μ*g	47%	7	?	?	?	?	?	?	?	?
				0.1 *μ*g	39%	10								
				0.3 *%*g	79%	7								
				0.4 *μ*g	37%	10								
				1 *μ*g	43%	10								
				1 *μ*g	78%	7								
				3 *μ*g	22%	7								
				5 *μ*g	?	?								
[22]	TP508	Topic	Single	0.1 *μ*g	?	?	?	?	?	?	53%	14	?	?
[23]	HB-107	Topic	Three times per day	100 *μ*g/ml	63%	11	?	?	?	?	?	?	?	?
[15]	Marine collagen peptides (MCP)	Oral	Once daily	2 g/kg	76%	16	?	?	?	?	?	?	?	?
[24]	LL37	Topic	Twice daily	10 *μ*g	?	?	?	?	?	?	?	?	?	?
[25]	AH90	Topic	Twice daily	250 *μ*g/ml	64%	10	?	?	?	?	?	?	?	?
[26]	Pardaxin (GE33)	Topic	?	8 mg/ml	58%	21	85%	17	?	?	?	?	?	?
[27]	Tylotoin	Topic	Twice daily	20 *μ*g/ml	89%	10	?	?	?	?	?	?	?	?
[28]	CW49	Topic	Twice daily	200 *μ*g/ml	64%	8	?	?	23%	8	?	?	?	?
[29]	E1	Topic	Once daily	60 *μ*M	92%	12	?	?	?	?	?	?	?	?
[30]	Tilapia piscidin 4 (TP4)	Topic	?	2 mg/ml	27%	19	29%	19	?	?	?	?	?	?
[12]	Tilapia piscidin 3 (TP3)	Topic	?	2 mg/ml	23%	19	44%	19	?	?	?	?	?	?
[31]	Proinsulin C	S.c.	Continuous	35 pmol/kg per minute	?	?	?	?	67%	10	?	?	?	?
[32]	Camel milk peptide (CMP)	Oral	Once daily	25 mg/kg	?	?	?	?	37%	7	?	?	?	?
[33]	Ghrelin	S.c.	Once daily	50 nmol/kg	17%	14	?	?	?	?	?	?	0%	14
				100 nmol/kg	0%								50%	
				200 nmol/kg	0%								67%	
[34]	Epinecidin-1 (Epi-1)	Topic	Every three days	90 *μ*g/ml	?	?	65%	25	?	?	?	?	?	?
				900 *μ*g/ml			65%							
				9 mg/ml			71%							
[35]	Marine collagen peptides (MCP)	Topic	Once daily	?	86%	21	?	?	?	?	?	?	?	?
[1]	OM‒LV20	Topic	Twice daily	0.5 nM	?	10	?	?	?	?	?	?	?	?
				1 nM	?									
				2.5 nM	?									
				5 nM	?									
				10 nM	?									
				20 nM	50%									
[13]	Cathelicidin-OA1	Topic	Twice daily	10 *μ*M	6%	10	?	?	?	?	?	?	?	?
				20 *μ*M	53%									
				40 *μ*M	66%									
[16]	OA-GL21	Topic	Twice daily	1 *μ*g/ml	-3%	9	?	?	?	?	?	?	?	?
				10 *μ*g/ml	44%									
				100 *μ*g/ml	53%									
[36]	Cathelicidin-NV	Topic	Twice daily	200 *μ*g/ml	91%	10	?	?	?	?	?	?	?	?
[37]	Pollock Collagen Peptide (PCP)	Oral	?	0.5 g/kg	27%	12	?	?	?	?	?	?	?	?
				2 g/kg	48%									
[38]	OA-FF10	Topic	Twice daily	1 *μ*M	52%	8	?	?	?	?	?	?	?	?
				10 *μ*M	52%									
				100 *μ*M	68%									
[39]	Collagen peptides (CP1/CP2)	Oral	Once daily	0.3 g/kg	-27%/-92%	7	?	?	?	?	?	?	?	?
				0.6 g/kg	-20%/-71%									
				0.9 g/kg	47%/-17%									
[40]	OA-GL12	Topic	Twice daily	0.1 nM	13%	10	?	?	?	?	?	?	?	?
				1 nM	44%									
				10 nM	63%									
[41]	Ot-WHP	Topic	Once daily	200 *μ*g/ml	63%	8	?	?	?	?	?	?	?	?
[42]	Active peptides (APs)	Oral	?	0.5 g/kg	60%	14	?	?	?	?	?	?	?	?
				2 g/kg	80%									
[43]	Skin collagen peptide (Ss-SCP/ Tn-SCP)	Oral	?	2 g/kg	59%/45%	12	?	?	?	?	?	?	?	?
[44]	Cathelicidin-DM	I.v.	Once daily	10 mg/kg	?	?	?	?	?	?	?	?	?	?

^∗^Results shown as a percentage of reduction in the average wound area of the groups treated with peptide compared to the control group on a given postinjury day. RF: reference; P: peptides; R: route; A: application; C: concentration; RWS: reduction in wound size; DA: day analyzed; PI: postinjury; ?: not reported or unclear; I.p.: intraperitoneal; S.c.: subcutaneous; I.v.: intravenously; CP1: collagen peptides bands at 10-15 kDa; CP2: collagen peptides <25 kDa; Ss-SCP: *Salmo salar* skin collagen peptides; Tn-SCP: *Tilapia nilotica* skin collagen peptides.

**Table 2:** All relevant results reported in all studies included in the systematic review on peptides of animal origin applied in the treatment of skin wounds.

Peptide source	Outcomes	
	Increased	Reduced
Human [21, 22, 24, 31]	Wound closure [21, 22, 24, 31] Reepithelialization [24] Inflammatory cells [21, 22] Blood vessels [21, 22, 24, 31] Tensile strength [21]	Wound area [21, 22, 24, 31] Inflammatory cells [31] IL-1*β*, IL-6, and TNF-*α* [31]

Other mammals [20, 29, 32, 33]	Wound closure [20, 29, 32, 33] Reepithelialization [20, 29] Dermal regeneration [20, 32] Inflammatory cells [32] Blood vessels [20, 32, 33] Collagen [20, 29, 32, 33] SOD, CAT, GSH, and MIF [32] Hexosamine [29, 33] Ascorbate and Proteins [29] Tensile strength [29] Collagen contraction temperature [29] DNA, NO, VEGF, and TGF-*β*1 [33]	Wound area [20, 29, 32, 33] MDA, TNF-*α*, and NF-kB [32] Lipid peroxidation [29]

Amphibian [1, 13, 16, 25, 27, 28, 36, 38, 40, 41, 44]	Wound closure [1, 13, 16, 25, 27, 28, 36, 38, 40, 41, 44] Reepithelialization [13, 16, 25, 27, 28, 36, 40, 41] Dermal regeneration [13, 16, 25, 27, 28, 36, 40, 41] Inflammatory cells [13, 27, 41] Blood vessels [28] Collagen [36, 41] Myofibroblasts [25, 27, 36, 41] MCP-1 and VEGF [36] TNF-*α* [36, 41] TGF-*β* [41] TGF-*β*1 [13, 27, 36] CXCL1 and CCL2 [41] HIF-1*α*, eNOS, and iNOS in diabetic wounds [28]	Wound area [1, 13, 16, 25, 27, 28, 36, 38, 40, 41, 44] Inflammatory cells [28] IL-6 and TNF-*α* in diabetic wounds [28]

Fish [12, 15, 26, 30, 34, 35, 37, 43]	Wound closure [12, 15, 26, 30, 34, 35, 37, 43] Reepithelialization [12, 26, 34, 35, 37, 43] Dermal regeneration [12, 26, 34, 35, 37, 43] Inflammatory cells [26, 34] Collagen [34, 37, 43] VEGF [26, 30, 43] EGF and TGF-*β* [30, 37] FGF [43] bFGF [37] T*β*RII [37] IL-1 [30] IL-10 [43] NOD2 and BD14 [43] Hydroxyproline [37]	Wound area [12, 15, 26, 30, 34, 35, 37, 43] Inflammatory cells [12] IL-6 [12, 26, 30, 34] TNF [30] MCP-1 [26] TNF-*α* [12, 26] CRP [34] CXCL5 [12] Bacterial loads [12, 26, 34]

Jellyfish [39]	Wound closure, Reepithelialization, Dermal regeneration, Collagen, *β*-FGF, and TGF-*β*1	Wound area

Mollusk [42]	Wound closure, Reepithelialization, Dermal regeneration, CD31, EGF, FGF, TGF-*β*, T*β*RII, IL-1, and IL-10	Wound area, Inflammatory cells, and Smad7

Insect [23]	Wound closure, Reepithelialization, and Inflammatory cells	Wound area

IL: interleukin; TNF: tumor necrosis factor; SOD: superoxide dismutase; CAT: catalase; GSH: glutathione; MIF: macrophage migration inhibitory factor; DNA: deoxyribonucleic acid; NO: nitric oxide; VEGF: vascular endothelial growth factor; TGF: transforming growth factor; MDA: malondialdehyde; NF-*κ*B: transcription factor kappa-B; MCP: monocyte chemoattractant protein; HIF: hypoxia-inducible factor; eNOS: endothelial nitric oxide synthase; iNOS: inducible nitric oxide synthase; EGF: epidermal growth factor; CRP: C-reactive protein; FGF: fibroblast growth factor; T*β*R: transforming growth factor-*β* receptor.

## Supplementary Materials

Supplementary Materials S1 Table: complete search strategy with search filters and number of research records recovered in the PubMed-Medline, Scopus, and Web of Science databases. ^∗^: In the PubMed-Medline database, standardized animal filters were obtained in “Hooijmans CR, Tillema A, Leenaars M, Ritskes-Hoitinga M. Enhancing search efficiency by means of a search filter for finding all studies on animal experimentation in PubMed. Laboratory Animals 2010;44:170-175.”. S2 Table: studies excluded during the process of eligibility. S3 Table: general characteristics of the preclinical models used in all studies investigating the relevance of animal peptides in the treatment of skin wounds. ♂: male; ♀: female; ?: not reported or unclear; wk: weeks. S4 Table: general characteristics of skin wounds used in preclinical models investigating the relevance of animal peptides as healing agents. ?: not reported or unclear; *S. aureus*: *Staphylococcus aureus*; *E. coli: Escherichia coli*; D: diameter; CFU: colony-forming unit. S5 Table: description of the main characteristics related to peptides included in the systematic review on peptides of animal origin applied in the treatment of skin wounds. S6 Table: treatment protocols used in all studies investigating the relevance of animal peptides in the treatment of skin wounds. ?: not reported or unclear; SAL: saline solution; PBS: phosphate-buffered saline solution; DPBS: Dulbecco's phosphate-buffered saline; I.p.: intraperitoneal; S.c.: subcutaneous; I.v.: intravenously. S7 Table: PRISMA 2009 Checklist. *From*: Moher D, Liberati A, Tetzlaff J, Altman DG, The PRISMA Group (2009). Preferred Reporting Items for Systematic Reviews and Meta-Analyses: The PRISMA Statement. PLoS Med 6(7): e1000097. doi:10.1371/journal.pmed1000097.
