# Supplementary material for: Peptides from Animal Origin: A Systematic Review on Biological Sources and Effects on Skin Wounds
Source: Oxid Med Cell Longev. 2020 Oct 23;2020:4352761. doi: 10.1155/2020/4352761 (PMC7603624; doi:10.1155/2020/4352761)
Supplement: Supplementary Materials — S1 Table: complete search strategy with search filters and number of research records recovered in the PubMed-Medline, Scopus, and Web of Science databases. ∗: In the PubMed-Medline database, standardized animal filters were obtained in “Hooijmans CR, Tillema A, Leenaars M, Ritskes-Hoitinga M. Enhancing search efficiency by means of a search filter for finding all studies on animal experimentation in PubMed. Laboratory Animals 2010;44:170-175.”. S2 Table: studies excluded during the process of eligibility. S3 Table: general characteristics of the preclinical models used in all studies investigating the relevance of animal peptides in the treatment of skin wounds. ♂: male; ♀: female; ?: not reported or unclear; wk: weeks. S4 Table: general characteristics of skin wounds used in preclinical models investigating the relevance of animal peptides as healing agents. ?: not reported or unclear; S. aureus: Staphylococcus aureus; E. coli: Escherichia coli; D: diameter; CFU: colony-forming unit. S5 Table: description of the main characteristics related to peptides included in the systematic review on peptides of animal origin applied in the treatment of skin wounds. S6 Table: treatment protocols used in all studies investigating the relevance of animal peptides in the treatment of skin wounds. ?: not reported or unclear; SAL: saline solution; PBS: phosphate-buffered saline solution; DPBS: Dulbecco's phosphate-buffered saline; I.p.: intraperitoneal; S.c.: subcutaneous; I.v.: intravenously. S7 Table: PRISMA 2009 Checklist. From: Moher D, Liberati A, Tetzlaff J, Altman DG, The PRISMA Group (2009). Preferred Reporting Items for Systematic Reviews and Meta-Analyses: The PRISMA Statement. PLoS Med 6(7): e1000097. doi:10.1371/journal.pmed1000097. [file 4352761.f1.zip › S3 Table.docx]

S3 Table. General characteristics of the preclinical models used in all studies investigating the relevance of animal peptides in the treatment of skin wounds.

| **Reference** | **Animal models** | | | | | |
| --- | --- | --- | --- | --- | --- | --- |
|  | **Animal** | **Strain** | **Sex** | **Age** | **Weight** | **Associated pathology** |
| [20] | Rat | ? | ? | ? | ? | No |
| [21] | Rat | Sprague-Dawley | ♂ | ? | 250-300 g | No |
| [22] | Rat | Sprague-Dawley | ♂ | ? | 280-340 g | Ischemia |
| [23] | Mice | C57BL/6 | ? | ? | ? | No |
| [15] | Rat | Sprague-Dawley | ♂ | ? | 230-250 g | No |
| [24] | Mice | C57BL/6 | ? | 8-12 wk | ? | No |
| [25] | Mice | Balb/C | ♂ | ? | ? | No |
| [26] | Mice | Balb/C | ♀ | 6-8 wk | ? | No |
| [27] | Mice | Kunming | ♂ | 6-8 wk | ? | No |
| [28] | Mice | Balb/C - *db*/*db* | ♂ | 8 wk | ? | Diabetes |
| [29] | Rat | Wistar | ♂ | 43 wk | 500-600 g | No |
| [30] | Mice | Balb/C | ♀ | 6-8 wk | ? | No |
| [12] | Mice | Balb/C | ♀ | 6-8 wk | ? | No |
| [31] | Mice | C57BL/6 | ♂ | 6 wk | ? | Diabetes |
| [32] | Rat | Sprague-Dawley | ♂ | 8 wk | 150-200 g | Diabetes |
| [33] | Mice | Kunming | ♂ | 7-8 wk | ? | No |
| [34] | Pig | Yorkshire | ? | 6 wk | 10-13 kg | No |
| [35] | Rabbit | ? | ♂♀ | ? | ? | No |
| [1] | Mice | ? | ♂ | ? | 22-25 g | No |
| [13] | Mice | ? | ♂ | ? | 22-25 g | No |
| [16] | Mice | ? | ♂ | ? | 22-25 g | No |
| [36] | Mice | Kunming | ♂ | 6-7 wk | ? | No |
| [37] | Rat | Sprague-Dawley | ♂ | ? | 160-170 g | No |
| [38] | Mice | ? | ♂ | ? | 20-25 g | No |
| [39] | Mice | ? | ♂ | ? | 26 g | No |
| [40] | Mice | ? | ♂ | ? | 22-25 g | No |
| [41] | Mice | Balb/C | ♀ | ? | 18-20 g | No |
| [42] | Mice | ? | ? | ? | ? | No |
| [43] | Rat | Sprague-Dawley | ♂ | ? | 150-170 g | No |
| [44] | Mice | Kunming | ♀ | 6 wk | 27-30 g | No |

♂: Male, ♀: Female, ?: Not reported or unclear, wk: Weeks.
