# Supplementary material for: Peptides from Animal Origin: A Systematic Review on Biological Sources and Effects on Skin Wounds
Source: Oxid Med Cell Longev. 2020 Oct 23;2020:4352761. doi: 10.1155/2020/4352761 (PMC7603624; doi:10.1155/2020/4352761)
Supplement: Supplementary Materials — S1 Table: complete search strategy with search filters and number of research records recovered in the PubMed-Medline, Scopus, and Web of Science databases. ∗: In the PubMed-Medline database, standardized animal filters were obtained in “Hooijmans CR, Tillema A, Leenaars M, Ritskes-Hoitinga M. Enhancing search efficiency by means of a search filter for finding all studies on animal experimentation in PubMed. Laboratory Animals 2010;44:170-175.”. S2 Table: studies excluded during the process of eligibility. S3 Table: general characteristics of the preclinical models used in all studies investigating the relevance of animal peptides in the treatment of skin wounds. ♂: male; ♀: female; ?: not reported or unclear; wk: weeks. S4 Table: general characteristics of skin wounds used in preclinical models investigating the relevance of animal peptides as healing agents. ?: not reported or unclear; S. aureus: Staphylococcus aureus; E. coli: Escherichia coli; D: diameter; CFU: colony-forming unit. S5 Table: description of the main characteristics related to peptides included in the systematic review on peptides of animal origin applied in the treatment of skin wounds. S6 Table: treatment protocols used in all studies investigating the relevance of animal peptides in the treatment of skin wounds. ?: not reported or unclear; SAL: saline solution; PBS: phosphate-buffered saline solution; DPBS: Dulbecco's phosphate-buffered saline; I.p.: intraperitoneal; S.c.: subcutaneous; I.v.: intravenously. S7 Table: PRISMA 2009 Checklist. From: Moher D, Liberati A, Tetzlaff J, Altman DG, The PRISMA Group (2009). Preferred Reporting Items for Systematic Reviews and Meta-Analyses: The PRISMA Statement. PLoS Med 6(7): e1000097. doi:10.1371/journal.pmed1000097. [file 4352761.f1.zip › S4 Table.docx]

S4 Table. General characteristics of skin wounds used in preclinical models investigating the relevance of animal peptides as healing agents.

| **Reference** | **Cutaneous wounds** | | | | |
| --- | --- | --- | --- | --- | --- |
|  | **Lesion** | **Site** | **Initial area** | **Number** | **Infection/**  **Concentration** |
| [20] | Excision | Dorsum | 8 mm D | 6 | Uninfected |
| [21] | Excision | Dorsum | 2 cm D | 2 | Uninfected |
| [22] | Excision | Dorsum | 2 cm D | 2-4 | Uninfected |
| [23] | Excision | Dorsum | 3 mm D | 1 | Uninfected |
| [15] | Excision | Dorsum | 2 cm D | 2 | Uninfected |
| [24] | Excision | Dorsum | 5 mm D | 2 | Uninfected |
| [25] | Excision | Dorsum | 7 mm D | ? | Uninfected |
| [26] | Excision | Abdomen | 1 cm D | 1 | *S. aureus/*10^6^ CFU |
| [27] | Excision | Dorsum | 9 mm D | 2 | Uninfected |
| [28] | Excision | Dorsum | 7 mm D | ? | Uninfected |
| [29] | Excision | Dorsum | 2 cm^2^ | 1 | Uninfected |
| [30] | Excision | Dorsum | 1 cm D | 1 | *S. aureus*/10^6^ CFU |
| [12] | Excision | Dorsum | 1 cm D | 1 | *S. aureus*/10^6^ CFU |
| [31] | Excision | Dorsum | 4 mm D | ? | Uninfected |
| [32] | Incision | Dorsum | 2×5 mm | 1 | Uninfected |
| [33] | Excision | Dorsum | 1.44 cm^2^ | 1 | Uninfected |
| [34] | Burn | Dorsum | 3 cm D | 6 | *S. aureus/*10^10^ CFU |
| [35] | Burn | Dorsum | 4 cm^2^ | 2 | Uninfected |
| [1] | Excision | Dorsum | 8×8 mm | 2 | Uninfected |
| [13] | Excision | Dorsum | 8×8 mm | 2 | Uninfected |
| [16] | Excision | Dorsum | 8×8 mm | 2 | Uninfected |
| [36] | Excision | Dorsum | 6 mm D | 2 | Uninfected |
| [37] | Excision | Dorsum | 1 cm D | 1 | Uninfected |
| [38] | Excision | Dorsum | 8×8 mm | 2 | Uninfected |
| [39] | Excision | Dorsum | 8.5 mm D | 1 | Uninfected |
| [40] | Excision | Dorsum | 8×8 mm | 2 | Uninfected |
| [41] | Excision | Dorsum | 8 mm D | 1 | Uninfected |
| [42] | Excision | Dorsum | 0.8 cm D | 1 | Uninfected |
| [43] | Excision | Dorsum | 1 cm | 1 | Uninfected |
| [44] | Excision | Dorsum | 1 cm^2^ | 1 | *E. coli*/2 x 10^5^ CFU |

?: Not reported or unclear, *S. aureus*: *Staphylococcus aureus*, *E. coli: Escherichia coli*, D: Diameter, CFU: Colony forming unit.
