# Supplementary material for: Peptides from Animal Origin: A Systematic Review on Biological Sources and Effects on Skin Wounds
Source: Oxid Med Cell Longev. 2020 Oct 23;2020:4352761. doi: 10.1155/2020/4352761 (PMC7603624; doi:10.1155/2020/4352761)
Supplement: Supplementary Materials — S1 Table: complete search strategy with search filters and number of research records recovered in the PubMed-Medline, Scopus, and Web of Science databases. ∗: In the PubMed-Medline database, standardized animal filters were obtained in “Hooijmans CR, Tillema A, Leenaars M, Ritskes-Hoitinga M. Enhancing search efficiency by means of a search filter for finding all studies on animal experimentation in PubMed. Laboratory Animals 2010;44:170-175.”. S2 Table: studies excluded during the process of eligibility. S3 Table: general characteristics of the preclinical models used in all studies investigating the relevance of animal peptides in the treatment of skin wounds. ♂: male; ♀: female; ?: not reported or unclear; wk: weeks. S4 Table: general characteristics of skin wounds used in preclinical models investigating the relevance of animal peptides as healing agents. ?: not reported or unclear; S. aureus: Staphylococcus aureus; E. coli: Escherichia coli; D: diameter; CFU: colony-forming unit. S5 Table: description of the main characteristics related to peptides included in the systematic review on peptides of animal origin applied in the treatment of skin wounds. S6 Table: treatment protocols used in all studies investigating the relevance of animal peptides in the treatment of skin wounds. ?: not reported or unclear; SAL: saline solution; PBS: phosphate-buffered saline solution; DPBS: Dulbecco's phosphate-buffered saline; I.p.: intraperitoneal; S.c.: subcutaneous; I.v.: intravenously. S7 Table: PRISMA 2009 Checklist. From: Moher D, Liberati A, Tetzlaff J, Altman DG, The PRISMA Group (2009). Preferred Reporting Items for Systematic Reviews and Meta-Analyses: The PRISMA Statement. PLoS Med 6(7): e1000097. doi:10.1371/journal.pmed1000097. [file 4352761.f1.zip › S5 Table.docx]

S5 Table. Description of the main characteristics related to peptides included in the systematic review on peptides of animal origin applied in the treatment of skin wounds.

|  |  | **Peptides** | | |
| --- | --- | --- | --- | --- |
|  |  |  |  |  |
| **Reference** |  | **Name** | **Origin** | **Sequence** |
| [20] |  | Thymosin β4 | Bovine thymus | ? |
| [21] |  | TP508 | Human thrombin | AGYKPDEGKRGDACEGDSGGPFV |
| [22] |  | TP508 | Human thrombin | AGYKPDEGKRGDACEGDSGGPFV |
| [23] |  | HB-107 | *Hyalophora cecropia* | MPKEKVFLKIEKMGRNIRN |
| [15] |  | Marine collagen peptides (MCP) | *Oncorhynchus keta* | ? |
| [24] |  | LL37 | Human cathelicidin | LLGDFFRKSKEKIGKEFKRIVQRIKDFLRNLVPRTES |
| [25] |  | AH90 | *Odorrana grahami* | ATAWDFGPHGLLPIRPIRIRPLCG |
| [26] |  | Pardaxin (GE33) | *Pardachirus marmoratus* | ? |
| [27] |  | Tylotoin | *Tylototriton verrucosus* | KCVRQNNKRVCK |
| [28] |  | CW49 | *Odorrana grahami* | APFRMGICTTN |
| [29] |  | E1 | Bovine achilles tendon | GETGPAGPAGPIGPVGARGPAGPQGPRGDKGETGEQ |
| [30] |  | Tilapia piscidin 4 (TP4) | *Oreochromis niloticus* | FIHHIIGGLFSAGKAIHRLIRRRRR |
| [12] |  | Tilapia piscidin 3 (TP3) | *Oreochromis niloticus* | FIHHIIGGLFSVGKHIHSLIHGH |
| [31] |  | Proinsulin C | Human proinsulin | ? |
| [32] |  | Camel milk peptide (CMP) | Camel milk | ? |
| [33] |  | Ghrelin | Mouse ghrelin | ? |
| [34] |  | Epinecidin-1 (Epi-1) | *Epinephelus coioides* | GFIFHIIKGLFHAGKMIHGLV |
| [35] |  | Marine collagen peptides (MCP) | *Oreochromis niloticus* | ? |
| [1] |  | OM‐LV20 | *Odorrana margaretae* | LVGKLLKGAVGDVCGLLPIC |
| [13] |  | Cathelicidin-OA1 | *Odorrana andersonii* | IGRDPTWSHLAASCLKCIFDDLPKTHN |
| [16] |  | OA-GL21 | *Odorrana andersonii* | GLLSGHYGRVVSTQSGHYGRG |
| [36] |  | Cathelicidin-NV | *Nanorana ventripunctata* | ARGKKECKDDRCRLLMKRGSFSYV |
| [37] |  | Pollock Collagen Peptide (PCP) | *Theragra chalcogramma* | ? |
| [38] |  | OA-FF10 | *Odorrana andersonii* | FFTTSCRSC |
| [39] |  | Collagen peptides  (CP1/CP2) | *Rhopilema esculentum* | ? |
| [40] |  | OA-GL12 | *Odorrana andersonii* | GLLSGINAEWPC |
| [41] |  | Ot-WHP | *Odorrana tormota* | ATAWDLGPHGIRPLRPIRIRPLCG |
| [42] |  | Active peptides (APs) | *Pinctada martensii* | ? |
| [43] |  | Skin collagen peptide (SCP) | *Salmo salar*  and *Tilapia nilotica* | ? |
| [44] |  | Cathelicidin-DM | *Duttaphrynus melanostictus* | SSRRKPCKGWLCKLKLRGGYTLIGSATNLNRPTYVRA |
