# Supplementary material for: Peptides from Animal Origin: A Systematic Review on Biological Sources and Effects on Skin Wounds
Source: Oxid Med Cell Longev. 2020 Oct 23;2020:4352761. doi: 10.1155/2020/4352761 (PMC7603624; doi:10.1155/2020/4352761)
Supplement: Supplementary Materials — S1 Table: complete search strategy with search filters and number of research records recovered in the PubMed-Medline, Scopus, and Web of Science databases. ∗: In the PubMed-Medline database, standardized animal filters were obtained in “Hooijmans CR, Tillema A, Leenaars M, Ritskes-Hoitinga M. Enhancing search efficiency by means of a search filter for finding all studies on animal experimentation in PubMed. Laboratory Animals 2010;44:170-175.”. S2 Table: studies excluded during the process of eligibility. S3 Table: general characteristics of the preclinical models used in all studies investigating the relevance of animal peptides in the treatment of skin wounds. ♂: male; ♀: female; ?: not reported or unclear; wk: weeks. S4 Table: general characteristics of skin wounds used in preclinical models investigating the relevance of animal peptides as healing agents. ?: not reported or unclear; S. aureus: Staphylococcus aureus; E. coli: Escherichia coli; D: diameter; CFU: colony-forming unit. S5 Table: description of the main characteristics related to peptides included in the systematic review on peptides of animal origin applied in the treatment of skin wounds. S6 Table: treatment protocols used in all studies investigating the relevance of animal peptides in the treatment of skin wounds. ?: not reported or unclear; SAL: saline solution; PBS: phosphate-buffered saline solution; DPBS: Dulbecco's phosphate-buffered saline; I.p.: intraperitoneal; S.c.: subcutaneous; I.v.: intravenously. S7 Table: PRISMA 2009 Checklist. From: Moher D, Liberati A, Tetzlaff J, Altman DG, The PRISMA Group (2009). Preferred Reporting Items for Systematic Reviews and Meta-Analyses: The PRISMA Statement. PLoS Med 6(7): e1000097. doi:10.1371/journal.pmed1000097. [file 4352761.f1.zip › S6 Table.docx]

S6 Table. Treatment protocols used in all studies investigating the relevance of animal peptides in the treatment of skin wounds.

|  |  | **Intervention** | | | | |
| --- | --- | --- | --- | --- | --- | --- |
| **Reference** |  | **Vehicle** | **Route** | **Application** | **Treatment time (days)** | **Concentration** |
| [20] |  | SAL | Topic  I.p. | Twice  Every two days | 7 | 5 μg/50μl  60 μg/300μl |
| [21] |  | SAL | Topic | Single | ? | 0.03, 0.1, 0.3, 0.4, 3 and 5 μg |
| [22] |  | SAL | Topic | Single | ? | 0.1 μg |
| [23] |  | PBS | Topic | Three times  per day | 14 | 100 µg/ml |
| [15] |  | Water | Oral | Once daily | ? | 2 g/kg |
| [24] |  | Water | Topic | Twice daily | 7 | 10 µg |
| [25] |  | DPBS | Topic | Twice daily | ? | 250 μg/ml |
| [26] |  | PBS | Topic | ? | ? | 8 mg/ml |
| [27] |  | PBS | Topic | Twice daily | ? | 20 μg/ml |
| [28] |  | DPBS | Topic | Twice daily | ? | 200 μg/ml |
| [29] |  | SAL | Topic | Once daily | 12 | 60 µM |
| [30] |  | PBS | Topic | ? | ? | 2 mg/ml |
| [12] |  | PBS | Topic | ? | ? | 2 mg/ml |
| [31] |  | PBS | S.c. | Continuous | 14 | 35 pmol/kg per minute |
| [32] |  | ? | Oral | Once daily | 7 | 25 mg/kg |
| [33] |  | PBS | S.c. | Once daily | 7 | 50, 100 and 200 nmol/kg |
| [34] |  | PBS | Topic | Every three days | 25 | 90, 900 μg/ml and  9 mg/ml |
| [35] |  | ? | Topic | Once daily | 28 | ? |
| [1] |  | SAL | Topic | Twice daily | ? | 0.5, 1, 2.5, 5, 10 and 20 nM |
| [13] |  | SAL | Topic | Twice daily | ? | 10, 20 and 40 μM |
| [16] |  | SAL | Topic | Twice daily | ? | 1, 10 and 100 µg/ml |
| [36] |  | ? | Topic | Twice daily | ? | 200 µg/ml |
| [37] |  | SAL | Oral | ? | 7 | 0.5 and 2 g/kg |
| [38] |  | SAL | Topic | Twice daily | ? | 1, 10 and 100 μM |
| [39] |  | SAL | Oral | Once daily | 6 | 0.3, 0.6 and 0.9 g/kg |
| [40] |  | SAL | Topic | Twice daily | ? | 0.1, 1 and 10 nM |
| [41] |  | PBS | Topic | Once daily | 8 | 200 µg/ml |
| [42] |  | SAL | Oral | ? | ? | 0.5 and 2 g/kg |
| [43] |  | SAL | Oral | ? | ? | 2 g/kg |
| [44] |  | PBS | I.v. | Once daily | 5 | 10 mg/kg |

?: Not reported or unclear, SAL: Saline solution, PBS: Phosphate buffered saline solution, DPBS: Dulbecco's phosphate-buffered saline, I.p.: Intraperitoneal, S.c.: Subcutaneous, I.v.: Intravenously.
