# Supplementary material for: Peptides from Animal Origin: A Systematic Review on Biological Sources and Effects on Skin Wounds
Source: Oxid Med Cell Longev. 2020 Oct 23;2020:4352761. doi: 10.1155/2020/4352761 (PMC7603624; doi:10.1155/2020/4352761)
Supplement: Supplementary Materials — S1 Table: complete search strategy with search filters and number of research records recovered in the PubMed-Medline, Scopus, and Web of Science databases. ∗: In the PubMed-Medline database, standardized animal filters were obtained in “Hooijmans CR, Tillema A, Leenaars M, Ritskes-Hoitinga M. Enhancing search efficiency by means of a search filter for finding all studies on animal experimentation in PubMed. Laboratory Animals 2010;44:170-175.”. S2 Table: studies excluded during the process of eligibility. S3 Table: general characteristics of the preclinical models used in all studies investigating the relevance of animal peptides in the treatment of skin wounds. ♂: male; ♀: female; ?: not reported or unclear; wk: weeks. S4 Table: general characteristics of skin wounds used in preclinical models investigating the relevance of animal peptides as healing agents. ?: not reported or unclear; S. aureus: Staphylococcus aureus; E. coli: Escherichia coli; D: diameter; CFU: colony-forming unit. S5 Table: description of the main characteristics related to peptides included in the systematic review on peptides of animal origin applied in the treatment of skin wounds. S6 Table: treatment protocols used in all studies investigating the relevance of animal peptides in the treatment of skin wounds. ?: not reported or unclear; SAL: saline solution; PBS: phosphate-buffered saline solution; DPBS: Dulbecco's phosphate-buffered saline; I.p.: intraperitoneal; S.c.: subcutaneous; I.v.: intravenously. S7 Table: PRISMA 2009 Checklist. From: Moher D, Liberati A, Tetzlaff J, Altman DG, The PRISMA Group (2009). Preferred Reporting Items for Systematic Reviews and Meta-Analyses: The PRISMA Statement. PLoS Med 6(7): e1000097. doi:10.1371/journal.pmed1000097. [file 4352761.f1.zip › S1 Table.docx]

S1 Table. Complete search strategy with search filters and number of research records recovered in the PubMed-Medline, Scopus and Web of Science databases.

| **PubMed-MEDLINE - Search Filters** | **Retrieved records** |
| --- | --- |
| **#1 Peptides**  ("peptides"[TIAB] OR "antioxidant peptides"[TIAB] OR "antimicrobial peptides"[TIAB] OR "angiogenic peptides"[TIAB] OR "animal peptides"[TIAB] OR "natural peptides"[TIAB] OR "bioactive peptides"[TIAB] OR "biological peptides"[TIAB] OR "isolated peptides"[TIAB] OR "extracted peptides"[TIAB]) | **228261** |
| **#2 Wound Healing**  ("wound healing"[MeSH Terms] OR "regeneration"[MeSH Terms] OR "regeneration"[TIAB] OR "wound healing"[TIAB] OR "skin repair"[TIAB] OR "cutaneous repair"[TIAB] OR "skin healing"[TIAB] OR "cutaneous healing"[TIAB]) | **337601** |
| **#3 Skin**  ("skin"[MeSH Terms] OR "dermis"[MeSH Terms] OR "epidermis"[MeSH Terms] OR "subcutaneous tissue"[MeSH Terms] OR "granulation tissue"[MeSH Terms] OR "keratinocytes"[MeSH Terms] OR "fibroblasts"[MeSH Terms] OR "integumentary system"[MeSH Terms] OR "skin"[TIAB] OR "dermis"[TIAB] OR "epidermis"[TIAB] OR "subcutaneous tissue"[TIAB] OR "hypodermis"[TIAB] OR "granulation tissue"[TIAB] OR "keratinocytes"[TIAB] OR "fibroblasts"[TIAB] OR "integumentary system"[TIAB] OR "skin injuries"[TIAB] OR "skin fibrosis"[TIAB] OR "skin scars"[TIAB]) | **955157** |
| **#4 First animal filter***  ("animal experimentation"[MeSH Terms] OR "models, animal"[MeSH Terms] OR "invertebrates"[MeSH Terms] OR "animals"[Mesh:noexp] OR "animal population groups"[MeSH Terms] OR "chordata"[MeSH Terms:noexp] OR "chordata, nonvertebrate"[MeSH Terms] OR "vertebrates"[MeSH Terms:noexp] OR "amphibians"[MeSH Terms] OR "birds"[MeSH Terms] OR "fishes"[MeSH Terms] OR "reptiles"[MeSH Terms] OR "mammals"[MeSH Terms:noexp] OR "primates"[MeSH Terms:noexp] OR "artiodactyla"[MeSH Terms] OR "carnivora"[MeSH Terms] OR "cetacea"[MeSH Terms] OR "chiroptera"[MeSH Terms] OR "elephants"[MeSH Terms] OR "hyraxes"[MeSH Terms] OR "insectivora"[MeSH Terms] OR "lagomorpha"[MeSH Terms] OR "marsupialia"[MeSH Terms] OR "monotremata"[MeSH Terms] OR "perissodactyla"[MeSH Terms] OR "rodentia"[MeSH Terms] OR "scandentia"[MeSH Terms] OR "sirenia"[MeSH Terms] OR "xenarthra"[MeSH Terms] OR "haplorhini"[MeSH Terms:noexp] OR "strepsirhini"[MeSH Terms] OR "platyrrhini"[MeSH Terms] OR "tarsii"[MeSH Terms] OR "catarrhini"[MeSH Terms:noexp] OR "cercopithecidae"[MeSH Terms] OR "hylobatidae"[MeSH Terms] OR "hominidae"[MeSH Terms:noexp] OR "gorilla gorilla"[MeSH Terms] OR "pan paniscus"[MeSH Terms] OR "pan troglodytes"[MeSH Terms] OR "pongo pygmaeus"[MeSH Terms]) | **6707109** |
| **#5 Second animal filter***  ("animals"[TIAB] OR "animal"[TIAB] OR "mice"[TIAB] OR "mus"[TIAB] OR "mouse"[TIAB] OR "murine"[TIAB] OR "woodmouse"[TIAB] OR "rats"[TIAB] OR "rat"[TIAB] OR "murinae"[TIAB] OR "muridae"[TIAB] OR "cottonrat"[TIAB] OR "cottonrats"[TIAB] OR "hamster"[TIAB] OR "hamsters"[TIAB] OR "cricetinae"[TIAB] OR "rodentia"[TIAB] OR "rodent"[TIAB] OR "rodents"[TIAB] OR "pigs"[TIAB] OR "pig"[TIAB] OR "swine"[TIAB] OR "swines"[TIAB] OR "piglets"[TIAB] OR "piglet"[TIAB] OR "boar"[TIAB] OR "boars"[TIAB] OR "sus scrofa"[TIAB] OR "ferrets"[TIAB] OR "ferret"[TIAB] OR "polecat"[TIAB] OR "polecats"[TIAB] OR "mustela putorius"[TIAB] OR "guinea pigs"[TIAB] OR "guinea pig"[TIAB] OR "cavia"[TIAB] OR "callithrix"[TIAB] OR "marmoset"[TIAB] OR "marmosets"[TIAB] OR "cebuella"[TIAB] OR "hapale"[TIAB] OR "octodon"[TIAB] OR "chinchilla"[TIAB] OR "chinchillas"[TIAB] OR "gerbillinae"[TIAB] OR "gerbil"[TIAB] OR "gerbils"[TIAB] OR "jird"[TIAB] OR "jirds"[TIAB] OR "merione"[TIAB] OR "meriones"[TIAB] OR "rabbits"[TIAB] OR "rabbit"[TIAB] OR "hares"[TIAB] OR "hare"[TIAB] OR "diptera"[TIAB] OR "flies"[TIAB] OR "fly"[TIAB] OR "dipteral"[TIAB] OR "drosphila"[TIAB] OR "drosophilidae"[TIAB] OR "cats"[TIAB] OR "cat"[TIAB] OR "carus"[TIAB] OR "felis"[TIAB] OR "nematoda"[TIAB] OR "nematode"[TIAB] OR "nematoda"[TIAB] OR "nematode"[TIAB] OR "nematodes"[TIAB] OR "sipunculida"[TIAB] OR "dogs"[TIAB] OR "dog"[TIAB] OR "canine"[TIAB] OR "canines"[TIAB] OR "canis"[TIAB] OR "sheep"[TIAB] OR "sheeps"[TIAB] OR "mouflon"[TIAB] OR "mouflons"[TIAB] OR "ovis"[TIAB] OR "goats"[TIAB] OR "goat"[TIAB] OR "capra"[TIAB] OR "capras"[TIAB] OR "rupicapra"[TIAB] OR "chamois"[TIAB] OR "haplorhini"[TIAB] OR "monkey"[TIAB] OR "monkeys"[TIAB] OR "anthropoidea"[TIAB] OR "anthropoids"[TIAB] OR "saguinus"[TIAB] OR "tamarin"[TIAB] OR "tamarins"[TIAB] OR "leontopithecus"[TIAB] OR "hominidae"[TIAB] OR "ape"[TIAB] OR "apes"[TIAB] OR "pan"[TIAB] OR "paniscus"[TIAB] OR "pan paniscus"[TIAB] OR "bonobo"[TIAB] OR "bonobos"[TIAB] OR "troglodytes"[TIAB] OR "pan troglodytes"[TIAB] OR "gibbon"[TIAB] OR "gibbons"[TIAB] OR "siamang"[TIAB] OR "siamangs"[TIAB] OR "nomascus"[TIAB] OR "symphalangus"[TIAB] OR "chimpanzee"[TIAB] OR "chimpanzees"[TIAB] OR "prosimians"[TIAB] OR "bush baby"[TIAB] OR "prosimian"[TIAB] OR "bush babies"[TIAB] OR "galagos"[TIAB] OR "galago"[TIAB] OR "pongidae"[TIAB] OR "gorilla"[TIAB] OR "gorillas"[TIAB] OR "pongo"[TIAB] OR "pygmaeus"[TIAB] OR "pongo pygmaeus"[TIAB] OR "orangutans"[TIAB] OR "pygmaeus"[TIAB] OR "lemur"[TIAB] OR "lemurs"[TIAB] OR "lemuridae"[TIAB] OR "horse"[TIAB] OR "horses"[TIAB] OR "pongo"[TIAB] OR "equus"[TIAB] OR "cow"[TIAB] OR "calf"[TIAB] OR "bull"[TIAB] OR "chicken"[TIAB] OR "chickens"[TIAB] OR "gallus"[TIAB] OR "quail"[TIAB] OR "bird"[TIAB] OR "birds"[TIAB] OR "quails"[TIAB] OR "poultry"[TIAB] OR "poultries"[TIAB] OR "fowl"[TIAB] OR "fowls"[TIAB] OR "reptile"[TIAB] OR "reptilia"[TIAB] OR "reptiles"[TIAB] OR "snakes"[TIAB] OR "snake"[TIAB] OR "lizard"[TIAB] OR "lizards"[TIAB] OR "alligator"[TIAB] OR "alligators"[TIAB] OR crocodile[TIAB] OR "crocodiles"[TIAB] OR "turtle"[TIAB] OR "turtles"[TIAB] OR "amphibian"[TIAB]) | **4553894** |
| **#6 Third animal filter***  ("amphibians"[TIAB] OR "amphibia"[TIAB] OR "frog"[TIAB] OR "frogs"[TIAB] OR "bombina"[TIAB] OR "salientia"[TIAB] OR "toad"[TIAB] OR "toads"[TIAB] OR "epidalea calamita"[TIAB] OR "salamander"[TIAB] OR "salamanders"[TIAB] OR "eel"[TIAB] OR "eels"[TIAB] OR "fish"[TIAB] OR "fishes"[TIAB] OR "pisces"[TIAB] OR "catfish"[TIAB] OR "catfishes"[TIAB] OR "siluriformes"[TIAB] OR "arius"[TIAB] OR "heteropneustes"[TIAB] OR "sheatfish"[TIAB] OR "perch"[TIAB] OR "perches"[TIAB] OR "percidae"[TIAB] OR "perca"[TIAB] OR "trout"[TIAB] OR "trouts"[TIAB] OR "char"[TIAB] OR "chars"[TIAB] OR "salvelinus"[TIAB] OR "fathead minnow"[TIAB] OR "minnow"[TIAB] OR "cyprinidae"[TIAB] OR "carps"[TIAB] OR "carp"[TIAB] OR "zebrafish"[TIAB] OR "zebrafishes"[TIAB] OR "goldfish"[TIAB] OR "goldfishes"[TIAB] OR "guppy"[TIAB] OR "guppies"[TIAB] OR "chub"[TIAB] OR "chubs"[TIAB] OR "tinca"[TIAB] OR "barbels"[TIAB] OR "barbus"[TIAB] OR "pimephales"[TIAB] OR "promelas"[TIAB] OR "poecilia reticulata"[TIAB] OR "mullet"[TIAB] OR "mullets"[TIAB] OR "seahorse"[TIAB] OR "seahorses"[TIAB] OR "mugil curema"[TIAB] OR "atlantic cod"[TIAB] OR "shark"[TIAB] OR "sharks"[TIAB] OR "catshark"[TIAB] OR "anguilla"[TIAB] OR "salmonid"[TIAB] OR "salmonids"[TIAB] OR "whitefish"[TIAB] OR "whitefishes"[TIAB] OR "salmon"[TIAB] OR "salmons"[TIAB] OR "sole"[TIAB] OR "solea"[TIAB] OR "sea lamprey"[TIAB] OR "lamprey"[TIAB] OR "lampreys"[TIAB] OR "pumpkinseed"[TIAB] OR "sunfish"[TIAB] OR "sunfishes"[TIAB] OR "tilapia"[TIAB] OR "tilapias"[TIAB] OR "turbot"[TIAB] OR "turbots"[TIAB] OR "flatfish"[TIAB] OR "flatfishes"[TIAB] OR "sciuridae"[TIAB] OR "squirrel"[TIAB] OR "squirrels"[TIAB] OR "chipmunk"[TIAB] OR "chipmunks"[TIAB] OR "suslik"[TIAB] OR "susliks"[TIAB] OR "vole"[TIAB] OR "voles"[TIAB] OR "lemming"[TIAB] OR "lemmings"[TIAB] OR "muskrat"[TIAB] OR "muskrats"[TIAB] OR "lemmus"[TIAB] OR "otter"[TIAB] OR "otters"[TIAB] OR "marten"[TIAB] OR "martens"[TIAB] OR "martes"[TIAB] OR "weasel"[TIAB] OR "badger"[TIAB] OR "badgers"[TIAB] OR "ermine"[TIAB] OR "mink"[TIAB] OR "minks"[TIAB] OR "sable"[TIAB] OR "sables"[TIAB] OR "gulo"[TIAB] OR "gulos"[TIAB] OR "wolverine"[TIAB] OR "wolverines"[TIAB] OR "minks"[TIAB] OR "mustela"[TIAB] OR "llama"[TIAB] OR "llamas"[TIAB] OR "alpaca"[TIAB] OR "alpacas"[TIAB] OR "camelid"[TIAB] OR "camelids"[TIAB] OR "guanaco"[TIAB] OR "guanacos"[TIAB] OR "chiroptera"[TIAB] OR "chiropteras"[TIAB] OR "bat"[TIAB] OR "bats"[TIAB] OR "fox"[TIAB] OR "foxes"[TIAB] OR "iguana"[TIAB] OR "iguanas"[TIAB] OR "xenopus laevis"[TIAB] OR "parakeet"[TIAB] OR "parakeets"[TIAB] OR "parrot"[TIAB] OR "parrots"[TIAB] OR "donkey"[TIAB] OR "donkeys"[TIAB] OR "mule"[TIAB] OR "mules"[TIAB] OR "zebra"[TIAB] OR "zebras"[TIAB] OR "shrew"[TIAB] OR "shrews"[TIAB] OR "bison"[TIAB] OR "bisons"[TIAB] OR "buffalo"[TIAB] OR "buffaloes"[TIAB] OR "deer"[TIAB] OR "deers"[TIAB] OR "bear"[TIAB] OR "bears"[TIAB] OR "panda"[TIAB] OR "pandas"[TIAB] OR "wild hog"[TIAB] OR "wild boar"[TIAB] OR "fitchew"[TIAB] OR "fitch"[TIAB] OR "beaver"[TIAB] OR "beavers"[TIAB] OR "jerboa"[TIAB] OR "jerboas"[TIAB] OR "capybara"[TIAB] OR "capybaras"[TIAB]) | **499752** |
| **Combined search:** (((((#6) OR (#5)) OR (#4)) AND (#3)) AND (#2)) AND (#1) | **422** |
|  |  |
| **SCOPUS - Search Filters** | **Retrieved records** |
| **#1 Peptides**  (TITLE-ABS-KEY("peptides") OR TITLE-ABS-KEY("antioxidant peptides") OR TITLE-ABS-KEY ("antimicrobial peptides") OR TITLE-ABS-KEY("angiogenic peptides") OR TITLE-ABS-KEY ("animal peptides") OR TITLE-ABS-KEY("natural peptides") OR TITLE-ABS-KEY("bioactive peptides") OR TITLE-ABS-KEY("biological peptides") OR TITLE-ABS-KEY("isolated peptides") OR TITLE-ABS-KEY("extracted peptides")) | **938519** |
| **#2 Wound Healing**  (TITLE-ABS-KEY("wound healing") OR TITLE-ABS-KEY("regeneration") OR TITLE-ABS-KEY("skin repair") OR TITLE-ABS-KEY("cutaneous repair") OR TITLE-ABS-KEY("skin healing") OR TITLE-ABS-KEY("cutaneous healing")) | **482869** |
| **#3 Skin**  (TITLE-ABS-KEY("skin") OR TITLE-ABS-KEY("dermis") OR TITLE-ABS-KEY ("epidermis") OR TITLE-ABS-KEY("subcutaneous tissue") OR TITLE-ABS-KEY("hypodermis") OR TITLE-ABS-KEY(“granulation tissue") OR TITLE-ABS-KEY("keratinocytes") OR TITLE-ABS-KEY("fibroblasts”) OR TITLE-ABS-KEY ("integumentary system") OR TITLE-ABS-KEY("skin injuries") OR TITLE-ABS-KEY("skin fibrosis") OR TITLE-ABS-KEY("skin scars")) | **1599969** |
| **Combined search:** #3 AND #2 AND #1 | **3908** |
| **Search limits (Keyword):** Animal Experiment and English | **926** |
|  |  |
| **Web of Science - Search Filters** | **Retrieved records** |
| **#1 Peptides**  TS=("peptides" OR "antioxidant peptides" OR "antimicrobial peptides" OR "angiogenic peptides" OR "animal peptides" OR "natural peptides" OR "bioactive peptides" OR "biological peptides" OR "isolated peptides" OR "extracted peptides") | **295819** |
| **#2 Wound Healing**  TS=("wound healing" OR "regeneration" OR "skin repair" OR "cutaneous repair" OR "skin healing" OR "cutaneous healing") | **311314** |
| **#3 Skin**  TS=("skin" OR "dermis" OR "epidermis" OR "subcutaneous tissue" OR "hypodermis" OR "granulation tissue" OR "keratinocytes" OR "fibroblasts" OR "integumentary system" OR "skin injuries" OR "skin fibrosis" OR "skin scars") | **788902** |
| **#4 First animal filter***  TS=("animal experimentation" OR "models, animal" OR “invertebrates" OR "animals" OR "animal population groups" OR "chordata" OR "chordata, nonvertebrate" OR "vertebrates" OR "amphibians" OR "birds" OR "fishes" OR "reptiles" OR "mammals" OR "primates" OR "artiodactyla" OR "carnivora" OR "cetacea" OR "chiroptera" OR "elephants" OR "hyraxes" OR "insectivora" OR "lagomorpha" OR "marsupialia" OR "monotremata" OR "perissodactyla" OR "rodentia" OR "scandentia" OR "sirenia" OR "xenarthra" OR "haplorhini" OR "strepsirhini" OR "platyrrhini" OR "tarsii" OR "catarrhini" OR "cercopithecidae" OR "hylobatidae" OR "hominidae" OR "gorilla gorilla" OR "pan paniscus" OR "pan troglodytes" OR "pongo pygmaeus") | **1075322** |
| **#5 Second animal filter***  TS=("animals" OR "animal" OR "mice" OR "mus" OR "mouse" OR "murine" OR "woodmouse" OR "rats" OR "rat" OR "murinae" OR "muridae" OR "cottonrat" OR "cottonrats" OR "hamster" OR "hamsters" OR "cricetinae" OR "rodentia" OR "rodent" OR "rodents" OR "pigs" OR "pig" OR "swine" OR "swines" OR "piglets" OR "piglet" OR "boar" OR "boars" OR "sus scrofa" OR "ferrets" OR "ferret" OR "polecat" OR "polecats" OR "mustela putorius" OR "guinea pigs" OR "guinea pig" OR "cavia" OR "callithrix" OR "marmoset" OR "marmosets" OR "cebuella" OR "hapale" OR "octodon" OR "chinchilla" OR "chinchillas" OR "gerbillinae" OR "gerbil" OR "gerbils" OR "jird" OR "jirds" OR "merione" OR "meriones" OR "rabbits" OR "rabbit" OR "hares" OR "hare" OR "diptera" OR "flies" OR "fly" OR "dipteral" OR "drosphila" OR "drosophilidae" OR "cats" OR "cat" OR "carus" OR "felis" OR "nematoda" OR "nematode" OR "nematoda" OR "nematode" OR "nematodes" OR "sipunculida" OR "dogs" OR "dog" OR "canine" OR "canines" OR "canis" OR "sheep" OR "sheeps" OR "mouflon" OR "mouflons" OR "ovis" OR "goats" OR "goat" OR "capra" OR "capras" OR "rupicapra" OR "chamois" OR "haplorhini" OR "monkey" OR "monkeys" OR "anthropoidea" OR "anthropoids" OR "saguinus" OR "tamarin" OR "tamarins" OR "leontopithecus" OR "hominidae" OR "ape" OR "apes" OR "pan" OR "paniscus" OR "pan paniscus" OR "bonobo" OR "bonobos" OR "troglodytes" OR "pan troglodytes" OR "gibbon" OR "gibbons" OR "siamang" OR "siamangs" OR "nomascus" OR "symphalangus" OR "chimpanzee" OR "chimpanzees" OR "prosimians" OR "bush baby" OR "prosimian" OR "bush babies" OR "galagos" OR "galago" OR "pongidae" OR "gorilla" OR "gorillas" OR "pongo" OR "pygmaeus" OR "pongo pygmaeus" OR "orangutans" OR "pygmaeus" OR "lemur" OR "lemurs" OR "lemuridae" OR "horse" OR "horses" OR "pongo" OR "equus" OR "cow" OR "calf" OR "bull" OR "chicken" OR "chickens" OR "gallus" OR "quail" OR "bird" OR "birds" OR "quails" OR "poultry" OR "poultries" OR "fowl" OR "fowls" OR "reptile" OR "reptilia" OR "reptiles" OR "snakes" OR "snake" OR "lizard" OR "lizards" OR "alligator" OR "alligators" OR "crocodile" OR "crocodiles" OR "turtle" OR "turtles" OR "amphibian") | **5837275** |
| **#6 Third animal filter***  TS=("amphibians" OR "amphibia" OR "frog" OR "frogs" OR "bombina" OR "salientia" OR "toad" OR "toads" OR "epidalea calamita" OR "salamander" OR "salamanders" OR "eel" OR "eels" OR "fish" OR "fishes" OR "pisces" OR "catfish" OR "catfishes" OR "siluriformes" OR "arius" OR "heteropneustes" OR "sheatfish" OR "perch" OR "perches" OR "percidae" OR "perca" OR "trout" OR "trouts" OR "char" OR "chars" OR "salvelinus" OR "fathead minnow" OR "minnow" OR "cyprinidae" OR "carps" OR "carp" OR "zebrafish" OR "zebrafishes" OR "goldfish" OR "goldfishes" OR "guppy" OR "guppies" OR "chub" OR "chubs" OR "tinca" OR "barbels" OR "barbus" OR "pimephales" OR "promelas" OR "poecilia reticulata" OR "mullet" OR "mullets" OR "seahorse" OR "seahorses" OR "mugil curema" OR "atlantic cod" OR "shark" OR "sharks" OR "catshark" OR "anguilla" OR "salmonid" OR "salmonids" OR "whitefish" OR "whitefishes" OR "salmon" OR "salmons" OR "sole" OR "solea" OR "sea lamprey" OR "lamprey" OR "lampreys" OR "pumpkinseed" OR "sunfish" OR "sunfishes" OR "tilapia" OR "tilapias" OR "turbot" OR "turbots" OR "flatfish" OR "flatfishes" OR "sciuridae" OR "squirrel" OR "squirrels" OR "chipmunk" OR "chipmunks" OR "suslik" OR "susliks" OR "vole" OR "voles" OR "lemming" OR "lemmings" OR "muskrat" OR "muskrats" OR "lemmus" OR "otter" OR "otters" OR "marten" OR "martens" OR "martes" OR "weasel" OR "badger" OR "badgers" OR "ermine" OR "mink" OR "minks" OR "sable" OR "sables" OR "gulo" OR "gulos" OR "wolverine" OR "wolverines" OR "minks" OR "mustela" OR "llama" OR "llamas" OR "alpaca" OR "alpacas" OR "camelid" OR "camelids" OR "guanaco" OR "guanacos" OR "chiroptera" OR "chiropteras" OR "bat" OR "bats" OR "fox" OR "foxes" OR "iguana" OR "iguanas" OR "xenopus laevis" OR "parakeet" OR "parakeets" OR "parrot" OR "parrots" OR "donkey" OR "donkeys" OR "mule" OR "mules" OR "zebra" OR "zebras" OR "shrew" OR "shrews" OR "bison" OR "bisons" OR "buffalo" OR "buffaloes" OR "deer" OR "deers" OR "bear" OR "bears" OR "panda" OR "pandas" OR "wild hog" OR "wild boar" OR "fitchew" OR "fitch" OR "beaver" OR "beavers" OR "jerboa" OR "jerboas" OR "capybara" OR "capybaras") | **1034243** |
| **Combined search:** (((((#6) OR (#5)) OR (#4)) AND (#3)) AND (#2)) AND (#1)  **Search limits (Keyword):** English | **386** |

*: In PubMed-Medline and Web of Science databases, standardized animal filters were obtained in "Hooijmans CR, Tillema A, Leenaars M, Ritskes-Hoitinga M. Enhancing search efficiency by means of a search filter for finding all studies on animal experimentation in PubMed. Laboratory Animals 2010;44:170-175."
