# Supplementary material for: Peptides from Animal Origin: A Systematic Review on Biological Sources and Effects on Skin Wounds
Source: Oxid Med Cell Longev. 2020 Oct 23;2020:4352761. doi: 10.1155/2020/4352761 (PMC7603624; doi:10.1155/2020/4352761)
Supplement: Supplementary Materials — S1 Table: complete search strategy with search filters and number of research records recovered in the PubMed-Medline, Scopus, and Web of Science databases. ∗: In the PubMed-Medline database, standardized animal filters were obtained in “Hooijmans CR, Tillema A, Leenaars M, Ritskes-Hoitinga M. Enhancing search efficiency by means of a search filter for finding all studies on animal experimentation in PubMed. Laboratory Animals 2010;44:170-175.”. S2 Table: studies excluded during the process of eligibility. S3 Table: general characteristics of the preclinical models used in all studies investigating the relevance of animal peptides in the treatment of skin wounds. ♂: male; ♀: female; ?: not reported or unclear; wk: weeks. S4 Table: general characteristics of skin wounds used in preclinical models investigating the relevance of animal peptides as healing agents. ?: not reported or unclear; S. aureus: Staphylococcus aureus; E. coli: Escherichia coli; D: diameter; CFU: colony-forming unit. S5 Table: description of the main characteristics related to peptides included in the systematic review on peptides of animal origin applied in the treatment of skin wounds. S6 Table: treatment protocols used in all studies investigating the relevance of animal peptides in the treatment of skin wounds. ?: not reported or unclear; SAL: saline solution; PBS: phosphate-buffered saline solution; DPBS: Dulbecco's phosphate-buffered saline; I.p.: intraperitoneal; S.c.: subcutaneous; I.v.: intravenously. S7 Table: PRISMA 2009 Checklist. From: Moher D, Liberati A, Tetzlaff J, Altman DG, The PRISMA Group (2009). Preferred Reporting Items for Systematic Reviews and Meta-Analyses: The PRISMA Statement. PLoS Med 6(7): e1000097. doi:10.1371/journal.pmed1000097. [file 4352761.f1.zip › S2 Table.docx]

S2 Table. Studies excluded during the process of eligibility.

| **Exclusion criteria** |  | **Studies** | **Number** |
| --- | --- | --- | --- |
| Association with other compounds |  | \| [Anti-infective biomaterials with surface-decorated tachyplesin I](https://www2.scopus.com/record/display.uri?eid=2-s2.0-85053177564&origin=resultslist&sort=plf-f&src=s&nlo=&nlr=&nls=&mltAll=t&sid=4d2dea855575f6fbdb403e5ce008a978&sot=comb&sdt=cl&cluster=scoexactkeywords%2c%22Animal+Experiment%22%2ct%2bscolang%2c%22English%22%2ct&sl=1001&s=%28%28TITLE-ABS-KEY%28%22peptides%22%29+OR+TITLE-ABS-KEY%28%22antioxidant+peptides%22%29+OR+TITLE-ABS-KEY+%28%22antimicrobial+peptides%22%29+OR+TITLE-ABS-KEY%28%22angiogenic+peptides%22%29+OR+TITLE-ABS-KEY+%28%22animal+peptides%22%29+OR+TITLE-ABS-KEY%28%22natural+peptides%22%29+OR+TITLE-ABS-KEY%28%22bioactive+peptides%22%29+OR+TITLE-ABS-KEY%28%22biological+peptides%22%29+OR+TITLE-ABS-KEY%28%22isolated+peptides%22%29+OR+TITLE-ABS-KEY%28%22extracted+peptides%22%29%29%29+AND+%28%28TITLE-ABS-KEY%28%22wound+healing%22%29+OR+TITLE-ABS-KEY%28%22regeneration%22%29+OR+TITLE-ABS-KEY%28%22skin+repair%22%29+OR+TITLE-ABS-KEY%28%22cutaneous+repair%22%29+OR+TITLE-ABS-KEY%28%22skin+healing%22%29+OR+TITLE-ABS-KEY%28%22cutaneous+healing%22%29%29%29+AND+%28%28TITLE-ABS-KEY%28%22skin%22%29+OR+TITLE-ABS-KEY%28%22dermis%22%29+OR+TITLE-ABS-KEY+%28%22epidermis%22%29+OR+TITLE-ABS-KEY%28%22subcutaneous+tissue%22%29+OR+TITLE-ABS-KEY%28%22hypodermis%22%29+OR+TITLE-ABS-KEY%28%22granulation+tissue%22%29+OR+TITLE-ABS-KEY%28%22keratinocytes%22%29+OR+TITLE-ABS-KEY%28%22fibroblasts%22%29+OR+TITLE-ABS-KEY+%28%22integumentary+system%22%29+OR+TITLE-ABS-KEY%28%22skin+injuries%22%29+OR+TITLE-ABS-KEY%28%22skin+fibrosis%22%29+OR+TITLE-ABS-KEY%28%22skin+scars%22%29%29%29&relpos=52&citeCnt=7&searchTerm=) \| \| --- \| \| [A bio-inspired, microchanneled hydrogel with controlled spacing of cell adhesion ligands regulates 3D spatial organization of cells and tissue](https://www.ncbi.nlm.nih.gov/pubmed/25941779) \| \| A hybrid system of hydrogel/frog egg-like microspheres accelerates wound healing via sustained delivery of RCSPs \| \| A Novel Functionalization of Bioactive Antimicrobial Peptide onto the Nano-CeO2/Reduced Graphene Oxide Cluster Type Biocomposite Wound Dressings for Diabetic Wound Care Management: In Vitro and In Vivo Evaluations \| \| [A peptide-morpholino oligomer conjugate targeting Staphylococcus aureus gyrA mRNA improves healing in an infected mouse cutaneous wound mode](https://www-scopus.ez35.periodicos.capes.gov.br/record/display.uri?eid=2-s2.0-84884151162&origin=resultslist&sort=plf-f&src=s&nlo=&nlr=&nls=&mltAll=t&sid=8f682cda9d1e11ee420f15ef65811fea&sot=comb&sdt=cl&cluster=scoexactkeywords%2c%22Animal+Experiment%22%2ct%2bscolang%2c%22English%22%2ct&sl=1001&s=%28%28TITLE-ABS-KEY%28%22peptides%22%29+OR+TITLE-ABS-KEY%28%22antioxidant+peptides%22%29+OR+TITLE-ABS-KEY+%28%22antimicrobial+peptides%22%29+OR+TITLE-ABS-KEY%28%22angiogenic+peptides%22%29+OR+TITLE-ABS-KEY+%28%22animal+peptides%22%29+OR+TITLE-ABS-KEY%28%22natural+peptides%22%29+OR+TITLE-ABS-KEY%28%22bioactive+peptides%22%29+OR+TITLE-ABS-KEY%28%22biological+peptides%22%29+OR+TITLE-ABS-KEY%28%22isolated+peptides%22%29+OR+TITLE-ABS-KEY%28%22extracted+peptides%22%29%29%29+AND+%28%28TITLE-ABS-KEY%28%22wound+healing%22%29+OR+TITLE-ABS-KEY%28%22regeneration%22%29+OR+TITLE-ABS-KEY%28%22skin+repair%22%29+OR+TITLE-ABS-KEY%28%22cutaneous+repair%22%29+OR+TITLE-ABS-KEY%28%22skin+healing%22%29+OR+TITLE-ABS-KEY%28%22cutaneous+healing%22%29%29%29+AND+%28%28TITLE-ABS-KEY%28%22skin%22%29+OR+TITLE-ABS-KEY%28%22dermis%22%29+OR+TITLE-ABS-KEY+%28%22epidermis%22%29+OR+TITLE-ABS-KEY%28%22subcutaneous+tissue%22%29+OR+TITLE-ABS-KEY%28%22hypodermis%22%29+OR+TITLE-ABS-KEY%28%22granulation+tissue%22%29+OR+TITLE-ABS-KEY%28%22keratinocytes%22%29+OR+TITLE-ABS-KEY%28%22fibroblasts%22%29+OR+TITLE-ABS-KEY+%28%22integumentary+system%22%29+OR+TITLE-ABS-KEY%28%22skin+injuries%22%29+OR+TITLE-ABS-KEY%28%22skin+fibrosis%22%29+OR+TITLE-ABS-KEY%28%22skin+scars%22%29%29%29&relpos=366&citeCnt=22&searchTerm=) \| \| [A therapeutic approach for diabetic wound healing using biotinylated GHK incorporated collagen matrices](https://www-scopus.ez35.periodicos.capes.gov.br/record/display.uri?eid=2-s2.0-33845623411&origin=resultslist&sort=plf-f&src=s&nlo=&nlr=&nls=&mltAll=t&sid=8f682cda9d1e11ee420f15ef65811fea&sot=comb&sdt=cl&cluster=scoexactkeywords%2c%22Animal+Experiment%22%2ct%2bscolang%2c%22English%22%2ct&sl=1001&s=%28%28TITLE-ABS-KEY%28%22peptides%22%29+OR+TITLE-ABS-KEY%28%22antioxidant+peptides%22%29+OR+TITLE-ABS-KEY+%28%22antimicrobial+peptides%22%29+OR+TITLE-ABS-KEY%28%22angiogenic+peptides%22%29+OR+TITLE-ABS-KEY+%28%22animal+peptides%22%29+OR+TITLE-ABS-KEY%28%22natural+peptides%22%29+OR+TITLE-ABS-KEY%28%22bioactive+peptides%22%29+OR+TITLE-ABS-KEY%28%22biological+peptides%22%29+OR+TITLE-ABS-KEY%28%22isolated+peptides%22%29+OR+TITLE-ABS-KEY%28%22extracted+peptides%22%29%29%29+AND+%28%28TITLE-ABS-KEY%28%22wound+healing%22%29+OR+TITLE-ABS-KEY%28%22regeneration%22%29+OR+TITLE-ABS-KEY%28%22skin+repair%22%29+OR+TITLE-ABS-KEY%28%22cutaneous+repair%22%29+OR+TITLE-ABS-KEY%28%22skin+healing%22%29+OR+TITLE-ABS-KEY%28%22cutaneous+healing%22%29%29%29+AND+%28%28TITLE-ABS-KEY%28%22skin%22%29+OR+TITLE-ABS-KEY%28%22dermis%22%29+OR+TITLE-ABS-KEY+%28%22epidermis%22%29+OR+TITLE-ABS-KEY%28%22subcutaneous+tissue%22%29+OR+TITLE-ABS-KEY%28%22hypodermis%22%29+OR+TITLE-ABS-KEY%28%22granulation+tissue%22%29+OR+TITLE-ABS-KEY%28%22keratinocytes%22%29+OR+TITLE-ABS-KEY%28%22fibroblasts%22%29+OR+TITLE-ABS-KEY+%28%22integumentary+system%22%29+OR+TITLE-ABS-KEY%28%22skin+injuries%22%29+OR+TITLE-ABS-KEY%28%22skin+fibrosis%22%29+OR+TITLE-ABS-KEY%28%22skin+scars%22%29%29%29&relpos=611&citeCnt=50&searchTerm=) \| \| [Accelerated healing of excisional skin wounds by PL 14736 in alloxan-hyperglycemic rats](https://www-scopus.ez35.periodicos.capes.gov.br/record/display.uri?eid=2-s2.0-33748333238&origin=resultslist&sort=plf-f&src=s&nlo=&nlr=&nls=&mltAll=t&sid=8f682cda9d1e11ee420f15ef65811fea&sot=comb&sdt=cl&cluster=scoexactkeywords%2c%22Animal+Experiment%22%2ct%2bscolang%2c%22English%22%2ct&sl=1001&s=%28%28TITLE-ABS-KEY%28%22peptides%22%29+OR+TITLE-ABS-KEY%28%22antioxidant+peptides%22%29+OR+TITLE-ABS-KEY+%28%22antimicrobial+peptides%22%29+OR+TITLE-ABS-KEY%28%22angiogenic+peptides%22%29+OR+TITLE-ABS-KEY+%28%22animal+peptides%22%29+OR+TITLE-ABS-KEY%28%22natural+peptides%22%29+OR+TITLE-ABS-KEY%28%22bioactive+peptides%22%29+OR+TITLE-ABS-KEY%28%22biological+peptides%22%29+OR+TITLE-ABS-KEY%28%22isolated+peptides%22%29+OR+TITLE-ABS-KEY%28%22extracted+peptides%22%29%29%29+AND+%28%28TITLE-ABS-KEY%28%22wound+healing%22%29+OR+TITLE-ABS-KEY%28%22regeneration%22%29+OR+TITLE-ABS-KEY%28%22skin+repair%22%29+OR+TITLE-ABS-KEY%28%22cutaneous+repair%22%29+OR+TITLE-ABS-KEY%28%22skin+healing%22%29+OR+TITLE-ABS-KEY%28%22cutaneous+healing%22%29%29%29+AND+%28%28TITLE-ABS-KEY%28%22skin%22%29+OR+TITLE-ABS-KEY%28%22dermis%22%29+OR+TITLE-ABS-KEY+%28%22epidermis%22%29+OR+TITLE-ABS-KEY%28%22subcutaneous+tissue%22%29+OR+TITLE-ABS-KEY%28%22hypodermis%22%29+OR+TITLE-ABS-KEY%28%22granulation+tissue%22%29+OR+TITLE-ABS-KEY%28%22keratinocytes%22%29+OR+TITLE-ABS-KEY%28%22fibroblasts%22%29+OR+TITLE-ABS-KEY+%28%22integumentary+system%22%29+OR+TITLE-ABS-KEY%28%22skin+injuries%22%29+OR+TITLE-ABS-KEY%28%22skin+fibrosis%22%29+OR+TITLE-ABS-KEY%28%22skin+scars%22%29%29%29&relpos=629&citeCnt=21&searchTerm=) \| \| [Acceleration of diabetic wound healing by an angiopoietin peptide mimetic](https://www-scopus.ez35.periodicos.capes.gov.br/record/display.uri?eid=2-s2.0-66249112824&origin=resultslist&sort=plf-f&src=s&nlo=&nlr=&nls=&mltAll=t&sid=8f682cda9d1e11ee420f15ef65811fea&sot=comb&sdt=cl&cluster=scoexactkeywords%2c%22Animal+Experiment%22%2ct%2bscolang%2c%22English%22%2ct&sl=1001&s=%28%28TITLE-ABS-KEY%28%22peptides%22%29+OR+TITLE-ABS-KEY%28%22antioxidant+peptides%22%29+OR+TITLE-ABS-KEY+%28%22antimicrobial+peptides%22%29+OR+TITLE-ABS-KEY%28%22angiogenic+peptides%22%29+OR+TITLE-ABS-KEY+%28%22animal+peptides%22%29+OR+TITLE-ABS-KEY%28%22natural+peptides%22%29+OR+TITLE-ABS-KEY%28%22bioactive+peptides%22%29+OR+TITLE-ABS-KEY%28%22biological+peptides%22%29+OR+TITLE-ABS-KEY%28%22isolated+peptides%22%29+OR+TITLE-ABS-KEY%28%22extracted+peptides%22%29%29%29+AND+%28%28TITLE-ABS-KEY%28%22wound+healing%22%29+OR+TITLE-ABS-KEY%28%22regeneration%22%29+OR+TITLE-ABS-KEY%28%22skin+repair%22%29+OR+TITLE-ABS-KEY%28%22cutaneous+repair%22%29+OR+TITLE-ABS-KEY%28%22skin+healing%22%29+OR+TITLE-ABS-KEY%28%22cutaneous+healing%22%29%29%29+AND+%28%28TITLE-ABS-KEY%28%22skin%22%29+OR+TITLE-ABS-KEY%28%22dermis%22%29+OR+TITLE-ABS-KEY+%28%22epidermis%22%29+OR+TITLE-ABS-KEY%28%22subcutaneous+tissue%22%29+OR+TITLE-ABS-KEY%28%22hypodermis%22%29+OR+TITLE-ABS-KEY%28%22granulation+tissue%22%29+OR+TITLE-ABS-KEY%28%22keratinocytes%22%29+OR+TITLE-ABS-KEY%28%22fibroblasts%22%29+OR+TITLE-ABS-KEY+%28%22integumentary+system%22%29+OR+TITLE-ABS-KEY%28%22skin+injuries%22%29+OR+TITLE-ABS-KEY%28%22skin+fibrosis%22%29+OR+TITLE-ABS-KEY%28%22skin+scars%22%29%29%29&relpos=525&citeCnt=38&searchTerm=) \| \| [An identified antioxidant peptide obtained from ostrich (*Struthio camelus*) egg white protein hydrolysate shows wound healing properties](https://www-scopus.ez35.periodicos.capes.gov.br/record/display.uri?eid=2-s2.0-84930814331&origin=resultslist&sort=plf-f&src=s&nlo=&nlr=&nls=&mltAll=t&sid=8f682cda9d1e11ee420f15ef65811fea&sot=comb&sdt=cl&cluster=scoexactkeywords%2c%22Animal+Experiment%22%2ct%2bscolang%2c%22English%22%2ct&sl=1001&s=%28%28TITLE-ABS-KEY%28%22peptides%22%29+OR+TITLE-ABS-KEY%28%22antioxidant+peptides%22%29+OR+TITLE-ABS-KEY+%28%22antimicrobial+peptides%22%29+OR+TITLE-ABS-KEY%28%22angiogenic+peptides%22%29+OR+TITLE-ABS-KEY+%28%22animal+peptides%22%29+OR+TITLE-ABS-KEY%28%22natural+peptides%22%29+OR+TITLE-ABS-KEY%28%22bioactive+peptides%22%29+OR+TITLE-ABS-KEY%28%22biological+peptides%22%29+OR+TITLE-ABS-KEY%28%22isolated+peptides%22%29+OR+TITLE-ABS-KEY%28%22extracted+peptides%22%29%29%29+AND+%28%28TITLE-ABS-KEY%28%22wound+healing%22%29+OR+TITLE-ABS-KEY%28%22regeneration%22%29+OR+TITLE-ABS-KEY%28%22skin+repair%22%29+OR+TITLE-ABS-KEY%28%22cutaneous+repair%22%29+OR+TITLE-ABS-KEY%28%22skin+healing%22%29+OR+TITLE-ABS-KEY%28%22cutaneous+healing%22%29%29%29+AND+%28%28TITLE-ABS-KEY%28%22skin%22%29+OR+TITLE-ABS-KEY%28%22dermis%22%29+OR+TITLE-ABS-KEY+%28%22epidermis%22%29+OR+TITLE-ABS-KEY%28%22subcutaneous+tissue%22%29+OR+TITLE-ABS-KEY%28%22hypodermis%22%29+OR+TITLE-ABS-KEY%28%22granulation+tissue%22%29+OR+TITLE-ABS-KEY%28%22keratinocytes%22%29+OR+TITLE-ABS-KEY%28%22fibroblasts%22%29+OR+TITLE-ABS-KEY+%28%22integumentary+system%22%29+OR+TITLE-ABS-KEY%28%22skin+injuries%22%29+OR+TITLE-ABS-KEY%28%22skin+fibrosis%22%29+OR+TITLE-ABS-KEY%28%22skin+scars%22%29%29%29&relpos=236&citeCnt=11&searchTerm=) \| \| [Analysis of healing effect of alginate sulfate hydrogel dressing containing antimicrobial peptide on wound infection caused by methicillin-resistant Staphylococcus aureus](https://www-scopus.ez35.periodicos.capes.gov.br/record/display.uri?eid=2-s2.0-84942155851&origin=resultslist&sort=plf-f&src=s&nlo=&nlr=&nls=&mltAll=t&sid=8f682cda9d1e11ee420f15ef65811fea&sot=comb&sdt=cl&cluster=scoexactkeywords%2c%22Animal+Experiment%22%2ct%2bscolang%2c%22English%22%2ct&sl=1001&s=%28%28TITLE-ABS-KEY%28%22peptides%22%29+OR+TITLE-ABS-KEY%28%22antioxidant+peptides%22%29+OR+TITLE-ABS-KEY+%28%22antimicrobial+peptides%22%29+OR+TITLE-ABS-KEY%28%22angiogenic+peptides%22%29+OR+TITLE-ABS-KEY+%28%22animal+peptides%22%29+OR+TITLE-ABS-KEY%28%22natural+peptides%22%29+OR+TITLE-ABS-KEY%28%22bioactive+peptides%22%29+OR+TITLE-ABS-KEY%28%22biological+peptides%22%29+OR+TITLE-ABS-KEY%28%22isolated+peptides%22%29+OR+TITLE-ABS-KEY%28%22extracted+peptides%22%29%29%29+AND+%28%28TITLE-ABS-KEY%28%22wound+healing%22%29+OR+TITLE-ABS-KEY%28%22regeneration%22%29+OR+TITLE-ABS-KEY%28%22skin+repair%22%29+OR+TITLE-ABS-KEY%28%22cutaneous+repair%22%29+OR+TITLE-ABS-KEY%28%22skin+healing%22%29+OR+TITLE-ABS-KEY%28%22cutaneous+healing%22%29%29%29+AND+%28%28TITLE-ABS-KEY%28%22skin%22%29+OR+TITLE-ABS-KEY%28%22dermis%22%29+OR+TITLE-ABS-KEY+%28%22epidermis%22%29+OR+TITLE-ABS-KEY%28%22subcutaneous+tissue%22%29+OR+TITLE-ABS-KEY%28%22hypodermis%22%29+OR+TITLE-ABS-KEY%28%22granulation+tissue%22%29+OR+TITLE-ABS-KEY%28%22keratinocytes%22%29+OR+TITLE-ABS-KEY%28%22fibroblasts%22%29+OR+TITLE-ABS-KEY+%28%22integumentary+system%22%29+OR+TITLE-ABS-KEY%28%22skin+injuries%22%29+OR+TITLE-ABS-KEY%28%22skin+fibrosis%22%29+OR+TITLE-ABS-KEY%28%22skin+scars%22%29%29%29&relpos=232&citeCnt=12&searchTerm=) \| \| [Angiogenic Heparin-Mimetic Peptide Nanofiber Gel Improves Regenerative Healing of Acute Wounds](https://www-scopus.ez35.periodicos.capes.gov.br/record/display.uri?eid=2-s2.0-85022207158&origin=resultslist&sort=plf-f&src=s&nlo=&nlr=&nls=&mltAll=t&sid=8f682cda9d1e11ee420f15ef65811fea&sot=comb&sdt=cl&cluster=scoexactkeywords%2c%22Animal+Experiment%22%2ct%2bscolang%2c%22English%22%2ct&sl=1001&s=%28%28TITLE-ABS-KEY%28%22peptides%22%29+OR+TITLE-ABS-KEY%28%22antioxidant+peptides%22%29+OR+TITLE-ABS-KEY+%28%22antimicrobial+peptides%22%29+OR+TITLE-ABS-KEY%28%22angiogenic+peptides%22%29+OR+TITLE-ABS-KEY+%28%22animal+peptides%22%29+OR+TITLE-ABS-KEY%28%22natural+peptides%22%29+OR+TITLE-ABS-KEY%28%22bioactive+peptides%22%29+OR+TITLE-ABS-KEY%28%22biological+peptides%22%29+OR+TITLE-ABS-KEY%28%22isolated+peptides%22%29+OR+TITLE-ABS-KEY%28%22extracted+peptides%22%29%29%29+AND+%28%28TITLE-ABS-KEY%28%22wound+healing%22%29+OR+TITLE-ABS-KEY%28%22regeneration%22%29+OR+TITLE-ABS-KEY%28%22skin+repair%22%29+OR+TITLE-ABS-KEY%28%22cutaneous+repair%22%29+OR+TITLE-ABS-KEY%28%22skin+healing%22%29+OR+TITLE-ABS-KEY%28%22cutaneous+healing%22%29%29%29+AND+%28%28TITLE-ABS-KEY%28%22skin%22%29+OR+TITLE-ABS-KEY%28%22dermis%22%29+OR+TITLE-ABS-KEY+%28%22epidermis%22%29+OR+TITLE-ABS-KEY%28%22subcutaneous+tissue%22%29+OR+TITLE-ABS-KEY%28%22hypodermis%22%29+OR+TITLE-ABS-KEY%28%22granulation+tissue%22%29+OR+TITLE-ABS-KEY%28%22keratinocytes%22%29+OR+TITLE-ABS-KEY%28%22fibroblasts%22%29+OR+TITLE-ABS-KEY+%28%22integumentary+system%22%29+OR+TITLE-ABS-KEY%28%22skin+injuries%22%29+OR+TITLE-ABS-KEY%28%22skin+fibrosis%22%29+OR+TITLE-ABS-KEY%28%22skin+scars%22%29%29%29&relpos=128&citeCnt=5&searchTerm=) \| \| [Angiogenic Peptide Nanofibers Improve Wound Healing in STZ-Induced Diabetic Rats](https://www-scopus.ez35.periodicos.capes.gov.br/record/display.uri?eid=2-s2.0-85006216000&origin=resultslist&sort=plf-f&src=s&nlo=&nlr=&nls=&mltAll=t&sid=8f682cda9d1e11ee420f15ef65811fea&sot=comb&sdt=cl&cluster=scoexactkeywords%2c%22Animal+Experiment%22%2ct%2bscolang%2c%22English%22%2ct&sl=1001&s=%28%28TITLE-ABS-KEY%28%22peptides%22%29+OR+TITLE-ABS-KEY%28%22antioxidant+peptides%22%29+OR+TITLE-ABS-KEY+%28%22antimicrobial+peptides%22%29+OR+TITLE-ABS-KEY%28%22angiogenic+peptides%22%29+OR+TITLE-ABS-KEY+%28%22animal+peptides%22%29+OR+TITLE-ABS-KEY%28%22natural+peptides%22%29+OR+TITLE-ABS-KEY%28%22bioactive+peptides%22%29+OR+TITLE-ABS-KEY%28%22biological+peptides%22%29+OR+TITLE-ABS-KEY%28%22isolated+peptides%22%29+OR+TITLE-ABS-KEY%28%22extracted+peptides%22%29%29%29+AND+%28%28TITLE-ABS-KEY%28%22wound+healing%22%29+OR+TITLE-ABS-KEY%28%22regeneration%22%29+OR+TITLE-ABS-KEY%28%22skin+repair%22%29+OR+TITLE-ABS-KEY%28%22cutaneous+repair%22%29+OR+TITLE-ABS-KEY%28%22skin+healing%22%29+OR+TITLE-ABS-KEY%28%22cutaneous+healing%22%29%29%29+AND+%28%28TITLE-ABS-KEY%28%22skin%22%29+OR+TITLE-ABS-KEY%28%22dermis%22%29+OR+TITLE-ABS-KEY+%28%22epidermis%22%29+OR+TITLE-ABS-KEY%28%22subcutaneous+tissue%22%29+OR+TITLE-ABS-KEY%28%22hypodermis%22%29+OR+TITLE-ABS-KEY%28%22granulation+tissue%22%29+OR+TITLE-ABS-KEY%28%22keratinocytes%22%29+OR+TITLE-ABS-KEY%28%22fibroblasts%22%29+OR+TITLE-ABS-KEY+%28%22integumentary+system%22%29+OR+TITLE-ABS-KEY%28%22skin+injuries%22%29+OR+TITLE-ABS-KEY%28%22skin+fibrosis%22%29+OR+TITLE-ABS-KEY%28%22skin+scars%22%29%29%29&relpos=181&citeCnt=7&searchTerm=) \| \| [Antimicrobial peptide-gold nanoscale therapeutic formulation with high skin regenerative potential](https://www-scopus.ez35.periodicos.capes.gov.br/record/display.uri?eid=2-s2.0-85025167046&origin=resultslist&sort=plf-f&src=s&nlo=&nlr=&nls=&mltAll=t&sid=8f682cda9d1e11ee420f15ef65811fea&sot=comb&sdt=cl&cluster=scoexactkeywords%2c%22Animal+Experiment%22%2ct%2bscolang%2c%22English%22%2ct&sl=1001&s=%28%28TITLE-ABS-KEY%28%22peptides%22%29+OR+TITLE-ABS-KEY%28%22antioxidant+peptides%22%29+OR+TITLE-ABS-KEY+%28%22antimicrobial+peptides%22%29+OR+TITLE-ABS-KEY%28%22angiogenic+peptides%22%29+OR+TITLE-ABS-KEY+%28%22animal+peptides%22%29+OR+TITLE-ABS-KEY%28%22natural+peptides%22%29+OR+TITLE-ABS-KEY%28%22bioactive+peptides%22%29+OR+TITLE-ABS-KEY%28%22biological+peptides%22%29+OR+TITLE-ABS-KEY%28%22isolated+peptides%22%29+OR+TITLE-ABS-KEY%28%22extracted+peptides%22%29%29%29+AND+%28%28TITLE-ABS-KEY%28%22wound+healing%22%29+OR+TITLE-ABS-KEY%28%22regeneration%22%29+OR+TITLE-ABS-KEY%28%22skin+repair%22%29+OR+TITLE-ABS-KEY%28%22cutaneous+repair%22%29+OR+TITLE-ABS-KEY%28%22skin+healing%22%29+OR+TITLE-ABS-KEY%28%22cutaneous+healing%22%29%29%29+AND+%28%28TITLE-ABS-KEY%28%22skin%22%29+OR+TITLE-ABS-KEY%28%22dermis%22%29+OR+TITLE-ABS-KEY+%28%22epidermis%22%29+OR+TITLE-ABS-KEY%28%22subcutaneous+tissue%22%29+OR+TITLE-ABS-KEY%28%22hypodermis%22%29+OR+TITLE-ABS-KEY%28%22granulation+tissue%22%29+OR+TITLE-ABS-KEY%28%22keratinocytes%22%29+OR+TITLE-ABS-KEY%28%22fibroblasts%22%29+OR+TITLE-ABS-KEY+%28%22integumentary+system%22%29+OR+TITLE-ABS-KEY%28%22skin+injuries%22%29+OR+TITLE-ABS-KEY%28%22skin+fibrosis%22%29+OR+TITLE-ABS-KEY%28%22skin+scars%22%29%29%29&relpos=114&citeCnt=8&searchTerm=) \| \| [Assessment of antimicrobial and wound healing effects of Brevinin-2Ta against the bacterium Klebsiella pneumoniae in dermally-wounded rats](https://www-scopus.ez35.periodicos.capes.gov.br/record/display.uri?eid=2-s2.0-85038432218&origin=resultslist&sort=plf-f&src=s&nlo=&nlr=&nls=&mltAll=t&sid=8f682cda9d1e11ee420f15ef65811fea&sot=comb&sdt=cl&cluster=scoexactkeywords%2c%22Animal+Experiment%22%2ct%2bscolang%2c%22English%22%2ct&sl=1001&s=%28%28TITLE-ABS-KEY%28%22peptides%22%29+OR+TITLE-ABS-KEY%28%22antioxidant+peptides%22%29+OR+TITLE-ABS-KEY+%28%22antimicrobial+peptides%22%29+OR+TITLE-ABS-KEY%28%22angiogenic+peptides%22%29+OR+TITLE-ABS-KEY+%28%22animal+peptides%22%29+OR+TITLE-ABS-KEY%28%22natural+peptides%22%29+OR+TITLE-ABS-KEY%28%22bioactive+peptides%22%29+OR+TITLE-ABS-KEY%28%22biological+peptides%22%29+OR+TITLE-ABS-KEY%28%22isolated+peptides%22%29+OR+TITLE-ABS-KEY%28%22extracted+peptides%22%29%29%29+AND+%28%28TITLE-ABS-KEY%28%22wound+healing%22%29+OR+TITLE-ABS-KEY%28%22regeneration%22%29+OR+TITLE-ABS-KEY%28%22skin+repair%22%29+OR+TITLE-ABS-KEY%28%22cutaneous+repair%22%29+OR+TITLE-ABS-KEY%28%22skin+healing%22%29+OR+TITLE-ABS-KEY%28%22cutaneous+healing%22%29%29%29+AND+%28%28TITLE-ABS-KEY%28%22skin%22%29+OR+TITLE-ABS-KEY%28%22dermis%22%29+OR+TITLE-ABS-KEY+%28%22epidermis%22%29+OR+TITLE-ABS-KEY%28%22subcutaneous+tissue%22%29+OR+TITLE-ABS-KEY%28%22hypodermis%22%29+OR+TITLE-ABS-KEY%28%22granulation+tissue%22%29+OR+TITLE-ABS-KEY%28%22keratinocytes%22%29+OR+TITLE-ABS-KEY%28%22fibroblasts%22%29+OR+TITLE-ABS-KEY+%28%22integumentary+system%22%29+OR+TITLE-ABS-KEY%28%22skin+injuries%22%29+OR+TITLE-ABS-KEY%28%22skin+fibrosis%22%29+OR+TITLE-ABS-KEY%28%22skin+scars%22%29%29%29&relpos=154&citeCnt=3&searchTerm=) \| \| [Atrial natriuretic peptide accelerates human endothelial progenitor cell–stimulated cutaneous wound healing and angiogenesis](https://www2.scopus.com/record/display.uri?eid=2-s2.0-85050730666&origin=resultslist&sort=plf-f&src=s&nlo=&nlr=&nls=&mltAll=t&sid=4d2dea855575f6fbdb403e5ce008a978&sot=comb&sdt=cl&cluster=scoexactkeywords%2c%22Animal+Experiment%22%2ct%2bscolang%2c%22English%22%2ct&sl=1001&s=%28%28TITLE-ABS-KEY%28%22peptides%22%29+OR+TITLE-ABS-KEY%28%22antioxidant+peptides%22%29+OR+TITLE-ABS-KEY+%28%22antimicrobial+peptides%22%29+OR+TITLE-ABS-KEY%28%22angiogenic+peptides%22%29+OR+TITLE-ABS-KEY+%28%22animal+peptides%22%29+OR+TITLE-ABS-KEY%28%22natural+peptides%22%29+OR+TITLE-ABS-KEY%28%22bioactive+peptides%22%29+OR+TITLE-ABS-KEY%28%22biological+peptides%22%29+OR+TITLE-ABS-KEY%28%22isolated+peptides%22%29+OR+TITLE-ABS-KEY%28%22extracted+peptides%22%29%29%29+AND+%28%28TITLE-ABS-KEY%28%22wound+healing%22%29+OR+TITLE-ABS-KEY%28%22regeneration%22%29+OR+TITLE-ABS-KEY%28%22skin+repair%22%29+OR+TITLE-ABS-KEY%28%22cutaneous+repair%22%29+OR+TITLE-ABS-KEY%28%22skin+healing%22%29+OR+TITLE-ABS-KEY%28%22cutaneous+healing%22%29%29%29+AND+%28%28TITLE-ABS-KEY%28%22skin%22%29+OR+TITLE-ABS-KEY%28%22dermis%22%29+OR+TITLE-ABS-KEY+%28%22epidermis%22%29+OR+TITLE-ABS-KEY%28%22subcutaneous+tissue%22%29+OR+TITLE-ABS-KEY%28%22hypodermis%22%29+OR+TITLE-ABS-KEY%28%22granulation+tissue%22%29+OR+TITLE-ABS-KEY%28%22keratinocytes%22%29+OR+TITLE-ABS-KEY%28%22fibroblasts%22%29+OR+TITLE-ABS-KEY+%28%22integumentary+system%22%29+OR+TITLE-ABS-KEY%28%22skin+injuries%22%29+OR+TITLE-ABS-KEY%28%22skin+fibrosis%22%29+OR+TITLE-ABS-KEY%28%22skin+scars%22%29%29%29&relpos=77&citeCnt=2&searchTerm=) \| \| [Bioactive peptide amphiphile nanofiber gels enhance burn wound healing](https://www2.scopus.com/record/display.uri?eid=2-s2.0-85064736115&origin=resultslist&sort=plf-f&src=s&nlo=&nlr=&nls=&mltAll=t&sid=4d2dea855575f6fbdb403e5ce008a978&sot=comb&sdt=cl&cluster=scoexactkeywords%2c%22Animal+Experiment%22%2ct%2bscolang%2c%22English%22%2ct&sl=1001&s=%28%28TITLE-ABS-KEY%28%22peptides%22%29+OR+TITLE-ABS-KEY%28%22antioxidant+peptides%22%29+OR+TITLE-ABS-KEY+%28%22antimicrobial+peptides%22%29+OR+TITLE-ABS-KEY%28%22angiogenic+peptides%22%29+OR+TITLE-ABS-KEY+%28%22animal+peptides%22%29+OR+TITLE-ABS-KEY%28%22natural+peptides%22%29+OR+TITLE-ABS-KEY%28%22bioactive+peptides%22%29+OR+TITLE-ABS-KEY%28%22biological+peptides%22%29+OR+TITLE-ABS-KEY%28%22isolated+peptides%22%29+OR+TITLE-ABS-KEY%28%22extracted+peptides%22%29%29%29+AND+%28%28TITLE-ABS-KEY%28%22wound+healing%22%29+OR+TITLE-ABS-KEY%28%22regeneration%22%29+OR+TITLE-ABS-KEY%28%22skin+repair%22%29+OR+TITLE-ABS-KEY%28%22cutaneous+repair%22%29+OR+TITLE-ABS-KEY%28%22skin+healing%22%29+OR+TITLE-ABS-KEY%28%22cutaneous+healing%22%29%29%29+AND+%28%28TITLE-ABS-KEY%28%22skin%22%29+OR+TITLE-ABS-KEY%28%22dermis%22%29+OR+TITLE-ABS-KEY+%28%22epidermis%22%29+OR+TITLE-ABS-KEY%28%22subcutaneous+tissue%22%29+OR+TITLE-ABS-KEY%28%22hypodermis%22%29+OR+TITLE-ABS-KEY%28%22granulation+tissue%22%29+OR+TITLE-ABS-KEY%28%22keratinocytes%22%29+OR+TITLE-ABS-KEY%28%22fibroblasts%22%29+OR+TITLE-ABS-KEY+%28%22integumentary+system%22%29+OR+TITLE-ABS-KEY%28%22skin+injuries%22%29+OR+TITLE-ABS-KEY%28%22skin+fibrosis%22%29+OR+TITLE-ABS-KEY%28%22skin+scars%22%29%29%29&relpos=0&citeCnt=0&searchTerm=) \| \| Biofunctions of antimicrobial peptide-conjugated alginate/hyaluronic acid/collagen wound dressings promote wound healing of a mixed-bacteria-infected wound \| \| [Biomacromolecule immobilization: Grafting of fish-scale collagen peptides onto aminolyzed P(3HB-co-4HB) scaffolds as a potential wound dressing](https://www-scopus.ez35.periodicos.capes.gov.br/record/display.uri?eid=2-s2.0-84992088226&origin=resultslist&sort=plf-f&src=s&nlo=&nlr=&nls=&mltAll=t&sid=8f682cda9d1e11ee420f15ef65811fea&sot=comb&sdt=cl&cluster=scoexactkeywords%2c%22Animal+Experiment%22%2ct%2bscolang%2c%22English%22%2ct&sl=1001&s=%28%28TITLE-ABS-KEY%28%22peptides%22%29+OR+TITLE-ABS-KEY%28%22antioxidant+peptides%22%29+OR+TITLE-ABS-KEY+%28%22antimicrobial+peptides%22%29+OR+TITLE-ABS-KEY%28%22angiogenic+peptides%22%29+OR+TITLE-ABS-KEY+%28%22animal+peptides%22%29+OR+TITLE-ABS-KEY%28%22natural+peptides%22%29+OR+TITLE-ABS-KEY%28%22bioactive+peptides%22%29+OR+TITLE-ABS-KEY%28%22biological+peptides%22%29+OR+TITLE-ABS-KEY%28%22isolated+peptides%22%29+OR+TITLE-ABS-KEY%28%22extracted+peptides%22%29%29%29+AND+%28%28TITLE-ABS-KEY%28%22wound+healing%22%29+OR+TITLE-ABS-KEY%28%22regeneration%22%29+OR+TITLE-ABS-KEY%28%22skin+repair%22%29+OR+TITLE-ABS-KEY%28%22cutaneous+repair%22%29+OR+TITLE-ABS-KEY%28%22skin+healing%22%29+OR+TITLE-ABS-KEY%28%22cutaneous+healing%22%29%29%29+AND+%28%28TITLE-ABS-KEY%28%22skin%22%29+OR+TITLE-ABS-KEY%28%22dermis%22%29+OR+TITLE-ABS-KEY+%28%22epidermis%22%29+OR+TITLE-ABS-KEY%28%22subcutaneous+tissue%22%29+OR+TITLE-ABS-KEY%28%22hypodermis%22%29+OR+TITLE-ABS-KEY%28%22granulation+tissue%22%29+OR+TITLE-ABS-KEY%28%22keratinocytes%22%29+OR+TITLE-ABS-KEY%28%22fibroblasts%22%29+OR+TITLE-ABS-KEY+%28%22integumentary+system%22%29+OR+TITLE-ABS-KEY%28%22skin+injuries%22%29+OR+TITLE-ABS-KEY%28%22skin+fibrosis%22%29+OR+TITLE-ABS-KEY%28%22skin+scars%22%29%29%29&relpos=172&citeCnt=6&searchTerm=) \| \| [Biotinylated GHK peptide incorporated collagenous matrix: A novel biomaterial for dermal wound healing in rats](https://www-scopus.ez35.periodicos.capes.gov.br/record/display.uri?eid=2-s2.0-18244403133&origin=resultslist&sort=plf-f&src=s&nlo=&nlr=&nls=&mltAll=t&sid=8f682cda9d1e11ee420f15ef65811fea&sot=comb&sdt=cl&cluster=scoexactkeywords%2c%22Animal+Experiment%22%2ct%2bscolang%2c%22English%22%2ct&sl=1001&s=%28%28TITLE-ABS-KEY%28%22peptides%22%29+OR+TITLE-ABS-KEY%28%22antioxidant+peptides%22%29+OR+TITLE-ABS-KEY+%28%22antimicrobial+peptides%22%29+OR+TITLE-ABS-KEY%28%22angiogenic+peptides%22%29+OR+TITLE-ABS-KEY+%28%22animal+peptides%22%29+OR+TITLE-ABS-KEY%28%22natural+peptides%22%29+OR+TITLE-ABS-KEY%28%22bioactive+peptides%22%29+OR+TITLE-ABS-KEY%28%22biological+peptides%22%29+OR+TITLE-ABS-KEY%28%22isolated+peptides%22%29+OR+TITLE-ABS-KEY%28%22extracted+peptides%22%29%29%29+AND+%28%28TITLE-ABS-KEY%28%22wound+healing%22%29+OR+TITLE-ABS-KEY%28%22regeneration%22%29+OR+TITLE-ABS-KEY%28%22skin+repair%22%29+OR+TITLE-ABS-KEY%28%22cutaneous+repair%22%29+OR+TITLE-ABS-KEY%28%22skin+healing%22%29+OR+TITLE-ABS-KEY%28%22cutaneous+healing%22%29%29%29+AND+%28%28TITLE-ABS-KEY%28%22skin%22%29+OR+TITLE-ABS-KEY%28%22dermis%22%29+OR+TITLE-ABS-KEY+%28%22epidermis%22%29+OR+TITLE-ABS-KEY%28%22subcutaneous+tissue%22%29+OR+TITLE-ABS-KEY%28%22hypodermis%22%29+OR+TITLE-ABS-KEY%28%22granulation+tissue%22%29+OR+TITLE-ABS-KEY%28%22keratinocytes%22%29+OR+TITLE-ABS-KEY%28%22fibroblasts%22%29+OR+TITLE-ABS-KEY+%28%22integumentary+system%22%29+OR+TITLE-ABS-KEY%28%22skin+injuries%22%29+OR+TITLE-ABS-KEY%28%22skin+fibrosis%22%29+OR+TITLE-ABS-KEY%28%22skin+scars%22%29%29%29&relpos=647&citeCnt=34&searchTerm=) \| \| [Body protective compound-157 enhances alkali-burn wound healing in vivo and promotes proliferation, migration, and angiogenesis in vitro](https://www-scopus.ez35.periodicos.capes.gov.br/record/display.uri?eid=2-s2.0-84929191247&origin=resultslist&sort=plf-f&src=s&nlo=&nlr=&nls=&mltAll=t&sid=8f682cda9d1e11ee420f15ef65811fea&sot=comb&sdt=cl&cluster=scoexactkeywords%2c%22Animal+Experiment%22%2ct%2bscolang%2c%22English%22%2ct&sl=1001&s=%28%28TITLE-ABS-KEY%28%22peptides%22%29+OR+TITLE-ABS-KEY%28%22antioxidant+peptides%22%29+OR+TITLE-ABS-KEY+%28%22antimicrobial+peptides%22%29+OR+TITLE-ABS-KEY%28%22angiogenic+peptides%22%29+OR+TITLE-ABS-KEY+%28%22animal+peptides%22%29+OR+TITLE-ABS-KEY%28%22natural+peptides%22%29+OR+TITLE-ABS-KEY%28%22bioactive+peptides%22%29+OR+TITLE-ABS-KEY%28%22biological+peptides%22%29+OR+TITLE-ABS-KEY%28%22isolated+peptides%22%29+OR+TITLE-ABS-KEY%28%22extracted+peptides%22%29%29%29+AND+%28%28TITLE-ABS-KEY%28%22wound+healing%22%29+OR+TITLE-ABS-KEY%28%22regeneration%22%29+OR+TITLE-ABS-KEY%28%22skin+repair%22%29+OR+TITLE-ABS-KEY%28%22cutaneous+repair%22%29+OR+TITLE-ABS-KEY%28%22skin+healing%22%29+OR+TITLE-ABS-KEY%28%22cutaneous+healing%22%29%29%29+AND+%28%28TITLE-ABS-KEY%28%22skin%22%29+OR+TITLE-ABS-KEY%28%22dermis%22%29+OR+TITLE-ABS-KEY+%28%22epidermis%22%29+OR+TITLE-ABS-KEY%28%22subcutaneous+tissue%22%29+OR+TITLE-ABS-KEY%28%22hypodermis%22%29+OR+TITLE-ABS-KEY%28%22granulation+tissue%22%29+OR+TITLE-ABS-KEY%28%22keratinocytes%22%29+OR+TITLE-ABS-KEY%28%22fibroblasts%22%29+OR+TITLE-ABS-KEY+%28%22integumentary+system%22%29+OR+TITLE-ABS-KEY%28%22skin+injuries%22%29+OR+TITLE-ABS-KEY%28%22skin+fibrosis%22%29+OR+TITLE-ABS-KEY%28%22skin+scars%22%29%29%29&relpos=252&citeCnt=30&searchTerm=) \| \| [Carboxymethyl chitosan nanoparticles loaded with bioactive peptide OH-CATH30 benefit nonscar wound healing](https://www-scopus.ez35.periodicos.capes.gov.br/record/display.uri?eid=2-s2.0-85054780053&origin=resultslist&sort=plf-f&src=s&nlo=&nlr=&nls=&mltAll=t&sid=8f682cda9d1e11ee420f15ef65811fea&sot=comb&sdt=cl&cluster=scoexactkeywords%2c%22Animal+Experiment%22%2ct%2bscolang%2c%22English%22%2ct&sl=1001&s=%28%28TITLE-ABS-KEY%28%22peptides%22%29+OR+TITLE-ABS-KEY%28%22antioxidant+peptides%22%29+OR+TITLE-ABS-KEY+%28%22antimicrobial+peptides%22%29+OR+TITLE-ABS-KEY%28%22angiogenic+peptides%22%29+OR+TITLE-ABS-KEY+%28%22animal+peptides%22%29+OR+TITLE-ABS-KEY%28%22natural+peptides%22%29+OR+TITLE-ABS-KEY%28%22bioactive+peptides%22%29+OR+TITLE-ABS-KEY%28%22biological+peptides%22%29+OR+TITLE-ABS-KEY%28%22isolated+peptides%22%29+OR+TITLE-ABS-KEY%28%22extracted+peptides%22%29%29%29+AND+%28%28TITLE-ABS-KEY%28%22wound+healing%22%29+OR+TITLE-ABS-KEY%28%22regeneration%22%29+OR+TITLE-ABS-KEY%28%22skin+repair%22%29+OR+TITLE-ABS-KEY%28%22cutaneous+repair%22%29+OR+TITLE-ABS-KEY%28%22skin+healing%22%29+OR+TITLE-ABS-KEY%28%22cutaneous+healing%22%29%29%29+AND+%28%28TITLE-ABS-KEY%28%22skin%22%29+OR+TITLE-ABS-KEY%28%22dermis%22%29+OR+TITLE-ABS-KEY+%28%22epidermis%22%29+OR+TITLE-ABS-KEY%28%22subcutaneous+tissue%22%29+OR+TITLE-ABS-KEY%28%22hypodermis%22%29+OR+TITLE-ABS-KEY%28%22granulation+tissue%22%29+OR+TITLE-ABS-KEY%28%22keratinocytes%22%29+OR+TITLE-ABS-KEY%28%22fibroblasts%22%29+OR+TITLE-ABS-KEY+%28%22integumentary+system%22%29+OR+TITLE-ABS-KEY%28%22skin+injuries%22%29+OR+TITLE-ABS-KEY%28%22skin+fibrosis%22%29+OR+TITLE-ABS-KEY%28%22skin+scars%22%29%29%29&relpos=91&citeCnt=3&searchTerm=) \| \| Chitosan hydrogel encapsulated with LL-37 peptide promotes deep tissue injury healing in a mouse model \| \| [Chitosan hydrogel in combination with marine peptides from tilapia for burns healing](https://www2.scopus.com/record/display.uri?eid=2-s2.0-85042193884&origin=resultslist&sort=plf-f&src=s&nlo=&nlr=&nls=&mltAll=t&sid=4d2dea855575f6fbdb403e5ce008a978&sot=comb&sdt=cl&cluster=scoexactkeywords%2c%22Animal+Experiment%22%2ct%2bscolang%2c%22English%22%2ct&sl=1001&s=%28%28TITLE-ABS-KEY%28%22peptides%22%29+OR+TITLE-ABS-KEY%28%22antioxidant+peptides%22%29+OR+TITLE-ABS-KEY+%28%22antimicrobial+peptides%22%29+OR+TITLE-ABS-KEY%28%22angiogenic+peptides%22%29+OR+TITLE-ABS-KEY+%28%22animal+peptides%22%29+OR+TITLE-ABS-KEY%28%22natural+peptides%22%29+OR+TITLE-ABS-KEY%28%22bioactive+peptides%22%29+OR+TITLE-ABS-KEY%28%22biological+peptides%22%29+OR+TITLE-ABS-KEY%28%22isolated+peptides%22%29+OR+TITLE-ABS-KEY%28%22extracted+peptides%22%29%29%29+AND+%28%28TITLE-ABS-KEY%28%22wound+healing%22%29+OR+TITLE-ABS-KEY%28%22regeneration%22%29+OR+TITLE-ABS-KEY%28%22skin+repair%22%29+OR+TITLE-ABS-KEY%28%22cutaneous+repair%22%29+OR+TITLE-ABS-KEY%28%22skin+healing%22%29+OR+TITLE-ABS-KEY%28%22cutaneous+healing%22%29%29%29+AND+%28%28TITLE-ABS-KEY%28%22skin%22%29+OR+TITLE-ABS-KEY%28%22dermis%22%29+OR+TITLE-ABS-KEY+%28%22epidermis%22%29+OR+TITLE-ABS-KEY%28%22subcutaneous+tissue%22%29+OR+TITLE-ABS-KEY%28%22hypodermis%22%29+OR+TITLE-ABS-KEY%28%22granulation+tissue%22%29+OR+TITLE-ABS-KEY%28%22keratinocytes%22%29+OR+TITLE-ABS-KEY%28%22fibroblasts%22%29+OR+TITLE-ABS-KEY+%28%22integumentary+system%22%29+OR+TITLE-ABS-KEY%28%22skin+injuries%22%29+OR+TITLE-ABS-KEY%28%22skin+fibrosis%22%29+OR+TITLE-ABS-KEY%28%22skin+scars%22%29%29%29&relpos=66&citeCnt=7&searchTerm=) \| \| Co-assembled supramolecular hydrogels of cell adhesive peptide and alginate for rapid hemostasis and efficacious wound healing \| \| [Connexin43 carboxyl-terminal peptides reduce scar progenitor and promote regenerative healing following skin wounding](https://www-scopus.ez35.periodicos.capes.gov.br/record/display.uri?eid=2-s2.0-65549168273&origin=resultslist&sort=plf-f&src=s&nlo=&nlr=&nls=&mltAll=t&sid=8f682cda9d1e11ee420f15ef65811fea&sot=comb&sdt=cl&cluster=scoexactkeywords%2c%22Animal+Experiment%22%2ct%2bscolang%2c%22English%22%2ct&sl=1001&s=%28%28TITLE-ABS-KEY%28%22peptides%22%29+OR+TITLE-ABS-KEY%28%22antioxidant+peptides%22%29+OR+TITLE-ABS-KEY+%28%22antimicrobial+peptides%22%29+OR+TITLE-ABS-KEY%28%22angiogenic+peptides%22%29+OR+TITLE-ABS-KEY+%28%22animal+peptides%22%29+OR+TITLE-ABS-KEY%28%22natural+peptides%22%29+OR+TITLE-ABS-KEY%28%22bioactive+peptides%22%29+OR+TITLE-ABS-KEY%28%22biological+peptides%22%29+OR+TITLE-ABS-KEY%28%22isolated+peptides%22%29+OR+TITLE-ABS-KEY%28%22extracted+peptides%22%29%29%29+AND+%28%28TITLE-ABS-KEY%28%22wound+healing%22%29+OR+TITLE-ABS-KEY%28%22regeneration%22%29+OR+TITLE-ABS-KEY%28%22skin+repair%22%29+OR+TITLE-ABS-KEY%28%22cutaneous+repair%22%29+OR+TITLE-ABS-KEY%28%22skin+healing%22%29+OR+TITLE-ABS-KEY%28%22cutaneous+healing%22%29%29%29+AND+%28%28TITLE-ABS-KEY%28%22skin%22%29+OR+TITLE-ABS-KEY%28%22dermis%22%29+OR+TITLE-ABS-KEY+%28%22epidermis%22%29+OR+TITLE-ABS-KEY%28%22subcutaneous+tissue%22%29+OR+TITLE-ABS-KEY%28%22hypodermis%22%29+OR+TITLE-ABS-KEY%28%22granulation+tissue%22%29+OR+TITLE-ABS-KEY%28%22keratinocytes%22%29+OR+TITLE-ABS-KEY%28%22fibroblasts%22%29+OR+TITLE-ABS-KEY+%28%22integumentary+system%22%29+OR+TITLE-ABS-KEY%28%22skin+injuries%22%29+OR+TITLE-ABS-KEY%28%22skin+fibrosis%22%29+OR+TITLE-ABS-KEY%28%22skin+scars%22%29%29%29&relpos=513&citeCnt=82&searchTerm=) \| \| [Design of antimicrobial peptides conjugated biodegradable citric acid derived hydrogels for wound healing](https://www-scopus.ez35.periodicos.capes.gov.br/record/display.uri?eid=2-s2.0-84945458974&origin=resultslist&sort=plf-f&src=s&nlo=&nlr=&nls=&mltAll=t&sid=8f682cda9d1e11ee420f15ef65811fea&sot=comb&sdt=cl&cluster=scoexactkeywords%2c%22Animal+Experiment%22%2ct%2bscolang%2c%22English%22%2ct&sl=1001&s=%28%28TITLE-ABS-KEY%28%22peptides%22%29+OR+TITLE-ABS-KEY%28%22antioxidant+peptides%22%29+OR+TITLE-ABS-KEY+%28%22antimicrobial+peptides%22%29+OR+TITLE-ABS-KEY%28%22angiogenic+peptides%22%29+OR+TITLE-ABS-KEY+%28%22animal+peptides%22%29+OR+TITLE-ABS-KEY%28%22natural+peptides%22%29+OR+TITLE-ABS-KEY%28%22bioactive+peptides%22%29+OR+TITLE-ABS-KEY%28%22biological+peptides%22%29+OR+TITLE-ABS-KEY%28%22isolated+peptides%22%29+OR+TITLE-ABS-KEY%28%22extracted+peptides%22%29%29%29+AND+%28%28TITLE-ABS-KEY%28%22wound+healing%22%29+OR+TITLE-ABS-KEY%28%22regeneration%22%29+OR+TITLE-ABS-KEY%28%22skin+repair%22%29+OR+TITLE-ABS-KEY%28%22cutaneous+repair%22%29+OR+TITLE-ABS-KEY%28%22skin+healing%22%29+OR+TITLE-ABS-KEY%28%22cutaneous+healing%22%29%29%29+AND+%28%28TITLE-ABS-KEY%28%22skin%22%29+OR+TITLE-ABS-KEY%28%22dermis%22%29+OR+TITLE-ABS-KEY+%28%22epidermis%22%29+OR+TITLE-ABS-KEY%28%22subcutaneous+tissue%22%29+OR+TITLE-ABS-KEY%28%22hypodermis%22%29+OR+TITLE-ABS-KEY%28%22granulation+tissue%22%29+OR+TITLE-ABS-KEY%28%22keratinocytes%22%29+OR+TITLE-ABS-KEY%28%22fibroblasts%22%29+OR+TITLE-ABS-KEY+%28%22integumentary+system%22%29+OR+TITLE-ABS-KEY%28%22skin+injuries%22%29+OR+TITLE-ABS-KEY%28%22skin+fibrosis%22%29+OR+TITLE-ABS-KEY%28%22skin+scars%22%29%29%29&relpos=217&citeCnt=18&searchTerm=) \| \| [Development of alginate wound dressings linked with hybrid peptides derived from laminin and elastin](https://www-scopus.ez35.periodicos.capes.gov.br/record/display.uri?eid=2-s2.0-0344825233&origin=resultslist&sort=plf-f&src=s&nlo=&nlr=&nls=&mltAll=t&sid=8f682cda9d1e11ee420f15ef65811fea&sot=comb&sdt=cl&cluster=scoexactkeywords%2c%22Animal+Experiment%22%2ct%2bscolang%2c%22English%22%2ct&sl=1001&s=%28%28TITLE-ABS-KEY%28%22peptides%22%29+OR+TITLE-ABS-KEY%28%22antioxidant+peptides%22%29+OR+TITLE-ABS-KEY+%28%22antimicrobial+peptides%22%29+OR+TITLE-ABS-KEY%28%22angiogenic+peptides%22%29+OR+TITLE-ABS-KEY+%28%22animal+peptides%22%29+OR+TITLE-ABS-KEY%28%22natural+peptides%22%29+OR+TITLE-ABS-KEY%28%22bioactive+peptides%22%29+OR+TITLE-ABS-KEY%28%22biological+peptides%22%29+OR+TITLE-ABS-KEY%28%22isolated+peptides%22%29+OR+TITLE-ABS-KEY%28%22extracted+peptides%22%29%29%29+AND+%28%28TITLE-ABS-KEY%28%22wound+healing%22%29+OR+TITLE-ABS-KEY%28%22regeneration%22%29+OR+TITLE-ABS-KEY%28%22skin+repair%22%29+OR+TITLE-ABS-KEY%28%22cutaneous+repair%22%29+OR+TITLE-ABS-KEY%28%22skin+healing%22%29+OR+TITLE-ABS-KEY%28%22cutaneous+healing%22%29%29%29+AND+%28%28TITLE-ABS-KEY%28%22skin%22%29+OR+TITLE-ABS-KEY%28%22dermis%22%29+OR+TITLE-ABS-KEY+%28%22epidermis%22%29+OR+TITLE-ABS-KEY%28%22subcutaneous+tissue%22%29+OR+TITLE-ABS-KEY%28%22hypodermis%22%29+OR+TITLE-ABS-KEY%28%22granulation+tissue%22%29+OR+TITLE-ABS-KEY%28%22keratinocytes%22%29+OR+TITLE-ABS-KEY%28%22fibroblasts%22%29+OR+TITLE-ABS-KEY+%28%22integumentary+system%22%29+OR+TITLE-ABS-KEY%28%22skin+injuries%22%29+OR+TITLE-ABS-KEY%28%22skin+fibrosis%22%29+OR+TITLE-ABS-KEY%28%22skin+scars%22%29%29%29&relpos=674&citeCnt=115&searchTerm=) \| \| [Development of angiotensin (1-7) as an agent to accelerate dermal repair](https://www-scopus.ez35.periodicos.capes.gov.br/record/display.uri?eid=2-s2.0-0034899508&origin=resultslist&sort=plf-f&src=s&nlo=&nlr=&nls=&mltAll=t&sid=8f682cda9d1e11ee420f15ef65811fea&sot=comb&sdt=cl&cluster=scoexactkeywords%2c%22Animal+Experiment%22%2ct%2bscolang%2c%22English%22%2ct&sl=1001&s=%28%28TITLE-ABS-KEY%28%22peptides%22%29+OR+TITLE-ABS-KEY%28%22antioxidant+peptides%22%29+OR+TITLE-ABS-KEY+%28%22antimicrobial+peptides%22%29+OR+TITLE-ABS-KEY%28%22angiogenic+peptides%22%29+OR+TITLE-ABS-KEY+%28%22animal+peptides%22%29+OR+TITLE-ABS-KEY%28%22natural+peptides%22%29+OR+TITLE-ABS-KEY%28%22bioactive+peptides%22%29+OR+TITLE-ABS-KEY%28%22biological+peptides%22%29+OR+TITLE-ABS-KEY%28%22isolated+peptides%22%29+OR+TITLE-ABS-KEY%28%22extracted+peptides%22%29%29%29+AND+%28%28TITLE-ABS-KEY%28%22wound+healing%22%29+OR+TITLE-ABS-KEY%28%22regeneration%22%29+OR+TITLE-ABS-KEY%28%22skin+repair%22%29+OR+TITLE-ABS-KEY%28%22cutaneous+repair%22%29+OR+TITLE-ABS-KEY%28%22skin+healing%22%29+OR+TITLE-ABS-KEY%28%22cutaneous+healing%22%29%29%29+AND+%28%28TITLE-ABS-KEY%28%22skin%22%29+OR+TITLE-ABS-KEY%28%22dermis%22%29+OR+TITLE-ABS-KEY+%28%22epidermis%22%29+OR+TITLE-ABS-KEY%28%22subcutaneous+tissue%22%29+OR+TITLE-ABS-KEY%28%22hypodermis%22%29+OR+TITLE-ABS-KEY%28%22granulation+tissue%22%29+OR+TITLE-ABS-KEY%28%22keratinocytes%22%29+OR+TITLE-ABS-KEY%28%22fibroblasts%22%29+OR+TITLE-ABS-KEY+%28%22integumentary+system%22%29+OR+TITLE-ABS-KEY%28%22skin+injuries%22%29+OR+TITLE-ABS-KEY%28%22skin+fibrosis%22%29+OR+TITLE-ABS-KEY%28%22skin+scars%22%29%29%29&relpos=723&citeCnt=43&searchTerm=) \| \| [Diabetic wound regeneration using heparin-mimetic peptide amphiphile gel in db/db mice](https://www-scopus.ez35.periodicos.capes.gov.br/record/display.uri?eid=2-s2.0-85021718141&origin=resultslist&sort=plf-f&src=s&nlo=&nlr=&nls=&mltAll=t&sid=8f682cda9d1e11ee420f15ef65811fea&sot=comb&sdt=cl&cluster=scoexactkeywords%2c%22Animal+Experiment%22%2ct%2bscolang%2c%22English%22%2ct&sl=1001&s=%28%28TITLE-ABS-KEY%28%22peptides%22%29+OR+TITLE-ABS-KEY%28%22antioxidant+peptides%22%29+OR+TITLE-ABS-KEY+%28%22antimicrobial+peptides%22%29+OR+TITLE-ABS-KEY%28%22angiogenic+peptides%22%29+OR+TITLE-ABS-KEY+%28%22animal+peptides%22%29+OR+TITLE-ABS-KEY%28%22natural+peptides%22%29+OR+TITLE-ABS-KEY%28%22bioactive+peptides%22%29+OR+TITLE-ABS-KEY%28%22biological+peptides%22%29+OR+TITLE-ABS-KEY%28%22isolated+peptides%22%29+OR+TITLE-ABS-KEY%28%22extracted+peptides%22%29%29%29+AND+%28%28TITLE-ABS-KEY%28%22wound+healing%22%29+OR+TITLE-ABS-KEY%28%22regeneration%22%29+OR+TITLE-ABS-KEY%28%22skin+repair%22%29+OR+TITLE-ABS-KEY%28%22cutaneous+repair%22%29+OR+TITLE-ABS-KEY%28%22skin+healing%22%29+OR+TITLE-ABS-KEY%28%22cutaneous+healing%22%29%29%29+AND+%28%28TITLE-ABS-KEY%28%22skin%22%29+OR+TITLE-ABS-KEY%28%22dermis%22%29+OR+TITLE-ABS-KEY+%28%22epidermis%22%29+OR+TITLE-ABS-KEY%28%22subcutaneous+tissue%22%29+OR+TITLE-ABS-KEY%28%22hypodermis%22%29+OR+TITLE-ABS-KEY%28%22granulation+tissue%22%29+OR+TITLE-ABS-KEY%28%22keratinocytes%22%29+OR+TITLE-ABS-KEY%28%22fibroblasts%22%29+OR+TITLE-ABS-KEY+%28%22integumentary+system%22%29+OR+TITLE-ABS-KEY%28%22skin+injuries%22%29+OR+TITLE-ABS-KEY%28%22skin+fibrosis%22%29+OR+TITLE-ABS-KEY%28%22skin+scars%22%29%29%29&relpos=132&citeCnt=5&searchTerm=) \| \| [Diabetic wound regeneration using peptide-modified hydrogels to target re-epithelialization](https://www-scopus.ez35.periodicos.capes.gov.br/record/display.uri?eid=2-s2.0-84989829732&origin=resultslist&sort=plf-f&src=s&nlo=&nlr=&nls=&mltAll=t&sid=8f682cda9d1e11ee420f15ef65811fea&sot=comb&sdt=cl&cluster=scoexactkeywords%2c%22Animal+Experiment%22%2ct%2bscolang%2c%22English%22%2ct&sl=1001&s=%28%28TITLE-ABS-KEY%28%22peptides%22%29+OR+TITLE-ABS-KEY%28%22antioxidant+peptides%22%29+OR+TITLE-ABS-KEY+%28%22antimicrobial+peptides%22%29+OR+TITLE-ABS-KEY%28%22angiogenic+peptides%22%29+OR+TITLE-ABS-KEY+%28%22animal+peptides%22%29+OR+TITLE-ABS-KEY%28%22natural+peptides%22%29+OR+TITLE-ABS-KEY%28%22bioactive+peptides%22%29+OR+TITLE-ABS-KEY%28%22biological+peptides%22%29+OR+TITLE-ABS-KEY%28%22isolated+peptides%22%29+OR+TITLE-ABS-KEY%28%22extracted+peptides%22%29%29%29+AND+%28%28TITLE-ABS-KEY%28%22wound+healing%22%29+OR+TITLE-ABS-KEY%28%22regeneration%22%29+OR+TITLE-ABS-KEY%28%22skin+repair%22%29+OR+TITLE-ABS-KEY%28%22cutaneous+repair%22%29+OR+TITLE-ABS-KEY%28%22skin+healing%22%29+OR+TITLE-ABS-KEY%28%22cutaneous+healing%22%29%29%29+AND+%28%28TITLE-ABS-KEY%28%22skin%22%29+OR+TITLE-ABS-KEY%28%22dermis%22%29+OR+TITLE-ABS-KEY+%28%22epidermis%22%29+OR+TITLE-ABS-KEY%28%22subcutaneous+tissue%22%29+OR+TITLE-ABS-KEY%28%22hypodermis%22%29+OR+TITLE-ABS-KEY%28%22granulation+tissue%22%29+OR+TITLE-ABS-KEY%28%22keratinocytes%22%29+OR+TITLE-ABS-KEY%28%22fibroblasts%22%29+OR+TITLE-ABS-KEY+%28%22integumentary+system%22%29+OR+TITLE-ABS-KEY%28%22skin+injuries%22%29+OR+TITLE-ABS-KEY%28%22skin+fibrosis%22%29+OR+TITLE-ABS-KEY%28%22skin+scars%22%29%29%29&relpos=173&citeCnt=25&searchTerm=) \| \| [Effect of fibrin-binding synthetic oligopeptide on the healing of full-thickness skin wounds in streptozotocin-induced diabetic rats](https://www-scopus.ez35.periodicos.capes.gov.br/record/display.uri?eid=2-s2.0-84876736457&origin=resultslist&sort=plf-f&src=s&nlo=&nlr=&nls=&mltAll=t&sid=8f682cda9d1e11ee420f15ef65811fea&sot=comb&sdt=cl&cluster=scoexactkeywords%2c%22Animal+Experiment%22%2ct%2bscolang%2c%22English%22%2ct&sl=1001&s=%28%28TITLE-ABS-KEY%28%22peptides%22%29+OR+TITLE-ABS-KEY%28%22antioxidant+peptides%22%29+OR+TITLE-ABS-KEY+%28%22antimicrobial+peptides%22%29+OR+TITLE-ABS-KEY%28%22angiogenic+peptides%22%29+OR+TITLE-ABS-KEY+%28%22animal+peptides%22%29+OR+TITLE-ABS-KEY%28%22natural+peptides%22%29+OR+TITLE-ABS-KEY%28%22bioactive+peptides%22%29+OR+TITLE-ABS-KEY%28%22biological+peptides%22%29+OR+TITLE-ABS-KEY%28%22isolated+peptides%22%29+OR+TITLE-ABS-KEY%28%22extracted+peptides%22%29%29%29+AND+%28%28TITLE-ABS-KEY%28%22wound+healing%22%29+OR+TITLE-ABS-KEY%28%22regeneration%22%29+OR+TITLE-ABS-KEY%28%22skin+repair%22%29+OR+TITLE-ABS-KEY%28%22cutaneous+repair%22%29+OR+TITLE-ABS-KEY%28%22skin+healing%22%29+OR+TITLE-ABS-KEY%28%22cutaneous+healing%22%29%29%29+AND+%28%28TITLE-ABS-KEY%28%22skin%22%29+OR+TITLE-ABS-KEY%28%22dermis%22%29+OR+TITLE-ABS-KEY+%28%22epidermis%22%29+OR+TITLE-ABS-KEY%28%22subcutaneous+tissue%22%29+OR+TITLE-ABS-KEY%28%22hypodermis%22%29+OR+TITLE-ABS-KEY%28%22granulation+tissue%22%29+OR+TITLE-ABS-KEY%28%22keratinocytes%22%29+OR+TITLE-ABS-KEY%28%22fibroblasts%22%29+OR+TITLE-ABS-KEY+%28%22integumentary+system%22%29+OR+TITLE-ABS-KEY%28%22skin+injuries%22%29+OR+TITLE-ABS-KEY%28%22skin+fibrosis%22%29+OR+TITLE-ABS-KEY%28%22skin+scars%22%29%29%29&relpos=353&citeCnt=2&searchTerm=) \| \| [Effect of synthetic peptide thrombin receptor agonist encapsulated in microparticles based on lactic and glycolic acid copolymer on healing of experimental skin wounds in mice](https://www-scopus.ez35.periodicos.capes.gov.br/record/display.uri?eid=2-s2.0-15544391136&origin=resultslist&sort=plf-f&src=s&nlo=&nlr=&nls=&mltAll=t&sid=8f682cda9d1e11ee420f15ef65811fea&sot=comb&sdt=cl&cluster=scoexactkeywords%2c%22Animal+Experiment%22%2ct%2bscolang%2c%22English%22%2ct&sl=1001&s=%28%28TITLE-ABS-KEY%28%22peptides%22%29+OR+TITLE-ABS-KEY%28%22antioxidant+peptides%22%29+OR+TITLE-ABS-KEY+%28%22antimicrobial+peptides%22%29+OR+TITLE-ABS-KEY%28%22angiogenic+peptides%22%29+OR+TITLE-ABS-KEY+%28%22animal+peptides%22%29+OR+TITLE-ABS-KEY%28%22natural+peptides%22%29+OR+TITLE-ABS-KEY%28%22bioactive+peptides%22%29+OR+TITLE-ABS-KEY%28%22biological+peptides%22%29+OR+TITLE-ABS-KEY%28%22isolated+peptides%22%29+OR+TITLE-ABS-KEY%28%22extracted+peptides%22%29%29%29+AND+%28%28TITLE-ABS-KEY%28%22wound+healing%22%29+OR+TITLE-ABS-KEY%28%22regeneration%22%29+OR+TITLE-ABS-KEY%28%22skin+repair%22%29+OR+TITLE-ABS-KEY%28%22cutaneous+repair%22%29+OR+TITLE-ABS-KEY%28%22skin+healing%22%29+OR+TITLE-ABS-KEY%28%22cutaneous+healing%22%29%29%29+AND+%28%28TITLE-ABS-KEY%28%22skin%22%29+OR+TITLE-ABS-KEY%28%22dermis%22%29+OR+TITLE-ABS-KEY+%28%22epidermis%22%29+OR+TITLE-ABS-KEY%28%22subcutaneous+tissue%22%29+OR+TITLE-ABS-KEY%28%22hypodermis%22%29+OR+TITLE-ABS-KEY%28%22granulation+tissue%22%29+OR+TITLE-ABS-KEY%28%22keratinocytes%22%29+OR+TITLE-ABS-KEY%28%22fibroblasts%22%29+OR+TITLE-ABS-KEY+%28%22integumentary+system%22%29+OR+TITLE-ABS-KEY%28%22skin+injuries%22%29+OR+TITLE-ABS-KEY%28%22skin+fibrosis%22%29+OR+TITLE-ABS-KEY%28%22skin+scars%22%29%29%29&relpos=658&citeCnt=8&searchTerm=) \| \| [Electrospinning of In situ crosslinked recombinant human collagen peptide/chitosan nanofibers for wound healing](https://www2.scopus.com/record/display.uri?eid=2-s2.0-85050723945&origin=resultslist&sort=plf-f&src=s&nlo=&nlr=&nls=&mltAll=t&sid=4d2dea855575f6fbdb403e5ce008a978&sot=comb&sdt=cl&cluster=scoexactkeywords%2c%22Animal+Experiment%22%2ct%2bscolang%2c%22English%22%2ct&sl=1001&s=%28%28TITLE-ABS-KEY%28%22peptides%22%29+OR+TITLE-ABS-KEY%28%22antioxidant+peptides%22%29+OR+TITLE-ABS-KEY+%28%22antimicrobial+peptides%22%29+OR+TITLE-ABS-KEY%28%22angiogenic+peptides%22%29+OR+TITLE-ABS-KEY+%28%22animal+peptides%22%29+OR+TITLE-ABS-KEY%28%22natural+peptides%22%29+OR+TITLE-ABS-KEY%28%22bioactive+peptides%22%29+OR+TITLE-ABS-KEY%28%22biological+peptides%22%29+OR+TITLE-ABS-KEY%28%22isolated+peptides%22%29+OR+TITLE-ABS-KEY%28%22extracted+peptides%22%29%29%29+AND+%28%28TITLE-ABS-KEY%28%22wound+healing%22%29+OR+TITLE-ABS-KEY%28%22regeneration%22%29+OR+TITLE-ABS-KEY%28%22skin+repair%22%29+OR+TITLE-ABS-KEY%28%22cutaneous+repair%22%29+OR+TITLE-ABS-KEY%28%22skin+healing%22%29+OR+TITLE-ABS-KEY%28%22cutaneous+healing%22%29%29%29+AND+%28%28TITLE-ABS-KEY%28%22skin%22%29+OR+TITLE-ABS-KEY%28%22dermis%22%29+OR+TITLE-ABS-KEY+%28%22epidermis%22%29+OR+TITLE-ABS-KEY%28%22subcutaneous+tissue%22%29+OR+TITLE-ABS-KEY%28%22hypodermis%22%29+OR+TITLE-ABS-KEY%28%22granulation+tissue%22%29+OR+TITLE-ABS-KEY%28%22keratinocytes%22%29+OR+TITLE-ABS-KEY%28%22fibroblasts%22%29+OR+TITLE-ABS-KEY+%28%22integumentary+system%22%29+OR+TITLE-ABS-KEY%28%22skin+injuries%22%29+OR+TITLE-ABS-KEY%28%22skin+fibrosis%22%29+OR+TITLE-ABS-KEY%28%22skin+scars%22%29%29%29&relpos=58&citeCnt=9&searchTerm=) \| \| [Electrospun poly(l-lactide)/zein nanofiber mats loaded with *Rana chensinensis* skin peptides for wound dressing.](https://www.ncbi.nlm.nih.gov/pubmed/27432415) \| \| Encapsulation of collagen mimetic peptide-tethered vancomycin liposomes in collagen-based scaffolds for infection control in wounds \| \| Evaluation of Small Molecular Polypeptides from the Mantle of *Pinctada Martensii* on Promoting Skin Wound Healing in Mice \| \| [Exendin-4 in combination with adipose-derived stem cells promotes angiogenesis and improves diabetic wound healing](https://www-scopus.ez35.periodicos.capes.gov.br/record/display.uri?eid=2-s2.0-85012982083&origin=resultslist&sort=plf-f&src=s&nlo=&nlr=&nls=&mltAll=t&sid=8f682cda9d1e11ee420f15ef65811fea&sot=comb&sdt=cl&cluster=scoexactkeywords%2c%22Animal+Experiment%22%2ct%2bscolang%2c%22English%22%2ct&sl=1001&s=%28%28TITLE-ABS-KEY%28%22peptides%22%29+OR+TITLE-ABS-KEY%28%22antioxidant+peptides%22%29+OR+TITLE-ABS-KEY+%28%22antimicrobial+peptides%22%29+OR+TITLE-ABS-KEY%28%22angiogenic+peptides%22%29+OR+TITLE-ABS-KEY+%28%22animal+peptides%22%29+OR+TITLE-ABS-KEY%28%22natural+peptides%22%29+OR+TITLE-ABS-KEY%28%22bioactive+peptides%22%29+OR+TITLE-ABS-KEY%28%22biological+peptides%22%29+OR+TITLE-ABS-KEY%28%22isolated+peptides%22%29+OR+TITLE-ABS-KEY%28%22extracted+peptides%22%29%29%29+AND+%28%28TITLE-ABS-KEY%28%22wound+healing%22%29+OR+TITLE-ABS-KEY%28%22regeneration%22%29+OR+TITLE-ABS-KEY%28%22skin+repair%22%29+OR+TITLE-ABS-KEY%28%22cutaneous+repair%22%29+OR+TITLE-ABS-KEY%28%22skin+healing%22%29+OR+TITLE-ABS-KEY%28%22cutaneous+healing%22%29%29%29+AND+%28%28TITLE-ABS-KEY%28%22skin%22%29+OR+TITLE-ABS-KEY%28%22dermis%22%29+OR+TITLE-ABS-KEY+%28%22epidermis%22%29+OR+TITLE-ABS-KEY%28%22subcutaneous+tissue%22%29+OR+TITLE-ABS-KEY%28%22hypodermis%22%29+OR+TITLE-ABS-KEY%28%22granulation+tissue%22%29+OR+TITLE-ABS-KEY%28%22keratinocytes%22%29+OR+TITLE-ABS-KEY%28%22fibroblasts%22%29+OR+TITLE-ABS-KEY+%28%22integumentary+system%22%29+OR+TITLE-ABS-KEY%28%22skin+injuries%22%29+OR+TITLE-ABS-KEY%28%22skin+fibrosis%22%29+OR+TITLE-ABS-KEY%28%22skin+scars%22%29%29%29&relpos=146&citeCnt=11&searchTerm=) \| \| [Fabrication of KR-12 peptide-containing hyaluronic acid immobilized fibrous eggshell membrane effectively kills multi-drug-resistant bacteria, promotes angiogenesis and accelerates re-epithelialization.](https://www.ncbi.nlm.nih.gov/pubmed/31190796) \| \| [Fragments of Nle3-angiotensin(1-7) accelerate healing in dermal models](https://www-scopus.ez35.periodicos.capes.gov.br/record/display.uri?eid=2-s2.0-33645061481&origin=resultslist&sort=plf-f&src=s&nlo=&nlr=&nls=&mltAll=t&sid=8f682cda9d1e11ee420f15ef65811fea&sot=comb&sdt=cl&cluster=scoexactkeywords%2c%22Animal+Experiment%22%2ct%2bscolang%2c%22English%22%2ct&sl=1001&s=%28%28TITLE-ABS-KEY%28%22peptides%22%29+OR+TITLE-ABS-KEY%28%22antioxidant+peptides%22%29+OR+TITLE-ABS-KEY+%28%22antimicrobial+peptides%22%29+OR+TITLE-ABS-KEY%28%22angiogenic+peptides%22%29+OR+TITLE-ABS-KEY+%28%22animal+peptides%22%29+OR+TITLE-ABS-KEY%28%22natural+peptides%22%29+OR+TITLE-ABS-KEY%28%22bioactive+peptides%22%29+OR+TITLE-ABS-KEY%28%22biological+peptides%22%29+OR+TITLE-ABS-KEY%28%22isolated+peptides%22%29+OR+TITLE-ABS-KEY%28%22extracted+peptides%22%29%29%29+AND+%28%28TITLE-ABS-KEY%28%22wound+healing%22%29+OR+TITLE-ABS-KEY%28%22regeneration%22%29+OR+TITLE-ABS-KEY%28%22skin+repair%22%29+OR+TITLE-ABS-KEY%28%22cutaneous+repair%22%29+OR+TITLE-ABS-KEY%28%22skin+healing%22%29+OR+TITLE-ABS-KEY%28%22cutaneous+healing%22%29%29%29+AND+%28%28TITLE-ABS-KEY%28%22skin%22%29+OR+TITLE-ABS-KEY%28%22dermis%22%29+OR+TITLE-ABS-KEY+%28%22epidermis%22%29+OR+TITLE-ABS-KEY%28%22subcutaneous+tissue%22%29+OR+TITLE-ABS-KEY%28%22hypodermis%22%29+OR+TITLE-ABS-KEY%28%22granulation+tissue%22%29+OR+TITLE-ABS-KEY%28%22keratinocytes%22%29+OR+TITLE-ABS-KEY%28%22fibroblasts%22%29+OR+TITLE-ABS-KEY+%28%22integumentary+system%22%29+OR+TITLE-ABS-KEY%28%22skin+injuries%22%29+OR+TITLE-ABS-KEY%28%22skin+fibrosis%22%29+OR+TITLE-ABS-KEY%28%22skin+scars%22%29%29%29&relpos=640&citeCnt=11&searchTerm=) \| \| [Heparin mimetic peptide nanofiber gel promotes regeneration of full thickness burn injury](https://www-scopus.ez35.periodicos.capes.gov.br/record/display.uri?eid=2-s2.0-85018287757&origin=resultslist&sort=plf-f&src=s&nlo=&nlr=&nls=&mltAll=t&sid=8f682cda9d1e11ee420f15ef65811fea&sot=comb&sdt=cl&cluster=scoexactkeywords%2c%22Animal+Experiment%22%2ct%2bscolang%2c%22English%22%2ct&sl=1001&s=%28%28TITLE-ABS-KEY%28%22peptides%22%29+OR+TITLE-ABS-KEY%28%22antioxidant+peptides%22%29+OR+TITLE-ABS-KEY+%28%22antimicrobial+peptides%22%29+OR+TITLE-ABS-KEY%28%22angiogenic+peptides%22%29+OR+TITLE-ABS-KEY+%28%22animal+peptides%22%29+OR+TITLE-ABS-KEY%28%22natural+peptides%22%29+OR+TITLE-ABS-KEY%28%22bioactive+peptides%22%29+OR+TITLE-ABS-KEY%28%22biological+peptides%22%29+OR+TITLE-ABS-KEY%28%22isolated+peptides%22%29+OR+TITLE-ABS-KEY%28%22extracted+peptides%22%29%29%29+AND+%28%28TITLE-ABS-KEY%28%22wound+healing%22%29+OR+TITLE-ABS-KEY%28%22regeneration%22%29+OR+TITLE-ABS-KEY%28%22skin+repair%22%29+OR+TITLE-ABS-KEY%28%22cutaneous+repair%22%29+OR+TITLE-ABS-KEY%28%22skin+healing%22%29+OR+TITLE-ABS-KEY%28%22cutaneous+healing%22%29%29%29+AND+%28%28TITLE-ABS-KEY%28%22skin%22%29+OR+TITLE-ABS-KEY%28%22dermis%22%29+OR+TITLE-ABS-KEY+%28%22epidermis%22%29+OR+TITLE-ABS-KEY%28%22subcutaneous+tissue%22%29+OR+TITLE-ABS-KEY%28%22hypodermis%22%29+OR+TITLE-ABS-KEY%28%22granulation+tissue%22%29+OR+TITLE-ABS-KEY%28%22keratinocytes%22%29+OR+TITLE-ABS-KEY%28%22fibroblasts%22%29+OR+TITLE-ABS-KEY+%28%22integumentary+system%22%29+OR+TITLE-ABS-KEY%28%22skin+injuries%22%29+OR+TITLE-ABS-KEY%28%22skin+fibrosis%22%29+OR+TITLE-ABS-KEY%28%22skin+scars%22%29%29%29&relpos=130&citeCnt=13&searchTerm=) \| \| [Histological evaluation of the effects of angiotensin peptides on wound repair in diabetic mice](https://www-scopus.ez35.periodicos.capes.gov.br/record/display.uri?eid=2-s2.0-0347062408&origin=resultslist&sort=plf-f&src=s&nlo=&nlr=&nls=&mltAll=t&sid=8f682cda9d1e11ee420f15ef65811fea&sot=comb&sdt=cl&cluster=scoexactkeywords%2c%22Animal+Experiment%22%2ct%2bscolang%2c%22English%22%2ct&sl=1001&s=%28%28TITLE-ABS-KEY%28%22peptides%22%29+OR+TITLE-ABS-KEY%28%22antioxidant+peptides%22%29+OR+TITLE-ABS-KEY+%28%22antimicrobial+peptides%22%29+OR+TITLE-ABS-KEY%28%22angiogenic+peptides%22%29+OR+TITLE-ABS-KEY+%28%22animal+peptides%22%29+OR+TITLE-ABS-KEY%28%22natural+peptides%22%29+OR+TITLE-ABS-KEY%28%22bioactive+peptides%22%29+OR+TITLE-ABS-KEY%28%22biological+peptides%22%29+OR+TITLE-ABS-KEY%28%22isolated+peptides%22%29+OR+TITLE-ABS-KEY%28%22extracted+peptides%22%29%29%29+AND+%28%28TITLE-ABS-KEY%28%22wound+healing%22%29+OR+TITLE-ABS-KEY%28%22regeneration%22%29+OR+TITLE-ABS-KEY%28%22skin+repair%22%29+OR+TITLE-ABS-KEY%28%22cutaneous+repair%22%29+OR+TITLE-ABS-KEY%28%22skin+healing%22%29+OR+TITLE-ABS-KEY%28%22cutaneous+healing%22%29%29%29+AND+%28%28TITLE-ABS-KEY%28%22skin%22%29+OR+TITLE-ABS-KEY%28%22dermis%22%29+OR+TITLE-ABS-KEY+%28%22epidermis%22%29+OR+TITLE-ABS-KEY%28%22subcutaneous+tissue%22%29+OR+TITLE-ABS-KEY%28%22hypodermis%22%29+OR+TITLE-ABS-KEY%28%22granulation+tissue%22%29+OR+TITLE-ABS-KEY%28%22keratinocytes%22%29+OR+TITLE-ABS-KEY%28%22fibroblasts%22%29+OR+TITLE-ABS-KEY+%28%22integumentary+system%22%29+OR+TITLE-ABS-KEY%28%22skin+injuries%22%29+OR+TITLE-ABS-KEY%28%22skin+fibrosis%22%29+OR+TITLE-ABS-KEY%28%22skin+scars%22%29%29%29&relpos=677&citeCnt=34&searchTerm=) \| \| [Identification and characterization of novel matrix-derived bioactive peptides: A role for collagenase from santyl1 ointment in post-debridement wound healing?](https://www-scopus.ez35.periodicos.capes.gov.br/record/display.uri?eid=2-s2.0-84982718248&origin=resultslist&sort=plf-f&src=s&nlo=&nlr=&nls=&mltAll=t&sid=8f682cda9d1e11ee420f15ef65811fea&sot=comb&sdt=cl&cluster=scoexactkeywords%2c%22Animal+Experiment%22%2ct%2bscolang%2c%22English%22%2ct&sl=1001&s=%28%28TITLE-ABS-KEY%28%22peptides%22%29+OR+TITLE-ABS-KEY%28%22antioxidant+peptides%22%29+OR+TITLE-ABS-KEY+%28%22antimicrobial+peptides%22%29+OR+TITLE-ABS-KEY%28%22angiogenic+peptides%22%29+OR+TITLE-ABS-KEY+%28%22animal+peptides%22%29+OR+TITLE-ABS-KEY%28%22natural+peptides%22%29+OR+TITLE-ABS-KEY%28%22bioactive+peptides%22%29+OR+TITLE-ABS-KEY%28%22biological+peptides%22%29+OR+TITLE-ABS-KEY%28%22isolated+peptides%22%29+OR+TITLE-ABS-KEY%28%22extracted+peptides%22%29%29%29+AND+%28%28TITLE-ABS-KEY%28%22wound+healing%22%29+OR+TITLE-ABS-KEY%28%22regeneration%22%29+OR+TITLE-ABS-KEY%28%22skin+repair%22%29+OR+TITLE-ABS-KEY%28%22cutaneous+repair%22%29+OR+TITLE-ABS-KEY%28%22skin+healing%22%29+OR+TITLE-ABS-KEY%28%22cutaneous+healing%22%29%29%29+AND+%28%28TITLE-ABS-KEY%28%22skin%22%29+OR+TITLE-ABS-KEY%28%22dermis%22%29+OR+TITLE-ABS-KEY+%28%22epidermis%22%29+OR+TITLE-ABS-KEY%28%22subcutaneous+tissue%22%29+OR+TITLE-ABS-KEY%28%22hypodermis%22%29+OR+TITLE-ABS-KEY%28%22granulation+tissue%22%29+OR+TITLE-ABS-KEY%28%22keratinocytes%22%29+OR+TITLE-ABS-KEY%28%22fibroblasts%22%29+OR+TITLE-ABS-KEY+%28%22integumentary+system%22%29+OR+TITLE-ABS-KEY%28%22skin+injuries%22%29+OR+TITLE-ABS-KEY%28%22skin+fibrosis%22%29+OR+TITLE-ABS-KEY%28%22skin+scars%22%29%29%29&relpos=185&citeCnt=7&searchTerm=) \| \| [Immobilized thrombin receptor agonist peptide accelerates wound healing in mice](https://www-scopus.ez35.periodicos.capes.gov.br/record/display.uri?eid=2-s2.0-0034779690&origin=resultslist&sort=plf-f&src=s&nlo=&nlr=&nls=&mltAll=t&sid=8f682cda9d1e11ee420f15ef65811fea&sot=comb&sdt=cl&cluster=scoexactkeywords%2c%22Animal+Experiment%22%2ct%2bscolang%2c%22English%22%2ct&sl=1001&s=%28%28TITLE-ABS-KEY%28%22peptides%22%29+OR+TITLE-ABS-KEY%28%22antioxidant+peptides%22%29+OR+TITLE-ABS-KEY+%28%22antimicrobial+peptides%22%29+OR+TITLE-ABS-KEY%28%22angiogenic+peptides%22%29+OR+TITLE-ABS-KEY+%28%22animal+peptides%22%29+OR+TITLE-ABS-KEY%28%22natural+peptides%22%29+OR+TITLE-ABS-KEY%28%22bioactive+peptides%22%29+OR+TITLE-ABS-KEY%28%22biological+peptides%22%29+OR+TITLE-ABS-KEY%28%22isolated+peptides%22%29+OR+TITLE-ABS-KEY%28%22extracted+peptides%22%29%29%29+AND+%28%28TITLE-ABS-KEY%28%22wound+healing%22%29+OR+TITLE-ABS-KEY%28%22regeneration%22%29+OR+TITLE-ABS-KEY%28%22skin+repair%22%29+OR+TITLE-ABS-KEY%28%22cutaneous+repair%22%29+OR+TITLE-ABS-KEY%28%22skin+healing%22%29+OR+TITLE-ABS-KEY%28%22cutaneous+healing%22%29%29%29+AND+%28%28TITLE-ABS-KEY%28%22skin%22%29+OR+TITLE-ABS-KEY%28%22dermis%22%29+OR+TITLE-ABS-KEY+%28%22epidermis%22%29+OR+TITLE-ABS-KEY%28%22subcutaneous+tissue%22%29+OR+TITLE-ABS-KEY%28%22hypodermis%22%29+OR+TITLE-ABS-KEY%28%22granulation+tissue%22%29+OR+TITLE-ABS-KEY%28%22keratinocytes%22%29+OR+TITLE-ABS-KEY%28%22fibroblasts%22%29+OR+TITLE-ABS-KEY+%28%22integumentary+system%22%29+OR+TITLE-ABS-KEY%28%22skin+injuries%22%29+OR+TITLE-ABS-KEY%28%22skin+fibrosis%22%29+OR+TITLE-ABS-KEY%28%22skin+scars%22%29%29%29&relpos=728&citeCnt=31&searchTerm=) \| \| [In situ gel-forming AP-57 peptide delivery system for cutaneous wound healing](https://www-scopus.ez35.periodicos.capes.gov.br/record/display.uri?eid=2-s2.0-84942564033&origin=resultslist&sort=plf-f&src=s&nlo=&nlr=&nls=&mltAll=t&sid=8f682cda9d1e11ee420f15ef65811fea&sot=comb&sdt=cl&cluster=scoexactkeywords%2c%22Animal+Experiment%22%2ct%2bscolang%2c%22English%22%2ct&sl=1001&s=%28%28TITLE-ABS-KEY%28%22peptides%22%29+OR+TITLE-ABS-KEY%28%22antioxidant+peptides%22%29+OR+TITLE-ABS-KEY+%28%22antimicrobial+peptides%22%29+OR+TITLE-ABS-KEY%28%22angiogenic+peptides%22%29+OR+TITLE-ABS-KEY+%28%22animal+peptides%22%29+OR+TITLE-ABS-KEY%28%22natural+peptides%22%29+OR+TITLE-ABS-KEY%28%22bioactive+peptides%22%29+OR+TITLE-ABS-KEY%28%22biological+peptides%22%29+OR+TITLE-ABS-KEY%28%22isolated+peptides%22%29+OR+TITLE-ABS-KEY%28%22extracted+peptides%22%29%29%29+AND+%28%28TITLE-ABS-KEY%28%22wound+healing%22%29+OR+TITLE-ABS-KEY%28%22regeneration%22%29+OR+TITLE-ABS-KEY%28%22skin+repair%22%29+OR+TITLE-ABS-KEY%28%22cutaneous+repair%22%29+OR+TITLE-ABS-KEY%28%22skin+healing%22%29+OR+TITLE-ABS-KEY%28%22cutaneous+healing%22%29%29%29+AND+%28%28TITLE-ABS-KEY%28%22skin%22%29+OR+TITLE-ABS-KEY%28%22dermis%22%29+OR+TITLE-ABS-KEY+%28%22epidermis%22%29+OR+TITLE-ABS-KEY%28%22subcutaneous+tissue%22%29+OR+TITLE-ABS-KEY%28%22hypodermis%22%29+OR+TITLE-ABS-KEY%28%22granulation+tissue%22%29+OR+TITLE-ABS-KEY%28%22keratinocytes%22%29+OR+TITLE-ABS-KEY%28%22fibroblasts%22%29+OR+TITLE-ABS-KEY+%28%22integumentary+system%22%29+OR+TITLE-ABS-KEY%28%22skin+injuries%22%29+OR+TITLE-ABS-KEY%28%22skin+fibrosis%22%29+OR+TITLE-ABS-KEY%28%22skin+scars%22%29%29%29&relpos=224&citeCnt=24&searchTerm=) \| \| [In vivo effects of tailored laminin-332 α3 conjugated scaffolds enhances wound healing: A histomorphometric analysis](https://www-scopus.ez35.periodicos.capes.gov.br/record/display.uri?eid=2-s2.0-84883242313&origin=resultslist&sort=plf-f&src=s&nlo=&nlr=&nls=&mltAll=t&sid=8f682cda9d1e11ee420f15ef65811fea&sot=comb&sdt=cl&cluster=scoexactkeywords%2c%22Animal+Experiment%22%2ct%2bscolang%2c%22English%22%2ct&sl=1001&s=%28%28TITLE-ABS-KEY%28%22peptides%22%29+OR+TITLE-ABS-KEY%28%22antioxidant+peptides%22%29+OR+TITLE-ABS-KEY+%28%22antimicrobial+peptides%22%29+OR+TITLE-ABS-KEY%28%22angiogenic+peptides%22%29+OR+TITLE-ABS-KEY+%28%22animal+peptides%22%29+OR+TITLE-ABS-KEY%28%22natural+peptides%22%29+OR+TITLE-ABS-KEY%28%22bioactive+peptides%22%29+OR+TITLE-ABS-KEY%28%22biological+peptides%22%29+OR+TITLE-ABS-KEY%28%22isolated+peptides%22%29+OR+TITLE-ABS-KEY%28%22extracted+peptides%22%29%29%29+AND+%28%28TITLE-ABS-KEY%28%22wound+healing%22%29+OR+TITLE-ABS-KEY%28%22regeneration%22%29+OR+TITLE-ABS-KEY%28%22skin+repair%22%29+OR+TITLE-ABS-KEY%28%22cutaneous+repair%22%29+OR+TITLE-ABS-KEY%28%22skin+healing%22%29+OR+TITLE-ABS-KEY%28%22cutaneous+healing%22%29%29%29+AND+%28%28TITLE-ABS-KEY%28%22skin%22%29+OR+TITLE-ABS-KEY%28%22dermis%22%29+OR+TITLE-ABS-KEY+%28%22epidermis%22%29+OR+TITLE-ABS-KEY%28%22subcutaneous+tissue%22%29+OR+TITLE-ABS-KEY%28%22hypodermis%22%29+OR+TITLE-ABS-KEY%28%22granulation+tissue%22%29+OR+TITLE-ABS-KEY%28%22keratinocytes%22%29+OR+TITLE-ABS-KEY%28%22fibroblasts%22%29+OR+TITLE-ABS-KEY+%28%22integumentary+system%22%29+OR+TITLE-ABS-KEY%28%22skin+injuries%22%29+OR+TITLE-ABS-KEY%28%22skin+fibrosis%22%29+OR+TITLE-ABS-KEY%28%22skin+scars%22%29%29%29&relpos=339&citeCnt=8&searchTerm=) \| \| [Laminin heparin-binding peptides bind to several growth factors and enhance diabetic wound healing](https://www2.scopus.com/record/display.uri?eid=2-s2.0-85048076269&origin=resultslist&sort=plf-f&src=s&nlo=&nlr=&nls=&mltAll=t&sid=4d2dea855575f6fbdb403e5ce008a978&sot=comb&sdt=cl&cluster=scoexactkeywords%2c%22Animal+Experiment%22%2ct%2bscolang%2c%22English%22%2ct&sl=1001&s=%28%28TITLE-ABS-KEY%28%22peptides%22%29+OR+TITLE-ABS-KEY%28%22antioxidant+peptides%22%29+OR+TITLE-ABS-KEY+%28%22antimicrobial+peptides%22%29+OR+TITLE-ABS-KEY%28%22angiogenic+peptides%22%29+OR+TITLE-ABS-KEY+%28%22animal+peptides%22%29+OR+TITLE-ABS-KEY%28%22natural+peptides%22%29+OR+TITLE-ABS-KEY%28%22bioactive+peptides%22%29+OR+TITLE-ABS-KEY%28%22biological+peptides%22%29+OR+TITLE-ABS-KEY%28%22isolated+peptides%22%29+OR+TITLE-ABS-KEY%28%22extracted+peptides%22%29%29%29+AND+%28%28TITLE-ABS-KEY%28%22wound+healing%22%29+OR+TITLE-ABS-KEY%28%22regeneration%22%29+OR+TITLE-ABS-KEY%28%22skin+repair%22%29+OR+TITLE-ABS-KEY%28%22cutaneous+repair%22%29+OR+TITLE-ABS-KEY%28%22skin+healing%22%29+OR+TITLE-ABS-KEY%28%22cutaneous+healing%22%29%29%29+AND+%28%28TITLE-ABS-KEY%28%22skin%22%29+OR+TITLE-ABS-KEY%28%22dermis%22%29+OR+TITLE-ABS-KEY+%28%22epidermis%22%29+OR+TITLE-ABS-KEY%28%22subcutaneous+tissue%22%29+OR+TITLE-ABS-KEY%28%22hypodermis%22%29+OR+TITLE-ABS-KEY%28%22granulation+tissue%22%29+OR+TITLE-ABS-KEY%28%22keratinocytes%22%29+OR+TITLE-ABS-KEY%28%22fibroblasts%22%29+OR+TITLE-ABS-KEY+%28%22integumentary+system%22%29+OR+TITLE-ABS-KEY%28%22skin+injuries%22%29+OR+TITLE-ABS-KEY%28%22skin+fibrosis%22%29+OR+TITLE-ABS-KEY%28%22skin+scars%22%29%29%29&relpos=26&citeCnt=13&searchTerm=) \| \| [Laminin peptide-conjugated chitosan membrane: Application for keratinocyte delivery in wounded skin](https://www-scopus.ez35.periodicos.capes.gov.br/record/display.uri?eid=2-s2.0-33751566549&origin=resultslist&sort=plf-f&src=s&nlo=&nlr=&nls=&mltAll=t&sid=8f682cda9d1e11ee420f15ef65811fea&sot=comb&sdt=cl&cluster=scoexactkeywords%2c%22Animal+Experiment%22%2ct%2bscolang%2c%22English%22%2ct&sl=1001&s=%28%28TITLE-ABS-KEY%28%22peptides%22%29+OR+TITLE-ABS-KEY%28%22antioxidant+peptides%22%29+OR+TITLE-ABS-KEY+%28%22antimicrobial+peptides%22%29+OR+TITLE-ABS-KEY%28%22angiogenic+peptides%22%29+OR+TITLE-ABS-KEY+%28%22animal+peptides%22%29+OR+TITLE-ABS-KEY%28%22natural+peptides%22%29+OR+TITLE-ABS-KEY%28%22bioactive+peptides%22%29+OR+TITLE-ABS-KEY%28%22biological+peptides%22%29+OR+TITLE-ABS-KEY%28%22isolated+peptides%22%29+OR+TITLE-ABS-KEY%28%22extracted+peptides%22%29%29%29+AND+%28%28TITLE-ABS-KEY%28%22wound+healing%22%29+OR+TITLE-ABS-KEY%28%22regeneration%22%29+OR+TITLE-ABS-KEY%28%22skin+repair%22%29+OR+TITLE-ABS-KEY%28%22cutaneous+repair%22%29+OR+TITLE-ABS-KEY%28%22skin+healing%22%29+OR+TITLE-ABS-KEY%28%22cutaneous+healing%22%29%29%29+AND+%28%28TITLE-ABS-KEY%28%22skin%22%29+OR+TITLE-ABS-KEY%28%22dermis%22%29+OR+TITLE-ABS-KEY+%28%22epidermis%22%29+OR+TITLE-ABS-KEY%28%22subcutaneous+tissue%22%29+OR+TITLE-ABS-KEY%28%22hypodermis%22%29+OR+TITLE-ABS-KEY%28%22granulation+tissue%22%29+OR+TITLE-ABS-KEY%28%22keratinocytes%22%29+OR+TITLE-ABS-KEY%28%22fibroblasts%22%29+OR+TITLE-ABS-KEY+%28%22integumentary+system%22%29+OR+TITLE-ABS-KEY%28%22skin+injuries%22%29+OR+TITLE-ABS-KEY%28%22skin+fibrosis%22%29+OR+TITLE-ABS-KEY%28%22skin+scars%22%29%29%29&relpos=617&citeCnt=36&searchTerm=) \| \| Light-Activated Peptide-Based Materials for Sutureless Wound Closure \| \| [LL37 loaded nanostructured lipid carriers (NLC): A new strategy for the topical treatment of chronic wounds](https://www-scopus.ez35.periodicos.capes.gov.br/record/display.uri?eid=2-s2.0-84963642592&origin=resultslist&sort=plf-f&src=s&nlo=&nlr=&nls=&mltAll=t&sid=8f682cda9d1e11ee420f15ef65811fea&sot=comb&sdt=cl&cluster=scoexactkeywords%2c%22Animal+Experiment%22%2ct%2bscolang%2c%22English%22%2ct&sl=1001&s=%28%28TITLE-ABS-KEY%28%22peptides%22%29+OR+TITLE-ABS-KEY%28%22antioxidant+peptides%22%29+OR+TITLE-ABS-KEY+%28%22antimicrobial+peptides%22%29+OR+TITLE-ABS-KEY%28%22angiogenic+peptides%22%29+OR+TITLE-ABS-KEY+%28%22animal+peptides%22%29+OR+TITLE-ABS-KEY%28%22natural+peptides%22%29+OR+TITLE-ABS-KEY%28%22bioactive+peptides%22%29+OR+TITLE-ABS-KEY%28%22biological+peptides%22%29+OR+TITLE-ABS-KEY%28%22isolated+peptides%22%29+OR+TITLE-ABS-KEY%28%22extracted+peptides%22%29%29%29+AND+%28%28TITLE-ABS-KEY%28%22wound+healing%22%29+OR+TITLE-ABS-KEY%28%22regeneration%22%29+OR+TITLE-ABS-KEY%28%22skin+repair%22%29+OR+TITLE-ABS-KEY%28%22cutaneous+repair%22%29+OR+TITLE-ABS-KEY%28%22skin+healing%22%29+OR+TITLE-ABS-KEY%28%22cutaneous+healing%22%29%29%29+AND+%28%28TITLE-ABS-KEY%28%22skin%22%29+OR+TITLE-ABS-KEY%28%22dermis%22%29+OR+TITLE-ABS-KEY+%28%22epidermis%22%29+OR+TITLE-ABS-KEY%28%22subcutaneous+tissue%22%29+OR+TITLE-ABS-KEY%28%22hypodermis%22%29+OR+TITLE-ABS-KEY%28%22granulation+tissue%22%29+OR+TITLE-ABS-KEY%28%22keratinocytes%22%29+OR+TITLE-ABS-KEY%28%22fibroblasts%22%29+OR+TITLE-ABS-KEY+%28%22integumentary+system%22%29+OR+TITLE-ABS-KEY%28%22skin+injuries%22%29+OR+TITLE-ABS-KEY%28%22skin+fibrosis%22%29+OR+TITLE-ABS-KEY%28%22skin+scars%22%29%29%29&relpos=167&citeCnt=24&searchTerm=) \| \| [Matrix- and plasma-derived peptides promote tissue-specific injury responses and wound healing in diabetic swine](https://www-scopus.ez35.periodicos.capes.gov.br/record/display.uri?eid=2-s2.0-84977104329&origin=resultslist&sort=plf-f&src=s&nlo=&nlr=&nls=&mltAll=t&sid=8f682cda9d1e11ee420f15ef65811fea&sot=comb&sdt=cl&cluster=scoexactkeywords%2c%22Animal+Experiment%22%2ct%2bscolang%2c%22English%22%2ct&sl=1001&s=%28%28TITLE-ABS-KEY%28%22peptides%22%29+OR+TITLE-ABS-KEY%28%22antioxidant+peptides%22%29+OR+TITLE-ABS-KEY+%28%22antimicrobial+peptides%22%29+OR+TITLE-ABS-KEY%28%22angiogenic+peptides%22%29+OR+TITLE-ABS-KEY+%28%22animal+peptides%22%29+OR+TITLE-ABS-KEY%28%22natural+peptides%22%29+OR+TITLE-ABS-KEY%28%22bioactive+peptides%22%29+OR+TITLE-ABS-KEY%28%22biological+peptides%22%29+OR+TITLE-ABS-KEY%28%22isolated+peptides%22%29+OR+TITLE-ABS-KEY%28%22extracted+peptides%22%29%29%29+AND+%28%28TITLE-ABS-KEY%28%22wound+healing%22%29+OR+TITLE-ABS-KEY%28%22regeneration%22%29+OR+TITLE-ABS-KEY%28%22skin+repair%22%29+OR+TITLE-ABS-KEY%28%22cutaneous+repair%22%29+OR+TITLE-ABS-KEY%28%22skin+healing%22%29+OR+TITLE-ABS-KEY%28%22cutaneous+healing%22%29%29%29+AND+%28%28TITLE-ABS-KEY%28%22skin%22%29+OR+TITLE-ABS-KEY%28%22dermis%22%29+OR+TITLE-ABS-KEY+%28%22epidermis%22%29+OR+TITLE-ABS-KEY%28%22subcutaneous+tissue%22%29+OR+TITLE-ABS-KEY%28%22hypodermis%22%29+OR+TITLE-ABS-KEY%28%22granulation+tissue%22%29+OR+TITLE-ABS-KEY%28%22keratinocytes%22%29+OR+TITLE-ABS-KEY%28%22fibroblasts%22%29+OR+TITLE-ABS-KEY+%28%22integumentary+system%22%29+OR+TITLE-ABS-KEY%28%22skin+injuries%22%29+OR+TITLE-ABS-KEY%28%22skin+fibrosis%22%29+OR+TITLE-ABS-KEY%28%22skin+scars%22%29%29%29&relpos=182&citeCnt=11&searchTerm=) \| \| [Modeling Glyco-Collagen Conjugates Using a Host-Guest Strategy To Alter Phenotypic Cell Migration and in Vivo Wound Healing](https://www.ncbi.nlm.nih.gov/pubmed/29077384) \| \| [Multidomain Peptide Hydrogel Accelerates Healing of Full-Thickness Wounds in Diabetic Mice](https://www2.scopus.com/record/display.uri?eid=2-s2.0-85045187349&origin=resultslist&sort=plf-f&src=s&nlo=&nlr=&nls=&mltAll=t&sid=4d2dea855575f6fbdb403e5ce008a978&sot=comb&sdt=cl&cluster=scoexactkeywords%2c%22Animal+Experiment%22%2ct%2bscolang%2c%22English%22%2ct&sl=1001&s=%28%28TITLE-ABS-KEY%28%22peptides%22%29+OR+TITLE-ABS-KEY%28%22antioxidant+peptides%22%29+OR+TITLE-ABS-KEY+%28%22antimicrobial+peptides%22%29+OR+TITLE-ABS-KEY%28%22angiogenic+peptides%22%29+OR+TITLE-ABS-KEY+%28%22animal+peptides%22%29+OR+TITLE-ABS-KEY%28%22natural+peptides%22%29+OR+TITLE-ABS-KEY%28%22bioactive+peptides%22%29+OR+TITLE-ABS-KEY%28%22biological+peptides%22%29+OR+TITLE-ABS-KEY%28%22isolated+peptides%22%29+OR+TITLE-ABS-KEY%28%22extracted+peptides%22%29%29%29+AND+%28%28TITLE-ABS-KEY%28%22wound+healing%22%29+OR+TITLE-ABS-KEY%28%22regeneration%22%29+OR+TITLE-ABS-KEY%28%22skin+repair%22%29+OR+TITLE-ABS-KEY%28%22cutaneous+repair%22%29+OR+TITLE-ABS-KEY%28%22skin+healing%22%29+OR+TITLE-ABS-KEY%28%22cutaneous+healing%22%29%29%29+AND+%28%28TITLE-ABS-KEY%28%22skin%22%29+OR+TITLE-ABS-KEY%28%22dermis%22%29+OR+TITLE-ABS-KEY+%28%22epidermis%22%29+OR+TITLE-ABS-KEY%28%22subcutaneous+tissue%22%29+OR+TITLE-ABS-KEY%28%22hypodermis%22%29+OR+TITLE-ABS-KEY%28%22granulation+tissue%22%29+OR+TITLE-ABS-KEY%28%22keratinocytes%22%29+OR+TITLE-ABS-KEY%28%22fibroblasts%22%29+OR+TITLE-ABS-KEY+%28%22integumentary+system%22%29+OR+TITLE-ABS-KEY%28%22skin+injuries%22%29+OR+TITLE-ABS-KEY%28%22skin+fibrosis%22%29+OR+TITLE-ABS-KEY%28%22skin+scars%22%29%29%29&relpos=71&citeCnt=5&searchTerm=) \| \| [Oligoarginine mediated collagen/chitosan gel composite for cutaneous wound healing](https://www2.scopus.com/record/display.uri?eid=2-s2.0-85053332667&origin=resultslist&sort=plf-f&src=s&nlo=&nlr=&nls=&mltAll=t&sid=4d2dea855575f6fbdb403e5ce008a978&sot=comb&sdt=cl&cluster=scoexactkeywords%2c%22Animal+Experiment%22%2ct%2bscolang%2c%22English%22%2ct&sl=1001&s=%28%28TITLE-ABS-KEY%28%22peptides%22%29+OR+TITLE-ABS-KEY%28%22antioxidant+peptides%22%29+OR+TITLE-ABS-KEY+%28%22antimicrobial+peptides%22%29+OR+TITLE-ABS-KEY%28%22angiogenic+peptides%22%29+OR+TITLE-ABS-KEY+%28%22animal+peptides%22%29+OR+TITLE-ABS-KEY%28%22natural+peptides%22%29+OR+TITLE-ABS-KEY%28%22bioactive+peptides%22%29+OR+TITLE-ABS-KEY%28%22biological+peptides%22%29+OR+TITLE-ABS-KEY%28%22isolated+peptides%22%29+OR+TITLE-ABS-KEY%28%22extracted+peptides%22%29%29%29+AND+%28%28TITLE-ABS-KEY%28%22wound+healing%22%29+OR+TITLE-ABS-KEY%28%22regeneration%22%29+OR+TITLE-ABS-KEY%28%22skin+repair%22%29+OR+TITLE-ABS-KEY%28%22cutaneous+repair%22%29+OR+TITLE-ABS-KEY%28%22skin+healing%22%29+OR+TITLE-ABS-KEY%28%22cutaneous+healing%22%29%29%29+AND+%28%28TITLE-ABS-KEY%28%22skin%22%29+OR+TITLE-ABS-KEY%28%22dermis%22%29+OR+TITLE-ABS-KEY+%28%22epidermis%22%29+OR+TITLE-ABS-KEY%28%22subcutaneous+tissue%22%29+OR+TITLE-ABS-KEY%28%22hypodermis%22%29+OR+TITLE-ABS-KEY%28%22granulation+tissue%22%29+OR+TITLE-ABS-KEY%28%22keratinocytes%22%29+OR+TITLE-ABS-KEY%28%22fibroblasts%22%29+OR+TITLE-ABS-KEY+%28%22integumentary+system%22%29+OR+TITLE-ABS-KEY%28%22skin+injuries%22%29+OR+TITLE-ABS-KEY%28%22skin+fibrosis%22%29+OR+TITLE-ABS-KEY%28%22skin+scars%22%29%29%29&relpos=12&citeCnt=1&searchTerm=) \| \| [Pentadecapeptide BPC 157 cream improves burn-wound healing and attenuates burn-gastric lesions in mice](https://www-scopus.ez35.periodicos.capes.gov.br/record/display.uri?eid=2-s2.0-0035148759&origin=resultslist&sort=plf-f&src=s&nlo=&nlr=&nls=&mltAll=t&sid=8f682cda9d1e11ee420f15ef65811fea&sot=comb&sdt=cl&cluster=scoexactkeywords%2c%22Animal+Experiment%22%2ct%2bscolang%2c%22English%22%2ct&sl=1001&s=%28%28TITLE-ABS-KEY%28%22peptides%22%29+OR+TITLE-ABS-KEY%28%22antioxidant+peptides%22%29+OR+TITLE-ABS-KEY+%28%22antimicrobial+peptides%22%29+OR+TITLE-ABS-KEY%28%22angiogenic+peptides%22%29+OR+TITLE-ABS-KEY+%28%22animal+peptides%22%29+OR+TITLE-ABS-KEY%28%22natural+peptides%22%29+OR+TITLE-ABS-KEY%28%22bioactive+peptides%22%29+OR+TITLE-ABS-KEY%28%22biological+peptides%22%29+OR+TITLE-ABS-KEY%28%22isolated+peptides%22%29+OR+TITLE-ABS-KEY%28%22extracted+peptides%22%29%29%29+AND+%28%28TITLE-ABS-KEY%28%22wound+healing%22%29+OR+TITLE-ABS-KEY%28%22regeneration%22%29+OR+TITLE-ABS-KEY%28%22skin+repair%22%29+OR+TITLE-ABS-KEY%28%22cutaneous+repair%22%29+OR+TITLE-ABS-KEY%28%22skin+healing%22%29+OR+TITLE-ABS-KEY%28%22cutaneous+healing%22%29%29%29+AND+%28%28TITLE-ABS-KEY%28%22skin%22%29+OR+TITLE-ABS-KEY%28%22dermis%22%29+OR+TITLE-ABS-KEY+%28%22epidermis%22%29+OR+TITLE-ABS-KEY%28%22subcutaneous+tissue%22%29+OR+TITLE-ABS-KEY%28%22hypodermis%22%29+OR+TITLE-ABS-KEY%28%22granulation+tissue%22%29+OR+TITLE-ABS-KEY%28%22keratinocytes%22%29+OR+TITLE-ABS-KEY%28%22fibroblasts%22%29+OR+TITLE-ABS-KEY+%28%22integumentary+system%22%29+OR+TITLE-ABS-KEY%28%22skin+injuries%22%29+OR+TITLE-ABS-KEY%28%22skin+fibrosis%22%29+OR+TITLE-ABS-KEY%28%22skin+scars%22%29%29%29&relpos=732&citeCnt=58&searchTerm=) \| \| [Peptide-Modified Chitosan Hydrogels Accelerate Skin Wound Healing by Promoting Fibroblast Proliferation, Migration, and Secretion](https://www-scopus.ez35.periodicos.capes.gov.br/record/display.uri?eid=2-s2.0-85029436247&origin=resultslist&sort=plf-f&src=s&nlo=&nlr=&nls=&mltAll=t&sid=8f682cda9d1e11ee420f15ef65811fea&sot=comb&sdt=cl&cluster=scoexactkeywords%2c%22Animal+Experiment%22%2ct%2bscolang%2c%22English%22%2ct&sl=1001&s=%28%28TITLE-ABS-KEY%28%22peptides%22%29+OR+TITLE-ABS-KEY%28%22antioxidant+peptides%22%29+OR+TITLE-ABS-KEY+%28%22antimicrobial+peptides%22%29+OR+TITLE-ABS-KEY%28%22angiogenic+peptides%22%29+OR+TITLE-ABS-KEY+%28%22animal+peptides%22%29+OR+TITLE-ABS-KEY%28%22natural+peptides%22%29+OR+TITLE-ABS-KEY%28%22bioactive+peptides%22%29+OR+TITLE-ABS-KEY%28%22biological+peptides%22%29+OR+TITLE-ABS-KEY%28%22isolated+peptides%22%29+OR+TITLE-ABS-KEY%28%22extracted+peptides%22%29%29%29+AND+%28%28TITLE-ABS-KEY%28%22wound+healing%22%29+OR+TITLE-ABS-KEY%28%22regeneration%22%29+OR+TITLE-ABS-KEY%28%22skin+repair%22%29+OR+TITLE-ABS-KEY%28%22cutaneous+repair%22%29+OR+TITLE-ABS-KEY%28%22skin+healing%22%29+OR+TITLE-ABS-KEY%28%22cutaneous+healing%22%29%29%29+AND+%28%28TITLE-ABS-KEY%28%22skin%22%29+OR+TITLE-ABS-KEY%28%22dermis%22%29+OR+TITLE-ABS-KEY+%28%22epidermis%22%29+OR+TITLE-ABS-KEY%28%22subcutaneous+tissue%22%29+OR+TITLE-ABS-KEY%28%22hypodermis%22%29+OR+TITLE-ABS-KEY%28%22granulation+tissue%22%29+OR+TITLE-ABS-KEY%28%22keratinocytes%22%29+OR+TITLE-ABS-KEY%28%22fibroblasts%22%29+OR+TITLE-ABS-KEY+%28%22integumentary+system%22%29+OR+TITLE-ABS-KEY%28%22skin+injuries%22%29+OR+TITLE-ABS-KEY%28%22skin+fibrosis%22%29+OR+TITLE-ABS-KEY%28%22skin+scars%22%29%29%29&relpos=125&citeCnt=5&searchTerm=) \| \| [Peptide-modified chitosan hydrogels promote skin wound healing by enhancing wound angiogenesis and inhibiting inflammation](https://www-scopus.ez35.periodicos.capes.gov.br/record/display.uri?eid=2-s2.0-85019704502&origin=resultslist&sort=plf-f&src=s&nlo=&nlr=&nls=&mltAll=t&sid=8f682cda9d1e11ee420f15ef65811fea&sot=comb&sdt=cl&cluster=scoexactkeywords%2c%22Animal+Experiment%22%2ct%2bscolang%2c%22English%22%2ct&sl=1001&s=%28%28TITLE-ABS-KEY%28%22peptides%22%29+OR+TITLE-ABS-KEY%28%22antioxidant+peptides%22%29+OR+TITLE-ABS-KEY+%28%22antimicrobial+peptides%22%29+OR+TITLE-ABS-KEY%28%22angiogenic+peptides%22%29+OR+TITLE-ABS-KEY+%28%22animal+peptides%22%29+OR+TITLE-ABS-KEY%28%22natural+peptides%22%29+OR+TITLE-ABS-KEY%28%22bioactive+peptides%22%29+OR+TITLE-ABS-KEY%28%22biological+peptides%22%29+OR+TITLE-ABS-KEY%28%22isolated+peptides%22%29+OR+TITLE-ABS-KEY%28%22extracted+peptides%22%29%29%29+AND+%28%28TITLE-ABS-KEY%28%22wound+healing%22%29+OR+TITLE-ABS-KEY%28%22regeneration%22%29+OR+TITLE-ABS-KEY%28%22skin+repair%22%29+OR+TITLE-ABS-KEY%28%22cutaneous+repair%22%29+OR+TITLE-ABS-KEY%28%22skin+healing%22%29+OR+TITLE-ABS-KEY%28%22cutaneous+healing%22%29%29%29+AND+%28%28TITLE-ABS-KEY%28%22skin%22%29+OR+TITLE-ABS-KEY%28%22dermis%22%29+OR+TITLE-ABS-KEY+%28%22epidermis%22%29+OR+TITLE-ABS-KEY%28%22subcutaneous+tissue%22%29+OR+TITLE-ABS-KEY%28%22hypodermis%22%29+OR+TITLE-ABS-KEY%28%22granulation+tissue%22%29+OR+TITLE-ABS-KEY%28%22keratinocytes%22%29+OR+TITLE-ABS-KEY%28%22fibroblasts%22%29+OR+TITLE-ABS-KEY+%28%22integumentary+system%22%29+OR+TITLE-ABS-KEY%28%22skin+injuries%22%29+OR+TITLE-ABS-KEY%28%22skin+fibrosis%22%29+OR+TITLE-ABS-KEY%28%22skin+scars%22%29%29%29&relpos=152&citeCnt=9&searchTerm=) \| \| [Pexiganan-incorporated collagen matrices for infected wound-healing processes in rat](https://www-scopus.ez35.periodicos.capes.gov.br/record/display.uri?eid=2-s2.0-18644365611&origin=resultslist&sort=plf-f&src=s&nlo=&nlr=&nls=&mltAll=t&sid=8f682cda9d1e11ee420f15ef65811fea&sot=comb&sdt=cl&cluster=scoexactkeywords%2c%22Animal+Experiment%22%2ct%2bscolang%2c%22English%22%2ct&sl=1001&s=%28%28TITLE-ABS-KEY%28%22peptides%22%29+OR+TITLE-ABS-KEY%28%22antioxidant+peptides%22%29+OR+TITLE-ABS-KEY+%28%22antimicrobial+peptides%22%29+OR+TITLE-ABS-KEY%28%22angiogenic+peptides%22%29+OR+TITLE-ABS-KEY+%28%22animal+peptides%22%29+OR+TITLE-ABS-KEY%28%22natural+peptides%22%29+OR+TITLE-ABS-KEY%28%22bioactive+peptides%22%29+OR+TITLE-ABS-KEY%28%22biological+peptides%22%29+OR+TITLE-ABS-KEY%28%22isolated+peptides%22%29+OR+TITLE-ABS-KEY%28%22extracted+peptides%22%29%29%29+AND+%28%28TITLE-ABS-KEY%28%22wound+healing%22%29+OR+TITLE-ABS-KEY%28%22regeneration%22%29+OR+TITLE-ABS-KEY%28%22skin+repair%22%29+OR+TITLE-ABS-KEY%28%22cutaneous+repair%22%29+OR+TITLE-ABS-KEY%28%22skin+healing%22%29+OR+TITLE-ABS-KEY%28%22cutaneous+healing%22%29%29%29+AND+%28%28TITLE-ABS-KEY%28%22skin%22%29+OR+TITLE-ABS-KEY%28%22dermis%22%29+OR+TITLE-ABS-KEY+%28%22epidermis%22%29+OR+TITLE-ABS-KEY%28%22subcutaneous+tissue%22%29+OR+TITLE-ABS-KEY%28%22hypodermis%22%29+OR+TITLE-ABS-KEY%28%22granulation+tissue%22%29+OR+TITLE-ABS-KEY%28%22keratinocytes%22%29+OR+TITLE-ABS-KEY%28%22fibroblasts%22%29+OR+TITLE-ABS-KEY+%28%22integumentary+system%22%29+OR+TITLE-ABS-KEY%28%22skin+injuries%22%29+OR+TITLE-ABS-KEY%28%22skin+fibrosis%22%29+OR+TITLE-ABS-KEY%28%22skin+scars%22%29%29%29&relpos=645&citeCnt=35&searchTerm=) \| \| [Pigment epithelium-derived factor short peptides facilitate full-thickness cutaneous wound healing by promoting epithelial basal cell and hair follicle stem cell proliferation](https://www-scopus.ez35.periodicos.capes.gov.br/record/display.uri?eid=2-s2.0-85030234120&origin=resultslist&sort=plf-f&src=s&nlo=&nlr=&nls=&mltAll=t&sid=8f682cda9d1e11ee420f15ef65811fea&sot=comb&sdt=cl&cluster=scoexactkeywords%2c%22Animal+Experiment%22%2ct%2bscolang%2c%22English%22%2ct&sl=1001&s=%28%28TITLE-ABS-KEY%28%22peptides%22%29+OR+TITLE-ABS-KEY%28%22antioxidant+peptides%22%29+OR+TITLE-ABS-KEY+%28%22antimicrobial+peptides%22%29+OR+TITLE-ABS-KEY%28%22angiogenic+peptides%22%29+OR+TITLE-ABS-KEY+%28%22animal+peptides%22%29+OR+TITLE-ABS-KEY%28%22natural+peptides%22%29+OR+TITLE-ABS-KEY%28%22bioactive+peptides%22%29+OR+TITLE-ABS-KEY%28%22biological+peptides%22%29+OR+TITLE-ABS-KEY%28%22isolated+peptides%22%29+OR+TITLE-ABS-KEY%28%22extracted+peptides%22%29%29%29+AND+%28%28TITLE-ABS-KEY%28%22wound+healing%22%29+OR+TITLE-ABS-KEY%28%22regeneration%22%29+OR+TITLE-ABS-KEY%28%22skin+repair%22%29+OR+TITLE-ABS-KEY%28%22cutaneous+repair%22%29+OR+TITLE-ABS-KEY%28%22skin+healing%22%29+OR+TITLE-ABS-KEY%28%22cutaneous+healing%22%29%29%29+AND+%28%28TITLE-ABS-KEY%28%22skin%22%29+OR+TITLE-ABS-KEY%28%22dermis%22%29+OR+TITLE-ABS-KEY+%28%22epidermis%22%29+OR+TITLE-ABS-KEY%28%22subcutaneous+tissue%22%29+OR+TITLE-ABS-KEY%28%22hypodermis%22%29+OR+TITLE-ABS-KEY%28%22granulation+tissue%22%29+OR+TITLE-ABS-KEY%28%22keratinocytes%22%29+OR+TITLE-ABS-KEY%28%22fibroblasts%22%29+OR+TITLE-ABS-KEY+%28%22integumentary+system%22%29+OR+TITLE-ABS-KEY%28%22skin+injuries%22%29+OR+TITLE-ABS-KEY%28%22skin+fibrosis%22%29+OR+TITLE-ABS-KEY%28%22skin+scars%22%29%29%29&relpos=110&citeCnt=3&searchTerm=) \| \| [PLGA nanoparticles loaded with host defense peptide LL37 promote wound healing](https://www-scopus.ez35.periodicos.capes.gov.br/record/display.uri?eid=2-s2.0-84907226101&origin=resultslist&sort=plf-f&src=s&nlo=&nlr=&nls=&mltAll=t&sid=8f682cda9d1e11ee420f15ef65811fea&sot=comb&sdt=cl&cluster=scoexactkeywords%2c%22Animal+Experiment%22%2ct%2bscolang%2c%22English%22%2ct&sl=1001&s=%28%28TITLE-ABS-KEY%28%22peptides%22%29+OR+TITLE-ABS-KEY%28%22antioxidant+peptides%22%29+OR+TITLE-ABS-KEY+%28%22antimicrobial+peptides%22%29+OR+TITLE-ABS-KEY%28%22angiogenic+peptides%22%29+OR+TITLE-ABS-KEY+%28%22animal+peptides%22%29+OR+TITLE-ABS-KEY%28%22natural+peptides%22%29+OR+TITLE-ABS-KEY%28%22bioactive+peptides%22%29+OR+TITLE-ABS-KEY%28%22biological+peptides%22%29+OR+TITLE-ABS-KEY%28%22isolated+peptides%22%29+OR+TITLE-ABS-KEY%28%22extracted+peptides%22%29%29%29+AND+%28%28TITLE-ABS-KEY%28%22wound+healing%22%29+OR+TITLE-ABS-KEY%28%22regeneration%22%29+OR+TITLE-ABS-KEY%28%22skin+repair%22%29+OR+TITLE-ABS-KEY%28%22cutaneous+repair%22%29+OR+TITLE-ABS-KEY%28%22skin+healing%22%29+OR+TITLE-ABS-KEY%28%22cutaneous+healing%22%29%29%29+AND+%28%28TITLE-ABS-KEY%28%22skin%22%29+OR+TITLE-ABS-KEY%28%22dermis%22%29+OR+TITLE-ABS-KEY+%28%22epidermis%22%29+OR+TITLE-ABS-KEY%28%22subcutaneous+tissue%22%29+OR+TITLE-ABS-KEY%28%22hypodermis%22%29+OR+TITLE-ABS-KEY%28%22granulation+tissue%22%29+OR+TITLE-ABS-KEY%28%22keratinocytes%22%29+OR+TITLE-ABS-KEY%28%22fibroblasts%22%29+OR+TITLE-ABS-KEY+%28%22integumentary+system%22%29+OR+TITLE-ABS-KEY%28%22skin+injuries%22%29+OR+TITLE-ABS-KEY%28%22skin+fibrosis%22%29+OR+TITLE-ABS-KEY%28%22skin+scars%22%29%29%29&relpos=285&citeCnt=74&searchTerm=) \| \| [Polyvinyl alcohol nanofiber formulation of the designer antimicrobial peptide APO sterilizes *Acinetobacter baumannii*-infected skin wounds in mice](https://www-scopus.ez35.periodicos.capes.gov.br/record/display.uri?eid=2-s2.0-84955170510&origin=resultslist&sort=plf-f&src=s&nlo=&nlr=&nls=&mltAll=t&sid=8f682cda9d1e11ee420f15ef65811fea&sot=comb&sdt=cl&cluster=scoexactkeywords%2c%22Animal+Experiment%22%2ct%2bscolang%2c%22English%22%2ct&sl=1001&s=%28%28TITLE-ABS-KEY%28%22peptides%22%29+OR+TITLE-ABS-KEY%28%22antioxidant+peptides%22%29+OR+TITLE-ABS-KEY+%28%22antimicrobial+peptides%22%29+OR+TITLE-ABS-KEY%28%22angiogenic+peptides%22%29+OR+TITLE-ABS-KEY+%28%22animal+peptides%22%29+OR+TITLE-ABS-KEY%28%22natural+peptides%22%29+OR+TITLE-ABS-KEY%28%22bioactive+peptides%22%29+OR+TITLE-ABS-KEY%28%22biological+peptides%22%29+OR+TITLE-ABS-KEY%28%22isolated+peptides%22%29+OR+TITLE-ABS-KEY%28%22extracted+peptides%22%29%29%29+AND+%28%28TITLE-ABS-KEY%28%22wound+healing%22%29+OR+TITLE-ABS-KEY%28%22regeneration%22%29+OR+TITLE-ABS-KEY%28%22skin+repair%22%29+OR+TITLE-ABS-KEY%28%22cutaneous+repair%22%29+OR+TITLE-ABS-KEY%28%22skin+healing%22%29+OR+TITLE-ABS-KEY%28%22cutaneous+healing%22%29%29%29+AND+%28%28TITLE-ABS-KEY%28%22skin%22%29+OR+TITLE-ABS-KEY%28%22dermis%22%29+OR+TITLE-ABS-KEY+%28%22epidermis%22%29+OR+TITLE-ABS-KEY%28%22subcutaneous+tissue%22%29+OR+TITLE-ABS-KEY%28%22hypodermis%22%29+OR+TITLE-ABS-KEY%28%22granulation+tissue%22%29+OR+TITLE-ABS-KEY%28%22keratinocytes%22%29+OR+TITLE-ABS-KEY%28%22fibroblasts%22%29+OR+TITLE-ABS-KEY+%28%22integumentary+system%22%29+OR+TITLE-ABS-KEY%28%22skin+injuries%22%29+OR+TITLE-ABS-KEY%28%22skin+fibrosis%22%29+OR+TITLE-ABS-KEY%28%22skin+scars%22%29%29%29&relpos=208&citeCnt=17&searchTerm=) \| \| [Potential therapeutic efficacy of a bactericidal-immunomodulatory fusion peptide against methicillin-resistant Staphylococcus aureus skin infection](https://www-scopus.ez35.periodicos.capes.gov.br/record/display.uri?eid=2-s2.0-77149173760&origin=resultslist&sort=plf-f&src=s&nlo=&nlr=&nls=&mltAll=t&sid=8f682cda9d1e11ee420f15ef65811fea&sot=comb&sdt=cl&cluster=scoexactkeywords%2c%22Animal+Experiment%22%2ct%2bscolang%2c%22English%22%2ct&sl=1001&s=%28%28TITLE-ABS-KEY%28%22peptides%22%29+OR+TITLE-ABS-KEY%28%22antioxidant+peptides%22%29+OR+TITLE-ABS-KEY+%28%22antimicrobial+peptides%22%29+OR+TITLE-ABS-KEY%28%22angiogenic+peptides%22%29+OR+TITLE-ABS-KEY+%28%22animal+peptides%22%29+OR+TITLE-ABS-KEY%28%22natural+peptides%22%29+OR+TITLE-ABS-KEY%28%22bioactive+peptides%22%29+OR+TITLE-ABS-KEY%28%22biological+peptides%22%29+OR+TITLE-ABS-KEY%28%22isolated+peptides%22%29+OR+TITLE-ABS-KEY%28%22extracted+peptides%22%29%29%29+AND+%28%28TITLE-ABS-KEY%28%22wound+healing%22%29+OR+TITLE-ABS-KEY%28%22regeneration%22%29+OR+TITLE-ABS-KEY%28%22skin+repair%22%29+OR+TITLE-ABS-KEY%28%22cutaneous+repair%22%29+OR+TITLE-ABS-KEY%28%22skin+healing%22%29+OR+TITLE-ABS-KEY%28%22cutaneous+healing%22%29%29%29+AND+%28%28TITLE-ABS-KEY%28%22skin%22%29+OR+TITLE-ABS-KEY%28%22dermis%22%29+OR+TITLE-ABS-KEY+%28%22epidermis%22%29+OR+TITLE-ABS-KEY%28%22subcutaneous+tissue%22%29+OR+TITLE-ABS-KEY%28%22hypodermis%22%29+OR+TITLE-ABS-KEY%28%22granulation+tissue%22%29+OR+TITLE-ABS-KEY%28%22keratinocytes%22%29+OR+TITLE-ABS-KEY%28%22fibroblasts%22%29+OR+TITLE-ABS-KEY+%28%22integumentary+system%22%29+OR+TITLE-ABS-KEY%28%22skin+injuries%22%29+OR+TITLE-ABS-KEY%28%22skin+fibrosis%22%29+OR+TITLE-ABS-KEY%28%22skin+scars%22%29%29%29&relpos=491&citeCnt=10&searchTerm=) \| \| [Simultaneous dual syringe electrospinning system using benign solvent to fabricate nanofibrous P(3HB-co-4HB)/collagen peptides construct as potential leave-on wound dressing](https://www.ncbi.nlm.nih.gov/pubmed/27207048) \| \| [Skin Regeneration with a Scaffold of Predefined Shape and Bioactive Peptide Hydrogels](https://www2.scopus.com/record/display.uri?eid=2-s2.0-85054432761&origin=resultslist&sort=plf-f&src=s&nlo=&nlr=&nls=&mltAll=t&sid=4d2dea855575f6fbdb403e5ce008a978&sot=comb&sdt=cl&cluster=scoexactkeywords%2c%22Animal+Experiment%22%2ct%2bscolang%2c%22English%22%2ct&sl=1001&s=%28%28TITLE-ABS-KEY%28%22peptides%22%29+OR+TITLE-ABS-KEY%28%22antioxidant+peptides%22%29+OR+TITLE-ABS-KEY+%28%22antimicrobial+peptides%22%29+OR+TITLE-ABS-KEY%28%22angiogenic+peptides%22%29+OR+TITLE-ABS-KEY+%28%22animal+peptides%22%29+OR+TITLE-ABS-KEY%28%22natural+peptides%22%29+OR+TITLE-ABS-KEY%28%22bioactive+peptides%22%29+OR+TITLE-ABS-KEY%28%22biological+peptides%22%29+OR+TITLE-ABS-KEY%28%22isolated+peptides%22%29+OR+TITLE-ABS-KEY%28%22extracted+peptides%22%29%29%29+AND+%28%28TITLE-ABS-KEY%28%22wound+healing%22%29+OR+TITLE-ABS-KEY%28%22regeneration%22%29+OR+TITLE-ABS-KEY%28%22skin+repair%22%29+OR+TITLE-ABS-KEY%28%22cutaneous+repair%22%29+OR+TITLE-ABS-KEY%28%22skin+healing%22%29+OR+TITLE-ABS-KEY%28%22cutaneous+healing%22%29%29%29+AND+%28%28TITLE-ABS-KEY%28%22skin%22%29+OR+TITLE-ABS-KEY%28%22dermis%22%29+OR+TITLE-ABS-KEY+%28%22epidermis%22%29+OR+TITLE-ABS-KEY%28%22subcutaneous+tissue%22%29+OR+TITLE-ABS-KEY%28%22hypodermis%22%29+OR+TITLE-ABS-KEY%28%22granulation+tissue%22%29+OR+TITLE-ABS-KEY%28%22keratinocytes%22%29+OR+TITLE-ABS-KEY%28%22fibroblasts%22%29+OR+TITLE-ABS-KEY+%28%22integumentary+system%22%29+OR+TITLE-ABS-KEY%28%22skin+injuries%22%29+OR+TITLE-ABS-KEY%28%22skin+fibrosis%22%29+OR+TITLE-ABS-KEY%28%22skin+scars%22%29%29%29&relpos=40&citeCnt=5&searchTerm=) \| \| [Skin Regeneration with Self-Assembled Peptide Hydrogels Conjugated with Substance P in a Diabetic Rat Model](https://www-scopus.ez35.periodicos.capes.gov.br/record/display.uri?eid=2-s2.0-85040614169&origin=resultslist&sort=plf-f&src=s&nlo=&nlr=&nls=&mltAll=t&sid=8f682cda9d1e11ee420f15ef65811fea&sot=comb&sdt=cl&cluster=scoexactkeywords%2c%22Animal+Experiment%22%2ct%2bscolang%2c%22English%22%2ct&sl=1001&s=%28%28TITLE-ABS-KEY%28%22peptides%22%29+OR+TITLE-ABS-KEY%28%22antioxidant+peptides%22%29+OR+TITLE-ABS-KEY+%28%22antimicrobial+peptides%22%29+OR+TITLE-ABS-KEY%28%22angiogenic+peptides%22%29+OR+TITLE-ABS-KEY+%28%22animal+peptides%22%29+OR+TITLE-ABS-KEY%28%22natural+peptides%22%29+OR+TITLE-ABS-KEY%28%22bioactive+peptides%22%29+OR+TITLE-ABS-KEY%28%22biological+peptides%22%29+OR+TITLE-ABS-KEY%28%22isolated+peptides%22%29+OR+TITLE-ABS-KEY%28%22extracted+peptides%22%29%29%29+AND+%28%28TITLE-ABS-KEY%28%22wound+healing%22%29+OR+TITLE-ABS-KEY%28%22regeneration%22%29+OR+TITLE-ABS-KEY%28%22skin+repair%22%29+OR+TITLE-ABS-KEY%28%22cutaneous+repair%22%29+OR+TITLE-ABS-KEY%28%22skin+healing%22%29+OR+TITLE-ABS-KEY%28%22cutaneous+healing%22%29%29%29+AND+%28%28TITLE-ABS-KEY%28%22skin%22%29+OR+TITLE-ABS-KEY%28%22dermis%22%29+OR+TITLE-ABS-KEY+%28%22epidermis%22%29+OR+TITLE-ABS-KEY%28%22subcutaneous+tissue%22%29+OR+TITLE-ABS-KEY%28%22hypodermis%22%29+OR+TITLE-ABS-KEY%28%22granulation+tissue%22%29+OR+TITLE-ABS-KEY%28%22keratinocytes%22%29+OR+TITLE-ABS-KEY%28%22fibroblasts%22%29+OR+TITLE-ABS-KEY+%28%22integumentary+system%22%29+OR+TITLE-ABS-KEY%28%22skin+injuries%22%29+OR+TITLE-ABS-KEY%28%22skin+fibrosis%22%29+OR+TITLE-ABS-KEY%28%22skin+scars%22%29%29%29&relpos=92&citeCnt=7&searchTerm=) \| \| [Sponges of Carboxymethyl Chitosan Grafted with Collagen Peptides for Wound Healing.](https://www.ncbi.nlm.nih.gov/pubmed/31404991) \| \| [Stromal Cell-Derived Growth Factor-1 Alpha-Elastin Like Peptide Fusion Protein Promotes Cell Migration and Revascularization of Experimental Wounds in Diabetic Mice](https://www.ncbi.nlm.nih.gov/pubmed/28116224) \| \| [Substance P accelerates wound healing in type 2 diabetic mice through endothelial progenitor cell mobilization and Yes-associated protein activation](https://www-scopus.ez35.periodicos.capes.gov.br/record/display.uri?eid=2-s2.0-85018696118&origin=resultslist&sort=plf-f&src=s&nlo=&nlr=&nls=&mltAll=t&sid=8f682cda9d1e11ee420f15ef65811fea&sot=comb&sdt=cl&cluster=scoexactkeywords%2c%22Animal+Experiment%22%2ct%2bscolang%2c%22English%22%2ct&sl=1001&s=%28%28TITLE-ABS-KEY%28%22peptides%22%29+OR+TITLE-ABS-KEY%28%22antioxidant+peptides%22%29+OR+TITLE-ABS-KEY+%28%22antimicrobial+peptides%22%29+OR+TITLE-ABS-KEY%28%22angiogenic+peptides%22%29+OR+TITLE-ABS-KEY+%28%22animal+peptides%22%29+OR+TITLE-ABS-KEY%28%22natural+peptides%22%29+OR+TITLE-ABS-KEY%28%22bioactive+peptides%22%29+OR+TITLE-ABS-KEY%28%22biological+peptides%22%29+OR+TITLE-ABS-KEY%28%22isolated+peptides%22%29+OR+TITLE-ABS-KEY%28%22extracted+peptides%22%29%29%29+AND+%28%28TITLE-ABS-KEY%28%22wound+healing%22%29+OR+TITLE-ABS-KEY%28%22regeneration%22%29+OR+TITLE-ABS-KEY%28%22skin+repair%22%29+OR+TITLE-ABS-KEY%28%22cutaneous+repair%22%29+OR+TITLE-ABS-KEY%28%22skin+healing%22%29+OR+TITLE-ABS-KEY%28%22cutaneous+healing%22%29%29%29+AND+%28%28TITLE-ABS-KEY%28%22skin%22%29+OR+TITLE-ABS-KEY%28%22dermis%22%29+OR+TITLE-ABS-KEY+%28%22epidermis%22%29+OR+TITLE-ABS-KEY%28%22subcutaneous+tissue%22%29+OR+TITLE-ABS-KEY%28%22hypodermis%22%29+OR+TITLE-ABS-KEY%28%22granulation+tissue%22%29+OR+TITLE-ABS-KEY%28%22keratinocytes%22%29+OR+TITLE-ABS-KEY%28%22fibroblasts%22%29+OR+TITLE-ABS-KEY+%28%22integumentary+system%22%29+OR+TITLE-ABS-KEY%28%22skin+injuries%22%29+OR+TITLE-ABS-KEY%28%22skin+fibrosis%22%29+OR+TITLE-ABS-KEY%28%22skin+scars%22%29%29%29&relpos=160&citeCnt=3&searchTerm=) \| \| [Sustained-release adrenomedullin ointment accelerates wound healing of pressure ulcers](https://www-scopus.ez35.periodicos.capes.gov.br/record/display.uri?eid=2-s2.0-79955664444&origin=resultslist&sort=plf-f&src=s&nlo=&nlr=&nls=&mltAll=t&sid=8f682cda9d1e11ee420f15ef65811fea&sot=comb&sdt=cl&cluster=scoexactkeywords%2c%22Animal+Experiment%22%2ct%2bscolang%2c%22English%22%2ct&sl=1001&s=%28%28TITLE-ABS-KEY%28%22peptides%22%29+OR+TITLE-ABS-KEY%28%22antioxidant+peptides%22%29+OR+TITLE-ABS-KEY+%28%22antimicrobial+peptides%22%29+OR+TITLE-ABS-KEY%28%22angiogenic+peptides%22%29+OR+TITLE-ABS-KEY+%28%22animal+peptides%22%29+OR+TITLE-ABS-KEY%28%22natural+peptides%22%29+OR+TITLE-ABS-KEY%28%22bioactive+peptides%22%29+OR+TITLE-ABS-KEY%28%22biological+peptides%22%29+OR+TITLE-ABS-KEY%28%22isolated+peptides%22%29+OR+TITLE-ABS-KEY%28%22extracted+peptides%22%29%29%29+AND+%28%28TITLE-ABS-KEY%28%22wound+healing%22%29+OR+TITLE-ABS-KEY%28%22regeneration%22%29+OR+TITLE-ABS-KEY%28%22skin+repair%22%29+OR+TITLE-ABS-KEY%28%22cutaneous+repair%22%29+OR+TITLE-ABS-KEY%28%22skin+healing%22%29+OR+TITLE-ABS-KEY%28%22cutaneous+healing%22%29%29%29+AND+%28%28TITLE-ABS-KEY%28%22skin%22%29+OR+TITLE-ABS-KEY%28%22dermis%22%29+OR+TITLE-ABS-KEY+%28%22epidermis%22%29+OR+TITLE-ABS-KEY%28%22subcutaneous+tissue%22%29+OR+TITLE-ABS-KEY%28%22hypodermis%22%29+OR+TITLE-ABS-KEY%28%22granulation+tissue%22%29+OR+TITLE-ABS-KEY%28%22keratinocytes%22%29+OR+TITLE-ABS-KEY%28%22fibroblasts%22%29+OR+TITLE-ABS-KEY+%28%22integumentary+system%22%29+OR+TITLE-ABS-KEY%28%22skin+injuries%22%29+OR+TITLE-ABS-KEY%28%22skin+fibrosis%22%29+OR+TITLE-ABS-KEY%28%22skin+scars%22%29%29%29&relpos=445&citeCnt=10&searchTerm=) \| \| [Synthesis and assessment of a novel peptide conjugate to deliver phenytoin for wound repair](https://www-scopus.ez35.periodicos.capes.gov.br/record/display.uri?eid=2-s2.0-36048966064&origin=resultslist&sort=plf-f&src=s&nlo=&nlr=&nls=&mltAll=t&sid=8f682cda9d1e11ee420f15ef65811fea&sot=comb&sdt=cl&cluster=scoexactkeywords%2c%22Animal+Experiment%22%2ct%2bscolang%2c%22English%22%2ct&sl=1001&s=%28%28TITLE-ABS-KEY%28%22peptides%22%29+OR+TITLE-ABS-KEY%28%22antioxidant+peptides%22%29+OR+TITLE-ABS-KEY+%28%22antimicrobial+peptides%22%29+OR+TITLE-ABS-KEY%28%22angiogenic+peptides%22%29+OR+TITLE-ABS-KEY+%28%22animal+peptides%22%29+OR+TITLE-ABS-KEY%28%22natural+peptides%22%29+OR+TITLE-ABS-KEY%28%22bioactive+peptides%22%29+OR+TITLE-ABS-KEY%28%22biological+peptides%22%29+OR+TITLE-ABS-KEY%28%22isolated+peptides%22%29+OR+TITLE-ABS-KEY%28%22extracted+peptides%22%29%29%29+AND+%28%28TITLE-ABS-KEY%28%22wound+healing%22%29+OR+TITLE-ABS-KEY%28%22regeneration%22%29+OR+TITLE-ABS-KEY%28%22skin+repair%22%29+OR+TITLE-ABS-KEY%28%22cutaneous+repair%22%29+OR+TITLE-ABS-KEY%28%22skin+healing%22%29+OR+TITLE-ABS-KEY%28%22cutaneous+healing%22%29%29%29+AND+%28%28TITLE-ABS-KEY%28%22skin%22%29+OR+TITLE-ABS-KEY%28%22dermis%22%29+OR+TITLE-ABS-KEY+%28%22epidermis%22%29+OR+TITLE-ABS-KEY%28%22subcutaneous+tissue%22%29+OR+TITLE-ABS-KEY%28%22hypodermis%22%29+OR+TITLE-ABS-KEY%28%22granulation+tissue%22%29+OR+TITLE-ABS-KEY%28%22keratinocytes%22%29+OR+TITLE-ABS-KEY%28%22fibroblasts%22%29+OR+TITLE-ABS-KEY+%28%22integumentary+system%22%29+OR+TITLE-ABS-KEY%28%22skin+injuries%22%29+OR+TITLE-ABS-KEY%28%22skin+fibrosis%22%29+OR+TITLE-ABS-KEY%28%22skin+scars%22%29%29%29&relpos=612&citeCnt=0&searchTerm=) \| \| [TGF-β1 latency associated peptide promotes remodeling of healing cutaneous wounds in the rat](https://www-scopus.ez35.periodicos.capes.gov.br/record/display.uri?eid=2-s2.0-33744955987&origin=resultslist&sort=plf-f&src=s&nlo=&nlr=&nls=&mltAll=t&sid=8f682cda9d1e11ee420f15ef65811fea&sot=comb&sdt=cl&cluster=scoexactkeywords%2c%22Animal+Experiment%22%2ct%2bscolang%2c%22English%22%2ct&sl=1001&s=%28%28TITLE-ABS-KEY%28%22peptides%22%29+OR+TITLE-ABS-KEY%28%22antioxidant+peptides%22%29+OR+TITLE-ABS-KEY+%28%22antimicrobial+peptides%22%29+OR+TITLE-ABS-KEY%28%22angiogenic+peptides%22%29+OR+TITLE-ABS-KEY+%28%22animal+peptides%22%29+OR+TITLE-ABS-KEY%28%22natural+peptides%22%29+OR+TITLE-ABS-KEY%28%22bioactive+peptides%22%29+OR+TITLE-ABS-KEY%28%22biological+peptides%22%29+OR+TITLE-ABS-KEY%28%22isolated+peptides%22%29+OR+TITLE-ABS-KEY%28%22extracted+peptides%22%29%29%29+AND+%28%28TITLE-ABS-KEY%28%22wound+healing%22%29+OR+TITLE-ABS-KEY%28%22regeneration%22%29+OR+TITLE-ABS-KEY%28%22skin+repair%22%29+OR+TITLE-ABS-KEY%28%22cutaneous+repair%22%29+OR+TITLE-ABS-KEY%28%22skin+healing%22%29+OR+TITLE-ABS-KEY%28%22cutaneous+healing%22%29%29%29+AND+%28%28TITLE-ABS-KEY%28%22skin%22%29+OR+TITLE-ABS-KEY%28%22dermis%22%29+OR+TITLE-ABS-KEY+%28%22epidermis%22%29+OR+TITLE-ABS-KEY%28%22subcutaneous+tissue%22%29+OR+TITLE-ABS-KEY%28%22hypodermis%22%29+OR+TITLE-ABS-KEY%28%22granulation+tissue%22%29+OR+TITLE-ABS-KEY%28%22keratinocytes%22%29+OR+TITLE-ABS-KEY%28%22fibroblasts%22%29+OR+TITLE-ABS-KEY+%28%22integumentary+system%22%29+OR+TITLE-ABS-KEY%28%22skin+injuries%22%29+OR+TITLE-ABS-KEY%28%22skin+fibrosis%22%29+OR+TITLE-ABS-KEY%28%22skin+scars%22%29%29%29&relpos=633&citeCnt=3&searchTerm=) \| \| [The development and characterization of SDF1α-elastin-like-peptide nanoparticles for wound healing](https://www-scopus.ez35.periodicos.capes.gov.br/record/display.uri?eid=2-s2.0-84966270760&origin=resultslist&sort=plf-f&src=s&nlo=&nlr=&nls=&mltAll=t&sid=8f682cda9d1e11ee420f15ef65811fea&sot=comb&sdt=cl&cluster=scoexactkeywords%2c%22Animal+Experiment%22%2ct%2bscolang%2c%22English%22%2ct&sl=1001&s=%28%28TITLE-ABS-KEY%28%22peptides%22%29+OR+TITLE-ABS-KEY%28%22antioxidant+peptides%22%29+OR+TITLE-ABS-KEY+%28%22antimicrobial+peptides%22%29+OR+TITLE-ABS-KEY%28%22angiogenic+peptides%22%29+OR+TITLE-ABS-KEY+%28%22animal+peptides%22%29+OR+TITLE-ABS-KEY%28%22natural+peptides%22%29+OR+TITLE-ABS-KEY%28%22bioactive+peptides%22%29+OR+TITLE-ABS-KEY%28%22biological+peptides%22%29+OR+TITLE-ABS-KEY%28%22isolated+peptides%22%29+OR+TITLE-ABS-KEY%28%22extracted+peptides%22%29%29%29+AND+%28%28TITLE-ABS-KEY%28%22wound+healing%22%29+OR+TITLE-ABS-KEY%28%22regeneration%22%29+OR+TITLE-ABS-KEY%28%22skin+repair%22%29+OR+TITLE-ABS-KEY%28%22cutaneous+repair%22%29+OR+TITLE-ABS-KEY%28%22skin+healing%22%29+OR+TITLE-ABS-KEY%28%22cutaneous+healing%22%29%29%29+AND+%28%28TITLE-ABS-KEY%28%22skin%22%29+OR+TITLE-ABS-KEY%28%22dermis%22%29+OR+TITLE-ABS-KEY+%28%22epidermis%22%29+OR+TITLE-ABS-KEY%28%22subcutaneous+tissue%22%29+OR+TITLE-ABS-KEY%28%22hypodermis%22%29+OR+TITLE-ABS-KEY%28%22granulation+tissue%22%29+OR+TITLE-ABS-KEY%28%22keratinocytes%22%29+OR+TITLE-ABS-KEY%28%22fibroblasts%22%29+OR+TITLE-ABS-KEY+%28%22integumentary+system%22%29+OR+TITLE-ABS-KEY%28%22skin+injuries%22%29+OR+TITLE-ABS-KEY%28%22skin+fibrosis%22%29+OR+TITLE-ABS-KEY%28%22skin+scars%22%29%29%29&relpos=209&citeCnt=14&searchTerm=) \| \| [The effect of a laminin-5-derived peptide coated onto chitin microfibers on re-epithelialization in early-stage wound healing](https://www-scopus.ez35.periodicos.capes.gov.br/record/display.uri?eid=2-s2.0-77951026399&origin=resultslist&sort=plf-f&src=s&nlo=&nlr=&nls=&mltAll=t&sid=8f682cda9d1e11ee420f15ef65811fea&sot=comb&sdt=cl&cluster=scoexactkeywords%2c%22Animal+Experiment%22%2ct%2bscolang%2c%22English%22%2ct&sl=1001&s=%28%28TITLE-ABS-KEY%28%22peptides%22%29+OR+TITLE-ABS-KEY%28%22antioxidant+peptides%22%29+OR+TITLE-ABS-KEY+%28%22antimicrobial+peptides%22%29+OR+TITLE-ABS-KEY%28%22angiogenic+peptides%22%29+OR+TITLE-ABS-KEY+%28%22animal+peptides%22%29+OR+TITLE-ABS-KEY%28%22natural+peptides%22%29+OR+TITLE-ABS-KEY%28%22bioactive+peptides%22%29+OR+TITLE-ABS-KEY%28%22biological+peptides%22%29+OR+TITLE-ABS-KEY%28%22isolated+peptides%22%29+OR+TITLE-ABS-KEY%28%22extracted+peptides%22%29%29%29+AND+%28%28TITLE-ABS-KEY%28%22wound+healing%22%29+OR+TITLE-ABS-KEY%28%22regeneration%22%29+OR+TITLE-ABS-KEY%28%22skin+repair%22%29+OR+TITLE-ABS-KEY%28%22cutaneous+repair%22%29+OR+TITLE-ABS-KEY%28%22skin+healing%22%29+OR+TITLE-ABS-KEY%28%22cutaneous+healing%22%29%29%29+AND+%28%28TITLE-ABS-KEY%28%22skin%22%29+OR+TITLE-ABS-KEY%28%22dermis%22%29+OR+TITLE-ABS-KEY+%28%22epidermis%22%29+OR+TITLE-ABS-KEY%28%22subcutaneous+tissue%22%29+OR+TITLE-ABS-KEY%28%22hypodermis%22%29+OR+TITLE-ABS-KEY%28%22granulation+tissue%22%29+OR+TITLE-ABS-KEY%28%22keratinocytes%22%29+OR+TITLE-ABS-KEY%28%22fibroblasts%22%29+OR+TITLE-ABS-KEY+%28%22integumentary+system%22%29+OR+TITLE-ABS-KEY%28%22skin+injuries%22%29+OR+TITLE-ABS-KEY%28%22skin+fibrosis%22%29+OR+TITLE-ABS-KEY%28%22skin+scars%22%29%29%29&relpos=485&citeCnt=26&searchTerm=) \| \| [The effects of Antimicrobial Peptides and Hyaluronic Acid compound mask on wound healing after ablative fractional Carbon Dioxide laser resurfacing](https://www.ncbi.nlm.nih.gov/pubmed/30198801) \| \| The wound healing effects of the Tilapia collagen peptide mixture TY001 in streptozotocin diabetic mice \| \| [Therapeutic effects of a recombinant human collagen peptide bioscaffold with human adipose-derived stem cells on impaired wound healing after radiotherapy](https://www2.scopus.com/record/display.uri?eid=2-s2.0-85046764118&origin=resultslist&sort=plf-f&src=s&nlo=&nlr=&nls=&mltAll=t&sid=4d2dea855575f6fbdb403e5ce008a978&sot=comb&sdt=cl&cluster=scoexactkeywords%2c%22Animal+Experiment%22%2ct%2bscolang%2c%22English%22%2ct&sl=1001&s=%28%28TITLE-ABS-KEY%28%22peptides%22%29+OR+TITLE-ABS-KEY%28%22antioxidant+peptides%22%29+OR+TITLE-ABS-KEY+%28%22antimicrobial+peptides%22%29+OR+TITLE-ABS-KEY%28%22angiogenic+peptides%22%29+OR+TITLE-ABS-KEY+%28%22animal+peptides%22%29+OR+TITLE-ABS-KEY%28%22natural+peptides%22%29+OR+TITLE-ABS-KEY%28%22bioactive+peptides%22%29+OR+TITLE-ABS-KEY%28%22biological+peptides%22%29+OR+TITLE-ABS-KEY%28%22isolated+peptides%22%29+OR+TITLE-ABS-KEY%28%22extracted+peptides%22%29%29%29+AND+%28%28TITLE-ABS-KEY%28%22wound+healing%22%29+OR+TITLE-ABS-KEY%28%22regeneration%22%29+OR+TITLE-ABS-KEY%28%22skin+repair%22%29+OR+TITLE-ABS-KEY%28%22cutaneous+repair%22%29+OR+TITLE-ABS-KEY%28%22skin+healing%22%29+OR+TITLE-ABS-KEY%28%22cutaneous+healing%22%29%29%29+AND+%28%28TITLE-ABS-KEY%28%22skin%22%29+OR+TITLE-ABS-KEY%28%22dermis%22%29+OR+TITLE-ABS-KEY+%28%22epidermis%22%29+OR+TITLE-ABS-KEY%28%22subcutaneous+tissue%22%29+OR+TITLE-ABS-KEY%28%22hypodermis%22%29+OR+TITLE-ABS-KEY%28%22granulation+tissue%22%29+OR+TITLE-ABS-KEY%28%22keratinocytes%22%29+OR+TITLE-ABS-KEY%28%22fibroblasts%22%29+OR+TITLE-ABS-KEY+%28%22integumentary+system%22%29+OR+TITLE-ABS-KEY%28%22skin+injuries%22%29+OR+TITLE-ABS-KEY%28%22skin+fibrosis%22%29+OR+TITLE-ABS-KEY%28%22skin+scars%22%29%29%29&relpos=70&citeCnt=3&searchTerm=) \| \| [Thermosensitive biomimetic polyisocyanopeptide hydrogels may facilitate wound repair](https://www2.scopus.com/record/display.uri?eid=2-s2.0-85053223095&origin=resultslist&sort=plf-f&src=s&nlo=&nlr=&nls=&mltAll=t&sid=4d2dea855575f6fbdb403e5ce008a978&sot=comb&sdt=cl&cluster=scoexactkeywords%2c%22Animal+Experiment%22%2ct%2bscolang%2c%22English%22%2ct&sl=1001&s=%28%28TITLE-ABS-KEY%28%22peptides%22%29+OR+TITLE-ABS-KEY%28%22antioxidant+peptides%22%29+OR+TITLE-ABS-KEY+%28%22antimicrobial+peptides%22%29+OR+TITLE-ABS-KEY%28%22angiogenic+peptides%22%29+OR+TITLE-ABS-KEY+%28%22animal+peptides%22%29+OR+TITLE-ABS-KEY%28%22natural+peptides%22%29+OR+TITLE-ABS-KEY%28%22bioactive+peptides%22%29+OR+TITLE-ABS-KEY%28%22biological+peptides%22%29+OR+TITLE-ABS-KEY%28%22isolated+peptides%22%29+OR+TITLE-ABS-KEY%28%22extracted+peptides%22%29%29%29+AND+%28%28TITLE-ABS-KEY%28%22wound+healing%22%29+OR+TITLE-ABS-KEY%28%22regeneration%22%29+OR+TITLE-ABS-KEY%28%22skin+repair%22%29+OR+TITLE-ABS-KEY%28%22cutaneous+repair%22%29+OR+TITLE-ABS-KEY%28%22skin+healing%22%29+OR+TITLE-ABS-KEY%28%22cutaneous+healing%22%29%29%29+AND+%28%28TITLE-ABS-KEY%28%22skin%22%29+OR+TITLE-ABS-KEY%28%22dermis%22%29+OR+TITLE-ABS-KEY+%28%22epidermis%22%29+OR+TITLE-ABS-KEY%28%22subcutaneous+tissue%22%29+OR+TITLE-ABS-KEY%28%22hypodermis%22%29+OR+TITLE-ABS-KEY%28%22granulation+tissue%22%29+OR+TITLE-ABS-KEY%28%22keratinocytes%22%29+OR+TITLE-ABS-KEY%28%22fibroblasts%22%29+OR+TITLE-ABS-KEY+%28%22integumentary+system%22%29+OR+TITLE-ABS-KEY%28%22skin+injuries%22%29+OR+TITLE-ABS-KEY%28%22skin+fibrosis%22%29+OR+TITLE-ABS-KEY%28%22skin+scars%22%29%29%29&relpos=41&citeCnt=6&searchTerm=) \| \| [Ultrashort peptide nanofibrous hydrogels for the acceleration of healing of burn wounds](https://www-scopus.ez35.periodicos.capes.gov.br/record/display.uri?eid=2-s2.0-84896494731&origin=resultslist&sort=plf-f&src=s&nlo=&nlr=&nls=&mltAll=t&sid=8f682cda9d1e11ee420f15ef65811fea&sot=comb&sdt=cl&cluster=scoexactkeywords%2c%22Animal+Experiment%22%2ct%2bscolang%2c%22English%22%2ct&sl=1001&s=%28%28TITLE-ABS-KEY%28%22peptides%22%29+OR+TITLE-ABS-KEY%28%22antioxidant+peptides%22%29+OR+TITLE-ABS-KEY+%28%22antimicrobial+peptides%22%29+OR+TITLE-ABS-KEY%28%22angiogenic+peptides%22%29+OR+TITLE-ABS-KEY+%28%22animal+peptides%22%29+OR+TITLE-ABS-KEY%28%22natural+peptides%22%29+OR+TITLE-ABS-KEY%28%22bioactive+peptides%22%29+OR+TITLE-ABS-KEY%28%22biological+peptides%22%29+OR+TITLE-ABS-KEY%28%22isolated+peptides%22%29+OR+TITLE-ABS-KEY%28%22extracted+peptides%22%29%29%29+AND+%28%28TITLE-ABS-KEY%28%22wound+healing%22%29+OR+TITLE-ABS-KEY%28%22regeneration%22%29+OR+TITLE-ABS-KEY%28%22skin+repair%22%29+OR+TITLE-ABS-KEY%28%22cutaneous+repair%22%29+OR+TITLE-ABS-KEY%28%22skin+healing%22%29+OR+TITLE-ABS-KEY%28%22cutaneous+healing%22%29%29%29+AND+%28%28TITLE-ABS-KEY%28%22skin%22%29+OR+TITLE-ABS-KEY%28%22dermis%22%29+OR+TITLE-ABS-KEY+%28%22epidermis%22%29+OR+TITLE-ABS-KEY%28%22subcutaneous+tissue%22%29+OR+TITLE-ABS-KEY%28%22hypodermis%22%29+OR+TITLE-ABS-KEY%28%22granulation+tissue%22%29+OR+TITLE-ABS-KEY%28%22keratinocytes%22%29+OR+TITLE-ABS-KEY%28%22fibroblasts%22%29+OR+TITLE-ABS-KEY+%28%22integumentary+system%22%29+OR+TITLE-ABS-KEY%28%22skin+injuries%22%29+OR+TITLE-ABS-KEY%28%22skin+fibrosis%22%29+OR+TITLE-ABS-KEY%28%22skin+scars%22%29%29%29&relpos=313&citeCnt=73&searchTerm=) \| | 77 |
| Peptides of unreported origin |  | \| [Angiogenic laminin-derived peptides stimulate wound healing](https://www-scopus.ez35.periodicos.capes.gov.br/record/display.uri?eid=2-s2.0-51449111472&origin=resultslist&sort=plf-f&src=s&nlo=&nlr=&nls=&mltAll=t&sid=8f682cda9d1e11ee420f15ef65811fea&sot=comb&sdt=cl&cluster=scoexactkeywords%2c%22Animal+Experiment%22%2ct%2bscolang%2c%22English%22%2ct&sl=1001&s=%28%28TITLE-ABS-KEY%28%22peptides%22%29+OR+TITLE-ABS-KEY%28%22antioxidant+peptides%22%29+OR+TITLE-ABS-KEY+%28%22antimicrobial+peptides%22%29+OR+TITLE-ABS-KEY%28%22angiogenic+peptides%22%29+OR+TITLE-ABS-KEY+%28%22animal+peptides%22%29+OR+TITLE-ABS-KEY%28%22natural+peptides%22%29+OR+TITLE-ABS-KEY%28%22bioactive+peptides%22%29+OR+TITLE-ABS-KEY%28%22biological+peptides%22%29+OR+TITLE-ABS-KEY%28%22isolated+peptides%22%29+OR+TITLE-ABS-KEY%28%22extracted+peptides%22%29%29%29+AND+%28%28TITLE-ABS-KEY%28%22wound+healing%22%29+OR+TITLE-ABS-KEY%28%22regeneration%22%29+OR+TITLE-ABS-KEY%28%22skin+repair%22%29+OR+TITLE-ABS-KEY%28%22cutaneous+repair%22%29+OR+TITLE-ABS-KEY%28%22skin+healing%22%29+OR+TITLE-ABS-KEY%28%22cutaneous+healing%22%29%29%29+AND+%28%28TITLE-ABS-KEY%28%22skin%22%29+OR+TITLE-ABS-KEY%28%22dermis%22%29+OR+TITLE-ABS-KEY+%28%22epidermis%22%29+OR+TITLE-ABS-KEY%28%22subcutaneous+tissue%22%29+OR+TITLE-ABS-KEY%28%22hypodermis%22%29+OR+TITLE-ABS-KEY%28%22granulation+tissue%22%29+OR+TITLE-ABS-KEY%28%22keratinocytes%22%29+OR+TITLE-ABS-KEY%28%22fibroblasts%22%29+OR+TITLE-ABS-KEY+%28%22integumentary+system%22%29+OR+TITLE-ABS-KEY%28%22skin+injuries%22%29+OR+TITLE-ABS-KEY%28%22skin+fibrosis%22%29+OR+TITLE-ABS-KEY%28%22skin+scars%22%29%29%29&relpos=568&citeCnt=25&searchTerm=) \| \| --- \| \| [Antimicrobial endotoxin-neutralizing peptides promote keratinocyte migration via P2X7 receptor activation and accelerate wound healing in vivo](https://www2.scopus.com/record/display.uri?eid=2-s2.0-85050798185&origin=resultslist&sort=plf-f&src=s&nlo=&nlr=&nls=&mltAll=t&sid=4d2dea855575f6fbdb403e5ce008a978&sot=comb&sdt=cl&cluster=scoexactkeywords%2c%22Animal+Experiment%22%2ct%2bscolang%2c%22English%22%2ct&sl=1001&s=%28%28TITLE-ABS-KEY%28%22peptides%22%29+OR+TITLE-ABS-KEY%28%22antioxidant+peptides%22%29+OR+TITLE-ABS-KEY+%28%22antimicrobial+peptides%22%29+OR+TITLE-ABS-KEY%28%22angiogenic+peptides%22%29+OR+TITLE-ABS-KEY+%28%22animal+peptides%22%29+OR+TITLE-ABS-KEY%28%22natural+peptides%22%29+OR+TITLE-ABS-KEY%28%22bioactive+peptides%22%29+OR+TITLE-ABS-KEY%28%22biological+peptides%22%29+OR+TITLE-ABS-KEY%28%22isolated+peptides%22%29+OR+TITLE-ABS-KEY%28%22extracted+peptides%22%29%29%29+AND+%28%28TITLE-ABS-KEY%28%22wound+healing%22%29+OR+TITLE-ABS-KEY%28%22regeneration%22%29+OR+TITLE-ABS-KEY%28%22skin+repair%22%29+OR+TITLE-ABS-KEY%28%22cutaneous+repair%22%29+OR+TITLE-ABS-KEY%28%22skin+healing%22%29+OR+TITLE-ABS-KEY%28%22cutaneous+healing%22%29%29%29+AND+%28%28TITLE-ABS-KEY%28%22skin%22%29+OR+TITLE-ABS-KEY%28%22dermis%22%29+OR+TITLE-ABS-KEY+%28%22epidermis%22%29+OR+TITLE-ABS-KEY%28%22subcutaneous+tissue%22%29+OR+TITLE-ABS-KEY%28%22hypodermis%22%29+OR+TITLE-ABS-KEY%28%22granulation+tissue%22%29+OR+TITLE-ABS-KEY%28%22keratinocytes%22%29+OR+TITLE-ABS-KEY%28%22fibroblasts%22%29+OR+TITLE-ABS-KEY+%28%22integumentary+system%22%29+OR+TITLE-ABS-KEY%28%22skin+injuries%22%29+OR+TITLE-ABS-KEY%28%22skin+fibrosis%22%29+OR+TITLE-ABS-KEY%28%22skin+scars%22%29%29%29&relpos=53&citeCnt=3&searchTerm=) \| \| [Carnosine enhances diabetic wound healing in the db/db mouse model of type 2 diabetes](https://www-scopus.ez35.periodicos.capes.gov.br/record/display.uri?eid=2-s2.0-84862765718&origin=resultslist&sort=plf-f&src=s&nlo=&nlr=&nls=&mltAll=t&sid=8f682cda9d1e11ee420f15ef65811fea&sot=comb&sdt=cl&cluster=scoexactkeywords%2c%22Animal+Experiment%22%2ct%2bscolang%2c%22English%22%2ct&sl=1001&s=%28%28TITLE-ABS-KEY%28%22peptides%22%29+OR+TITLE-ABS-KEY%28%22antioxidant+peptides%22%29+OR+TITLE-ABS-KEY+%28%22antimicrobial+peptides%22%29+OR+TITLE-ABS-KEY%28%22angiogenic+peptides%22%29+OR+TITLE-ABS-KEY+%28%22animal+peptides%22%29+OR+TITLE-ABS-KEY%28%22natural+peptides%22%29+OR+TITLE-ABS-KEY%28%22bioactive+peptides%22%29+OR+TITLE-ABS-KEY%28%22biological+peptides%22%29+OR+TITLE-ABS-KEY%28%22isolated+peptides%22%29+OR+TITLE-ABS-KEY%28%22extracted+peptides%22%29%29%29+AND+%28%28TITLE-ABS-KEY%28%22wound+healing%22%29+OR+TITLE-ABS-KEY%28%22regeneration%22%29+OR+TITLE-ABS-KEY%28%22skin+repair%22%29+OR+TITLE-ABS-KEY%28%22cutaneous+repair%22%29+OR+TITLE-ABS-KEY%28%22skin+healing%22%29+OR+TITLE-ABS-KEY%28%22cutaneous+healing%22%29%29%29+AND+%28%28TITLE-ABS-KEY%28%22skin%22%29+OR+TITLE-ABS-KEY%28%22dermis%22%29+OR+TITLE-ABS-KEY+%28%22epidermis%22%29+OR+TITLE-ABS-KEY%28%22subcutaneous+tissue%22%29+OR+TITLE-ABS-KEY%28%22hypodermis%22%29+OR+TITLE-ABS-KEY%28%22granulation+tissue%22%29+OR+TITLE-ABS-KEY%28%22keratinocytes%22%29+OR+TITLE-ABS-KEY%28%22fibroblasts%22%29+OR+TITLE-ABS-KEY+%28%22integumentary+system%22%29+OR+TITLE-ABS-KEY%28%22skin+injuries%22%29+OR+TITLE-ABS-KEY%28%22skin+fibrosis%22%29+OR+TITLE-ABS-KEY%28%22skin+scars%22%29%29%29&relpos=402&citeCnt=40&searchTerm=) \| \| [Efficacy of the quorum sensing inhibitor FS10 alone and in combination with tigecycline in an animal model of staphylococcal infected wound](https://www-scopus.ez35.periodicos.capes.gov.br/record/display.uri?eid=2-s2.0-84973325034&origin=resultslist&sort=plf-f&src=s&nlo=&nlr=&nls=&mltAll=t&sid=8f682cda9d1e11ee420f15ef65811fea&sot=comb&sdt=cl&cluster=scoexactkeywords%2c%22Animal+Experiment%22%2ct%2bscolang%2c%22English%22%2ct&sl=1001&s=%28%28TITLE-ABS-KEY%28%22peptides%22%29+OR+TITLE-ABS-KEY%28%22antioxidant+peptides%22%29+OR+TITLE-ABS-KEY+%28%22antimicrobial+peptides%22%29+OR+TITLE-ABS-KEY%28%22angiogenic+peptides%22%29+OR+TITLE-ABS-KEY+%28%22animal+peptides%22%29+OR+TITLE-ABS-KEY%28%22natural+peptides%22%29+OR+TITLE-ABS-KEY%28%22bioactive+peptides%22%29+OR+TITLE-ABS-KEY%28%22biological+peptides%22%29+OR+TITLE-ABS-KEY%28%22isolated+peptides%22%29+OR+TITLE-ABS-KEY%28%22extracted+peptides%22%29%29%29+AND+%28%28TITLE-ABS-KEY%28%22wound+healing%22%29+OR+TITLE-ABS-KEY%28%22regeneration%22%29+OR+TITLE-ABS-KEY%28%22skin+repair%22%29+OR+TITLE-ABS-KEY%28%22cutaneous+repair%22%29+OR+TITLE-ABS-KEY%28%22skin+healing%22%29+OR+TITLE-ABS-KEY%28%22cutaneous+healing%22%29%29%29+AND+%28%28TITLE-ABS-KEY%28%22skin%22%29+OR+TITLE-ABS-KEY%28%22dermis%22%29+OR+TITLE-ABS-KEY+%28%22epidermis%22%29+OR+TITLE-ABS-KEY%28%22subcutaneous+tissue%22%29+OR+TITLE-ABS-KEY%28%22hypodermis%22%29+OR+TITLE-ABS-KEY%28%22granulation+tissue%22%29+OR+TITLE-ABS-KEY%28%22keratinocytes%22%29+OR+TITLE-ABS-KEY%28%22fibroblasts%22%29+OR+TITLE-ABS-KEY+%28%22integumentary+system%22%29+OR+TITLE-ABS-KEY%28%22skin+injuries%22%29+OR+TITLE-ABS-KEY%28%22skin+fibrosis%22%29+OR+TITLE-ABS-KEY%28%22skin+scars%22%29%29%29&relpos=191&citeCnt=17&searchTerm=) \| \| Egg White peptide KPHAEVVLR promotes skin fibroblasts migration and mice skin wound healing by stimulating cell membrane Hsp90 alpha secretion \| \| [Enhancement by PL 14736 of granulation and collagen organization in healing wounds and the potential role of egr-1 expression](https://www-scopus.ez35.periodicos.capes.gov.br/record/display.uri?eid=2-s2.0-34547837601&origin=resultslist&sort=plf-f&src=s&nlo=&nlr=&nls=&mltAll=t&sid=8f682cda9d1e11ee420f15ef65811fea&sot=comb&sdt=cl&cluster=scoexactkeywords%2c%22Animal+Experiment%22%2ct%2bscolang%2c%22English%22%2ct&sl=1001&s=%28%28TITLE-ABS-KEY%28%22peptides%22%29+OR+TITLE-ABS-KEY%28%22antioxidant+peptides%22%29+OR+TITLE-ABS-KEY+%28%22antimicrobial+peptides%22%29+OR+TITLE-ABS-KEY%28%22angiogenic+peptides%22%29+OR+TITLE-ABS-KEY+%28%22animal+peptides%22%29+OR+TITLE-ABS-KEY%28%22natural+peptides%22%29+OR+TITLE-ABS-KEY%28%22bioactive+peptides%22%29+OR+TITLE-ABS-KEY%28%22biological+peptides%22%29+OR+TITLE-ABS-KEY%28%22isolated+peptides%22%29+OR+TITLE-ABS-KEY%28%22extracted+peptides%22%29%29%29+AND+%28%28TITLE-ABS-KEY%28%22wound+healing%22%29+OR+TITLE-ABS-KEY%28%22regeneration%22%29+OR+TITLE-ABS-KEY%28%22skin+repair%22%29+OR+TITLE-ABS-KEY%28%22cutaneous+repair%22%29+OR+TITLE-ABS-KEY%28%22skin+healing%22%29+OR+TITLE-ABS-KEY%28%22cutaneous+healing%22%29%29%29+AND+%28%28TITLE-ABS-KEY%28%22skin%22%29+OR+TITLE-ABS-KEY%28%22dermis%22%29+OR+TITLE-ABS-KEY+%28%22epidermis%22%29+OR+TITLE-ABS-KEY%28%22subcutaneous+tissue%22%29+OR+TITLE-ABS-KEY%28%22hypodermis%22%29+OR+TITLE-ABS-KEY%28%22granulation+tissue%22%29+OR+TITLE-ABS-KEY%28%22keratinocytes%22%29+OR+TITLE-ABS-KEY%28%22fibroblasts%22%29+OR+TITLE-ABS-KEY+%28%22integumentary+system%22%29+OR+TITLE-ABS-KEY%28%22skin+injuries%22%29+OR+TITLE-ABS-KEY%28%22skin+fibrosis%22%29+OR+TITLE-ABS-KEY%28%22skin+scars%22%29%29%29&relpos=597&citeCnt=58&searchTerm=) \| \| [Exendin-4, a glucagon-like peptide-1 analogue, accelerates diabetic wound healing](https://www-scopus.ez35.periodicos.capes.gov.br/record/display.uri?eid=2-s2.0-84993981986&origin=resultslist&sort=plf-f&src=s&nlo=&nlr=&nls=&mltAll=t&sid=8f682cda9d1e11ee420f15ef65811fea&sot=comb&sdt=cl&cluster=scoexactkeywords%2c%22Animal+Experiment%22%2ct%2bscolang%2c%22English%22%2ct&sl=1001&s=%28%28TITLE-ABS-KEY%28%22peptides%22%29+OR+TITLE-ABS-KEY%28%22antioxidant+peptides%22%29+OR+TITLE-ABS-KEY+%28%22antimicrobial+peptides%22%29+OR+TITLE-ABS-KEY%28%22angiogenic+peptides%22%29+OR+TITLE-ABS-KEY+%28%22animal+peptides%22%29+OR+TITLE-ABS-KEY%28%22natural+peptides%22%29+OR+TITLE-ABS-KEY%28%22bioactive+peptides%22%29+OR+TITLE-ABS-KEY%28%22biological+peptides%22%29+OR+TITLE-ABS-KEY%28%22isolated+peptides%22%29+OR+TITLE-ABS-KEY%28%22extracted+peptides%22%29%29%29+AND+%28%28TITLE-ABS-KEY%28%22wound+healing%22%29+OR+TITLE-ABS-KEY%28%22regeneration%22%29+OR+TITLE-ABS-KEY%28%22skin+repair%22%29+OR+TITLE-ABS-KEY%28%22cutaneous+repair%22%29+OR+TITLE-ABS-KEY%28%22skin+healing%22%29+OR+TITLE-ABS-KEY%28%22cutaneous+healing%22%29%29%29+AND+%28%28TITLE-ABS-KEY%28%22skin%22%29+OR+TITLE-ABS-KEY%28%22dermis%22%29+OR+TITLE-ABS-KEY+%28%22epidermis%22%29+OR+TITLE-ABS-KEY%28%22subcutaneous+tissue%22%29+OR+TITLE-ABS-KEY%28%22hypodermis%22%29+OR+TITLE-ABS-KEY%28%22granulation+tissue%22%29+OR+TITLE-ABS-KEY%28%22keratinocytes%22%29+OR+TITLE-ABS-KEY%28%22fibroblasts%22%29+OR+TITLE-ABS-KEY+%28%22integumentary+system%22%29+OR+TITLE-ABS-KEY%28%22skin+injuries%22%29+OR+TITLE-ABS-KEY%28%22skin+fibrosis%22%29+OR+TITLE-ABS-KEY%28%22skin+scars%22%29%29%29&relpos=148&citeCnt=8&searchTerm=) \| \| [Glu-Trp-ONa or its acylated analogue (R-Glu-Trp-ONa) administration enhances the wound healing in the model of chronic skin wounds in rabbits](https://www-scopus.ez35.periodicos.capes.gov.br/record/display.uri?eid=2-s2.0-84929222889&origin=resultslist&sort=plf-f&src=s&nlo=&nlr=&nls=&mltAll=t&sid=8f682cda9d1e11ee420f15ef65811fea&sot=comb&sdt=cl&cluster=scoexactkeywords%2c%22Animal+Experiment%22%2ct%2bscolang%2c%22English%22%2ct&sl=1001&s=%28%28TITLE-ABS-KEY%28%22peptides%22%29+OR+TITLE-ABS-KEY%28%22antioxidant+peptides%22%29+OR+TITLE-ABS-KEY+%28%22antimicrobial+peptides%22%29+OR+TITLE-ABS-KEY%28%22angiogenic+peptides%22%29+OR+TITLE-ABS-KEY+%28%22animal+peptides%22%29+OR+TITLE-ABS-KEY%28%22natural+peptides%22%29+OR+TITLE-ABS-KEY%28%22bioactive+peptides%22%29+OR+TITLE-ABS-KEY%28%22biological+peptides%22%29+OR+TITLE-ABS-KEY%28%22isolated+peptides%22%29+OR+TITLE-ABS-KEY%28%22extracted+peptides%22%29%29%29+AND+%28%28TITLE-ABS-KEY%28%22wound+healing%22%29+OR+TITLE-ABS-KEY%28%22regeneration%22%29+OR+TITLE-ABS-KEY%28%22skin+repair%22%29+OR+TITLE-ABS-KEY%28%22cutaneous+repair%22%29+OR+TITLE-ABS-KEY%28%22skin+healing%22%29+OR+TITLE-ABS-KEY%28%22cutaneous+healing%22%29%29%29+AND+%28%28TITLE-ABS-KEY%28%22skin%22%29+OR+TITLE-ABS-KEY%28%22dermis%22%29+OR+TITLE-ABS-KEY+%28%22epidermis%22%29+OR+TITLE-ABS-KEY%28%22subcutaneous+tissue%22%29+OR+TITLE-ABS-KEY%28%22hypodermis%22%29+OR+TITLE-ABS-KEY%28%22granulation+tissue%22%29+OR+TITLE-ABS-KEY%28%22keratinocytes%22%29+OR+TITLE-ABS-KEY%28%22fibroblasts%22%29+OR+TITLE-ABS-KEY+%28%22integumentary+system%22%29+OR+TITLE-ABS-KEY%28%22skin+injuries%22%29+OR+TITLE-ABS-KEY%28%22skin+fibrosis%22%29+OR+TITLE-ABS-KEY%28%22skin+scars%22%29%29%29&relpos=253&citeCnt=2&searchTerm=) \| \| [Highly selective end-tagged antimicrobial peptides derived from PRELP](https://www.ncbi.nlm.nih.gov/pubmed/21298015) \| \| [Innate defense regulator peptide 1018 in wound healing and wound infection](https://www-scopus.ez35.periodicos.capes.gov.br/record/display.uri?eid=2-s2.0-84864717609&origin=resultslist&sort=plf-f&src=s&nlo=&nlr=&nls=&mltAll=t&sid=8f682cda9d1e11ee420f15ef65811fea&sot=comb&sdt=cl&cluster=scoexactkeywords%2c%22Animal+Experiment%22%2ct%2bscolang%2c%22English%22%2ct&sl=1001&s=%28%28TITLE-ABS-KEY%28%22peptides%22%29+OR+TITLE-ABS-KEY%28%22antioxidant+peptides%22%29+OR+TITLE-ABS-KEY+%28%22antimicrobial+peptides%22%29+OR+TITLE-ABS-KEY%28%22angiogenic+peptides%22%29+OR+TITLE-ABS-KEY+%28%22animal+peptides%22%29+OR+TITLE-ABS-KEY%28%22natural+peptides%22%29+OR+TITLE-ABS-KEY%28%22bioactive+peptides%22%29+OR+TITLE-ABS-KEY%28%22biological+peptides%22%29+OR+TITLE-ABS-KEY%28%22isolated+peptides%22%29+OR+TITLE-ABS-KEY%28%22extracted+peptides%22%29%29%29+AND+%28%28TITLE-ABS-KEY%28%22wound+healing%22%29+OR+TITLE-ABS-KEY%28%22regeneration%22%29+OR+TITLE-ABS-KEY%28%22skin+repair%22%29+OR+TITLE-ABS-KEY%28%22cutaneous+repair%22%29+OR+TITLE-ABS-KEY%28%22skin+healing%22%29+OR+TITLE-ABS-KEY%28%22cutaneous+healing%22%29%29%29+AND+%28%28TITLE-ABS-KEY%28%22skin%22%29+OR+TITLE-ABS-KEY%28%22dermis%22%29+OR+TITLE-ABS-KEY+%28%22epidermis%22%29+OR+TITLE-ABS-KEY%28%22subcutaneous+tissue%22%29+OR+TITLE-ABS-KEY%28%22hypodermis%22%29+OR+TITLE-ABS-KEY%28%22granulation+tissue%22%29+OR+TITLE-ABS-KEY%28%22keratinocytes%22%29+OR+TITLE-ABS-KEY%28%22fibroblasts%22%29+OR+TITLE-ABS-KEY+%28%22integumentary+system%22%29+OR+TITLE-ABS-KEY%28%22skin+injuries%22%29+OR+TITLE-ABS-KEY%28%22skin+fibrosis%22%29+OR+TITLE-ABS-KEY%28%22skin+scars%22%29%29%29&relpos=395&citeCnt=67&searchTerm=) \| \| [The effects of brain natriuretic peptide on scar formation in incisional rat wounds](https://www-scopus.ez35.periodicos.capes.gov.br/record/display.uri?eid=2-s2.0-56849098825&origin=resultslist&sort=plf-f&src=s&nlo=&nlr=&nls=&mltAll=t&sid=8f682cda9d1e11ee420f15ef65811fea&sot=comb&sdt=cl&cluster=scoexactkeywords%2c%22Animal+Experiment%22%2ct%2bscolang%2c%22English%22%2ct&sl=1001&s=%28%28TITLE-ABS-KEY%28%22peptides%22%29+OR+TITLE-ABS-KEY%28%22antioxidant+peptides%22%29+OR+TITLE-ABS-KEY+%28%22antimicrobial+peptides%22%29+OR+TITLE-ABS-KEY%28%22angiogenic+peptides%22%29+OR+TITLE-ABS-KEY+%28%22animal+peptides%22%29+OR+TITLE-ABS-KEY%28%22natural+peptides%22%29+OR+TITLE-ABS-KEY%28%22bioactive+peptides%22%29+OR+TITLE-ABS-KEY%28%22biological+peptides%22%29+OR+TITLE-ABS-KEY%28%22isolated+peptides%22%29+OR+TITLE-ABS-KEY%28%22extracted+peptides%22%29%29%29+AND+%28%28TITLE-ABS-KEY%28%22wound+healing%22%29+OR+TITLE-ABS-KEY%28%22regeneration%22%29+OR+TITLE-ABS-KEY%28%22skin+repair%22%29+OR+TITLE-ABS-KEY%28%22cutaneous+repair%22%29+OR+TITLE-ABS-KEY%28%22skin+healing%22%29+OR+TITLE-ABS-KEY%28%22cutaneous+healing%22%29%29%29+AND+%28%28TITLE-ABS-KEY%28%22skin%22%29+OR+TITLE-ABS-KEY%28%22dermis%22%29+OR+TITLE-ABS-KEY+%28%22epidermis%22%29+OR+TITLE-ABS-KEY%28%22subcutaneous+tissue%22%29+OR+TITLE-ABS-KEY%28%22hypodermis%22%29+OR+TITLE-ABS-KEY%28%22granulation+tissue%22%29+OR+TITLE-ABS-KEY%28%22keratinocytes%22%29+OR+TITLE-ABS-KEY%28%22fibroblasts%22%29+OR+TITLE-ABS-KEY+%28%22integumentary+system%22%29+OR+TITLE-ABS-KEY%28%22skin+injuries%22%29+OR+TITLE-ABS-KEY%28%22skin+fibrosis%22%29+OR+TITLE-ABS-KEY%28%22skin+scars%22%29%29%29&relpos=555&citeCnt=0&searchTerm=) \| \| [The pro-healing effect of exendin-4 on wounds produced by abrasion in normoglycemic mice](https://www-scopus.ez35.periodicos.capes.gov.br/record/display.uri?eid=2-s2.0-84937458617&origin=resultslist&sort=plf-f&src=s&nlo=&nlr=&nls=&mltAll=t&sid=8f682cda9d1e11ee420f15ef65811fea&sot=comb&sdt=cl&cluster=scoexactkeywords%2c%22Animal+Experiment%22%2ct%2bscolang%2c%22English%22%2ct&sl=1001&s=%28%28TITLE-ABS-KEY%28%22peptides%22%29+OR+TITLE-ABS-KEY%28%22antioxidant+peptides%22%29+OR+TITLE-ABS-KEY+%28%22antimicrobial+peptides%22%29+OR+TITLE-ABS-KEY%28%22angiogenic+peptides%22%29+OR+TITLE-ABS-KEY+%28%22animal+peptides%22%29+OR+TITLE-ABS-KEY%28%22natural+peptides%22%29+OR+TITLE-ABS-KEY%28%22bioactive+peptides%22%29+OR+TITLE-ABS-KEY%28%22biological+peptides%22%29+OR+TITLE-ABS-KEY%28%22isolated+peptides%22%29+OR+TITLE-ABS-KEY%28%22extracted+peptides%22%29%29%29+AND+%28%28TITLE-ABS-KEY%28%22wound+healing%22%29+OR+TITLE-ABS-KEY%28%22regeneration%22%29+OR+TITLE-ABS-KEY%28%22skin+repair%22%29+OR+TITLE-ABS-KEY%28%22cutaneous+repair%22%29+OR+TITLE-ABS-KEY%28%22skin+healing%22%29+OR+TITLE-ABS-KEY%28%22cutaneous+healing%22%29%29%29+AND+%28%28TITLE-ABS-KEY%28%22skin%22%29+OR+TITLE-ABS-KEY%28%22dermis%22%29+OR+TITLE-ABS-KEY+%28%22epidermis%22%29+OR+TITLE-ABS-KEY%28%22subcutaneous+tissue%22%29+OR+TITLE-ABS-KEY%28%22hypodermis%22%29+OR+TITLE-ABS-KEY%28%22granulation+tissue%22%29+OR+TITLE-ABS-KEY%28%22keratinocytes%22%29+OR+TITLE-ABS-KEY%28%22fibroblasts%22%29+OR+TITLE-ABS-KEY+%28%22integumentary+system%22%29+OR+TITLE-ABS-KEY%28%22skin+injuries%22%29+OR+TITLE-ABS-KEY%28%22skin+fibrosis%22%29+OR+TITLE-ABS-KEY%28%22skin+scars%22%29%29%29&relpos=239&citeCnt=7&searchTerm=) \| \| [Variants of self-assembling peptide, KLD-12 that show both rapid fracture healing and antimicrobial properties](https://www.ncbi.nlm.nih.gov/pubmed/25934283) \| | 13 |
| Non-animal peptides |  | \| [A peptide inhibitor of c-Jun promotes wound healing in a mouse full-thickness burn model](https://www-scopus.ez35.periodicos.capes.gov.br/record/display.uri?eid=2-s2.0-38349170919&origin=resultslist&sort=plf-f&src=s&nlo=&nlr=&nls=&mltAll=t&sid=8f682cda9d1e11ee420f15ef65811fea&sot=comb&sdt=cl&cluster=scoexactkeywords%2c%22Animal+Experiment%22%2ct%2bscolang%2c%22English%22%2ct&sl=1001&s=%28%28TITLE-ABS-KEY%28%22peptides%22%29+OR+TITLE-ABS-KEY%28%22antioxidant+peptides%22%29+OR+TITLE-ABS-KEY+%28%22antimicrobial+peptides%22%29+OR+TITLE-ABS-KEY%28%22angiogenic+peptides%22%29+OR+TITLE-ABS-KEY+%28%22animal+peptides%22%29+OR+TITLE-ABS-KEY%28%22natural+peptides%22%29+OR+TITLE-ABS-KEY%28%22bioactive+peptides%22%29+OR+TITLE-ABS-KEY%28%22biological+peptides%22%29+OR+TITLE-ABS-KEY%28%22isolated+peptides%22%29+OR+TITLE-ABS-KEY%28%22extracted+peptides%22%29%29%29+AND+%28%28TITLE-ABS-KEY%28%22wound+healing%22%29+OR+TITLE-ABS-KEY%28%22regeneration%22%29+OR+TITLE-ABS-KEY%28%22skin+repair%22%29+OR+TITLE-ABS-KEY%28%22cutaneous+repair%22%29+OR+TITLE-ABS-KEY%28%22skin+healing%22%29+OR+TITLE-ABS-KEY%28%22cutaneous+healing%22%29%29%29+AND+%28%28TITLE-ABS-KEY%28%22skin%22%29+OR+TITLE-ABS-KEY%28%22dermis%22%29+OR+TITLE-ABS-KEY+%28%22epidermis%22%29+OR+TITLE-ABS-KEY%28%22subcutaneous+tissue%22%29+OR+TITLE-ABS-KEY%28%22hypodermis%22%29+OR+TITLE-ABS-KEY%28%22granulation+tissue%22%29+OR+TITLE-ABS-KEY%28%22keratinocytes%22%29+OR+TITLE-ABS-KEY%28%22fibroblasts%22%29+OR+TITLE-ABS-KEY+%28%22integumentary+system%22%29+OR+TITLE-ABS-KEY%28%22skin+injuries%22%29+OR+TITLE-ABS-KEY%28%22skin+fibrosis%22%29+OR+TITLE-ABS-KEY%28%22skin+scars%22%29%29%29&relpos=585&citeCnt=17&searchTerm=) \| \| --- \| \| [A RHAMM mimetic peptide blocks hyaluronan signaling and reduces inflammation and fibrogenesis in excisional skin wounds](https://www-scopus.ez35.periodicos.capes.gov.br/record/display.uri?eid=2-s2.0-84866504259&origin=resultslist&sort=plf-f&src=s&nlo=&nlr=&nls=&mltAll=t&sid=8f682cda9d1e11ee420f15ef65811fea&sot=comb&sdt=cl&cluster=scoexactkeywords%2c%22Animal+Experiment%22%2ct%2bscolang%2c%22English%22%2ct&sl=1001&s=%28%28TITLE-ABS-KEY%28%22peptides%22%29+OR+TITLE-ABS-KEY%28%22antioxidant+peptides%22%29+OR+TITLE-ABS-KEY+%28%22antimicrobial+peptides%22%29+OR+TITLE-ABS-KEY%28%22angiogenic+peptides%22%29+OR+TITLE-ABS-KEY+%28%22animal+peptides%22%29+OR+TITLE-ABS-KEY%28%22natural+peptides%22%29+OR+TITLE-ABS-KEY%28%22bioactive+peptides%22%29+OR+TITLE-ABS-KEY%28%22biological+peptides%22%29+OR+TITLE-ABS-KEY%28%22isolated+peptides%22%29+OR+TITLE-ABS-KEY%28%22extracted+peptides%22%29%29%29+AND+%28%28TITLE-ABS-KEY%28%22wound+healing%22%29+OR+TITLE-ABS-KEY%28%22regeneration%22%29+OR+TITLE-ABS-KEY%28%22skin+repair%22%29+OR+TITLE-ABS-KEY%28%22cutaneous+repair%22%29+OR+TITLE-ABS-KEY%28%22skin+healing%22%29+OR+TITLE-ABS-KEY%28%22cutaneous+healing%22%29%29%29+AND+%28%28TITLE-ABS-KEY%28%22skin%22%29+OR+TITLE-ABS-KEY%28%22dermis%22%29+OR+TITLE-ABS-KEY+%28%22epidermis%22%29+OR+TITLE-ABS-KEY%28%22subcutaneous+tissue%22%29+OR+TITLE-ABS-KEY%28%22hypodermis%22%29+OR+TITLE-ABS-KEY%28%22granulation+tissue%22%29+OR+TITLE-ABS-KEY%28%22keratinocytes%22%29+OR+TITLE-ABS-KEY%28%22fibroblasts%22%29+OR+TITLE-ABS-KEY+%28%22integumentary+system%22%29+OR+TITLE-ABS-KEY%28%22skin+injuries%22%29+OR+TITLE-ABS-KEY%28%22skin+fibrosis%22%29+OR+TITLE-ABS-KEY%28%22skin+scars%22%29%29%29&relpos=390&citeCnt=56&searchTerm=) \| \| [A small peptide with potential ability to promote wound healing](https://www-scopus.ez35.periodicos.capes.gov.br/record/display.uri?eid=2-s2.0-84898655740&origin=resultslist&sort=plf-f&src=s&nlo=&nlr=&nls=&mltAll=t&sid=8f682cda9d1e11ee420f15ef65811fea&sot=comb&sdt=cl&cluster=scoexactkeywords%2c%22Animal+Experiment%22%2ct%2bscolang%2c%22English%22%2ct&sl=1001&s=%28%28TITLE-ABS-KEY%28%22peptides%22%29+OR+TITLE-ABS-KEY%28%22antioxidant+peptides%22%29+OR+TITLE-ABS-KEY+%28%22antimicrobial+peptides%22%29+OR+TITLE-ABS-KEY%28%22angiogenic+peptides%22%29+OR+TITLE-ABS-KEY+%28%22animal+peptides%22%29+OR+TITLE-ABS-KEY%28%22natural+peptides%22%29+OR+TITLE-ABS-KEY%28%22bioactive+peptides%22%29+OR+TITLE-ABS-KEY%28%22biological+peptides%22%29+OR+TITLE-ABS-KEY%28%22isolated+peptides%22%29+OR+TITLE-ABS-KEY%28%22extracted+peptides%22%29%29%29+AND+%28%28TITLE-ABS-KEY%28%22wound+healing%22%29+OR+TITLE-ABS-KEY%28%22regeneration%22%29+OR+TITLE-ABS-KEY%28%22skin+repair%22%29+OR+TITLE-ABS-KEY%28%22cutaneous+repair%22%29+OR+TITLE-ABS-KEY%28%22skin+healing%22%29+OR+TITLE-ABS-KEY%28%22cutaneous+healing%22%29%29%29+AND+%28%28TITLE-ABS-KEY%28%22skin%22%29+OR+TITLE-ABS-KEY%28%22dermis%22%29+OR+TITLE-ABS-KEY+%28%22epidermis%22%29+OR+TITLE-ABS-KEY%28%22subcutaneous+tissue%22%29+OR+TITLE-ABS-KEY%28%22hypodermis%22%29+OR+TITLE-ABS-KEY%28%22granulation+tissue%22%29+OR+TITLE-ABS-KEY%28%22keratinocytes%22%29+OR+TITLE-ABS-KEY%28%22fibroblasts%22%29+OR+TITLE-ABS-KEY+%28%22integumentary+system%22%29+OR+TITLE-ABS-KEY%28%22skin+injuries%22%29+OR+TITLE-ABS-KEY%28%22skin+fibrosis%22%29+OR+TITLE-ABS-KEY%28%22skin+scars%22%29%29%29&relpos=302&citeCnt=32&searchTerm=) \| \| [Discovery and characterization of a high-affinity small peptide ligand, H1, targeting FGFR2IIIc for Skin Wound Healing](https://www2.scopus.com/record/display.uri?eid=2-s2.0-85053673364&origin=resultslist&sort=plf-f&src=s&nlo=&nlr=&nls=&mltAll=t&sid=4d2dea855575f6fbdb403e5ce008a978&sot=comb&sdt=cl&cluster=scoexactkeywords%2c%22Animal+Experiment%22%2ct%2bscolang%2c%22English%22%2ct&sl=1001&s=%28%28TITLE-ABS-KEY%28%22peptides%22%29+OR+TITLE-ABS-KEY%28%22antioxidant+peptides%22%29+OR+TITLE-ABS-KEY+%28%22antimicrobial+peptides%22%29+OR+TITLE-ABS-KEY%28%22angiogenic+peptides%22%29+OR+TITLE-ABS-KEY+%28%22animal+peptides%22%29+OR+TITLE-ABS-KEY%28%22natural+peptides%22%29+OR+TITLE-ABS-KEY%28%22bioactive+peptides%22%29+OR+TITLE-ABS-KEY%28%22biological+peptides%22%29+OR+TITLE-ABS-KEY%28%22isolated+peptides%22%29+OR+TITLE-ABS-KEY%28%22extracted+peptides%22%29%29%29+AND+%28%28TITLE-ABS-KEY%28%22wound+healing%22%29+OR+TITLE-ABS-KEY%28%22regeneration%22%29+OR+TITLE-ABS-KEY%28%22skin+repair%22%29+OR+TITLE-ABS-KEY%28%22cutaneous+repair%22%29+OR+TITLE-ABS-KEY%28%22skin+healing%22%29+OR+TITLE-ABS-KEY%28%22cutaneous+healing%22%29%29%29+AND+%28%28TITLE-ABS-KEY%28%22skin%22%29+OR+TITLE-ABS-KEY%28%22dermis%22%29+OR+TITLE-ABS-KEY+%28%22epidermis%22%29+OR+TITLE-ABS-KEY%28%22subcutaneous+tissue%22%29+OR+TITLE-ABS-KEY%28%22hypodermis%22%29+OR+TITLE-ABS-KEY%28%22granulation+tissue%22%29+OR+TITLE-ABS-KEY%28%22keratinocytes%22%29+OR+TITLE-ABS-KEY%28%22fibroblasts%22%29+OR+TITLE-ABS-KEY+%28%22integumentary+system%22%29+OR+TITLE-ABS-KEY%28%22skin+injuries%22%29+OR+TITLE-ABS-KEY%28%22skin+fibrosis%22%29+OR+TITLE-ABS-KEY%28%22skin+scars%22%29%29%29&relpos=38&citeCnt=1&searchTerm=) \| \| [Effect of tripeptide-copper complexes on the process of skin wound healing and on cultured fibroblasts.](https://www.ncbi.nlm.nih.gov/pubmed/8836453) \| \| [Efficacy of the designer antimicrobial peptide SHAP1 in wound healing and wound infection.](https://www.ncbi.nlm.nih.gov/pubmed/24952727) \| \| [Myxinidin2 and myxinidin3 suppress inflammatory responses through STAT3 and MAPKs to promote wound healing](https://www-scopus.ez35.periodicos.capes.gov.br/record/display.uri?eid=2-s2.0-85031719985&origin=resultslist&sort=plf-f&src=s&nlo=&nlr=&nls=&mltAll=t&sid=8f682cda9d1e11ee420f15ef65811fea&sot=comb&sdt=cl&cluster=scoexactkeywords%2c%22Animal+Experiment%22%2ct%2bscolang%2c%22English%22%2ct&sl=1001&s=%28%28TITLE-ABS-KEY%28%22peptides%22%29+OR+TITLE-ABS-KEY%28%22antioxidant+peptides%22%29+OR+TITLE-ABS-KEY+%28%22antimicrobial+peptides%22%29+OR+TITLE-ABS-KEY%28%22angiogenic+peptides%22%29+OR+TITLE-ABS-KEY+%28%22animal+peptides%22%29+OR+TITLE-ABS-KEY%28%22natural+peptides%22%29+OR+TITLE-ABS-KEY%28%22bioactive+peptides%22%29+OR+TITLE-ABS-KEY%28%22biological+peptides%22%29+OR+TITLE-ABS-KEY%28%22isolated+peptides%22%29+OR+TITLE-ABS-KEY%28%22extracted+peptides%22%29%29%29+AND+%28%28TITLE-ABS-KEY%28%22wound+healing%22%29+OR+TITLE-ABS-KEY%28%22regeneration%22%29+OR+TITLE-ABS-KEY%28%22skin+repair%22%29+OR+TITLE-ABS-KEY%28%22cutaneous+repair%22%29+OR+TITLE-ABS-KEY%28%22skin+healing%22%29+OR+TITLE-ABS-KEY%28%22cutaneous+healing%22%29%29%29+AND+%28%28TITLE-ABS-KEY%28%22skin%22%29+OR+TITLE-ABS-KEY%28%22dermis%22%29+OR+TITLE-ABS-KEY+%28%22epidermis%22%29+OR+TITLE-ABS-KEY%28%22subcutaneous+tissue%22%29+OR+TITLE-ABS-KEY%28%22hypodermis%22%29+OR+TITLE-ABS-KEY%28%22granulation+tissue%22%29+OR+TITLE-ABS-KEY%28%22keratinocytes%22%29+OR+TITLE-ABS-KEY%28%22fibroblasts%22%29+OR+TITLE-ABS-KEY+%28%22integumentary+system%22%29+OR+TITLE-ABS-KEY%28%22skin+injuries%22%29+OR+TITLE-ABS-KEY%28%22skin+fibrosis%22%29+OR+TITLE-ABS-KEY%28%22skin+scars%22%29%29%29&relpos=151&citeCnt=3&searchTerm=) \| \| [Topical application of Sadat-Habdan mesenchymal stimulating peptide (SHMSP) accelerates wound healing in diabetic rabbits](https://www-scopus.ez35.periodicos.capes.gov.br/record/display.uri?eid=2-s2.0-84863700201&origin=resultslist&sort=plf-f&src=s&nlo=&nlr=&nls=&mltAll=t&sid=8f682cda9d1e11ee420f15ef65811fea&sot=comb&sdt=cl&cluster=scoexactkeywords%2c%22Animal+Experiment%22%2ct%2bscolang%2c%22English%22%2ct&sl=1001&s=%28%28TITLE-ABS-KEY%28%22peptides%22%29+OR+TITLE-ABS-KEY%28%22antioxidant+peptides%22%29+OR+TITLE-ABS-KEY+%28%22antimicrobial+peptides%22%29+OR+TITLE-ABS-KEY%28%22angiogenic+peptides%22%29+OR+TITLE-ABS-KEY+%28%22animal+peptides%22%29+OR+TITLE-ABS-KEY%28%22natural+peptides%22%29+OR+TITLE-ABS-KEY%28%22bioactive+peptides%22%29+OR+TITLE-ABS-KEY%28%22biological+peptides%22%29+OR+TITLE-ABS-KEY%28%22isolated+peptides%22%29+OR+TITLE-ABS-KEY%28%22extracted+peptides%22%29%29%29+AND+%28%28TITLE-ABS-KEY%28%22wound+healing%22%29+OR+TITLE-ABS-KEY%28%22regeneration%22%29+OR+TITLE-ABS-KEY%28%22skin+repair%22%29+OR+TITLE-ABS-KEY%28%22cutaneous+repair%22%29+OR+TITLE-ABS-KEY%28%22skin+healing%22%29+OR+TITLE-ABS-KEY%28%22cutaneous+healing%22%29%29%29+AND+%28%28TITLE-ABS-KEY%28%22skin%22%29+OR+TITLE-ABS-KEY%28%22dermis%22%29+OR+TITLE-ABS-KEY+%28%22epidermis%22%29+OR+TITLE-ABS-KEY%28%22subcutaneous+tissue%22%29+OR+TITLE-ABS-KEY%28%22hypodermis%22%29+OR+TITLE-ABS-KEY%28%22granulation+tissue%22%29+OR+TITLE-ABS-KEY%28%22keratinocytes%22%29+OR+TITLE-ABS-KEY%28%22fibroblasts%22%29+OR+TITLE-ABS-KEY+%28%22integumentary+system%22%29+OR+TITLE-ABS-KEY%28%22skin+injuries%22%29+OR+TITLE-ABS-KEY%28%22skin+fibrosis%22%29+OR+TITLE-ABS-KEY%28%22skin+scars%22%29%29%29&relpos=398&citeCnt=3&searchTerm=) \| \| [Wound healing acceleration of a novel transforming growth factor-β inducer, SEK-1005](https://www-scopus.ez35.periodicos.capes.gov.br/record/display.uri?eid=2-s2.0-0034680843&origin=resultslist&sort=plf-f&src=s&nlo=&nlr=&nls=&mltAll=t&sid=8f682cda9d1e11ee420f15ef65811fea&sot=comb&sdt=cl&cluster=scoexactkeywords%2c%22Animal+Experiment%22%2ct%2bscolang%2c%22English%22%2ct&sl=1001&s=%28%28TITLE-ABS-KEY%28%22peptides%22%29+OR+TITLE-ABS-KEY%28%22antioxidant+peptides%22%29+OR+TITLE-ABS-KEY+%28%22antimicrobial+peptides%22%29+OR+TITLE-ABS-KEY%28%22angiogenic+peptides%22%29+OR+TITLE-ABS-KEY+%28%22animal+peptides%22%29+OR+TITLE-ABS-KEY%28%22natural+peptides%22%29+OR+TITLE-ABS-KEY%28%22bioactive+peptides%22%29+OR+TITLE-ABS-KEY%28%22biological+peptides%22%29+OR+TITLE-ABS-KEY%28%22isolated+peptides%22%29+OR+TITLE-ABS-KEY%28%22extracted+peptides%22%29%29%29+AND+%28%28TITLE-ABS-KEY%28%22wound+healing%22%29+OR+TITLE-ABS-KEY%28%22regeneration%22%29+OR+TITLE-ABS-KEY%28%22skin+repair%22%29+OR+TITLE-ABS-KEY%28%22cutaneous+repair%22%29+OR+TITLE-ABS-KEY%28%22skin+healing%22%29+OR+TITLE-ABS-KEY%28%22cutaneous+healing%22%29%29%29+AND+%28%28TITLE-ABS-KEY%28%22skin%22%29+OR+TITLE-ABS-KEY%28%22dermis%22%29+OR+TITLE-ABS-KEY+%28%22epidermis%22%29+OR+TITLE-ABS-KEY%28%22subcutaneous+tissue%22%29+OR+TITLE-ABS-KEY%28%22hypodermis%22%29+OR+TITLE-ABS-KEY%28%22granulation+tissue%22%29+OR+TITLE-ABS-KEY%28%22keratinocytes%22%29+OR+TITLE-ABS-KEY%28%22fibroblasts%22%29+OR+TITLE-ABS-KEY+%28%22integumentary+system%22%29+OR+TITLE-ABS-KEY%28%22skin+injuries%22%29+OR+TITLE-ABS-KEY%28%22skin+fibrosis%22%29+OR+TITLE-ABS-KEY%28%22skin+scars%22%29%29%29&relpos=735&citeCnt=14&searchTerm=) \| | 9 |
| Sutured wounds |  | \| [Effect of NorLeu3-A(1-7) on scar formation over time after full-thickness incision injury in the rat](https://www-scopus.ez35.periodicos.capes.gov.br/record/display.uri?eid=2-s2.0-20544477456&origin=resultslist&sort=plf-f&src=s&nlo=&nlr=&nls=&mltAll=t&sid=8f682cda9d1e11ee420f15ef65811fea&sot=comb&sdt=cl&cluster=scoexactkeywords%2c%22Animal+Experiment%22%2ct%2bscolang%2c%22English%22%2ct&sl=1001&s=%28%28TITLE-ABS-KEY%28%22peptides%22%29+OR+TITLE-ABS-KEY%28%22antioxidant+peptides%22%29+OR+TITLE-ABS-KEY+%28%22antimicrobial+peptides%22%29+OR+TITLE-ABS-KEY%28%22angiogenic+peptides%22%29+OR+TITLE-ABS-KEY+%28%22animal+peptides%22%29+OR+TITLE-ABS-KEY%28%22natural+peptides%22%29+OR+TITLE-ABS-KEY%28%22bioactive+peptides%22%29+OR+TITLE-ABS-KEY%28%22biological+peptides%22%29+OR+TITLE-ABS-KEY%28%22isolated+peptides%22%29+OR+TITLE-ABS-KEY%28%22extracted+peptides%22%29%29%29+AND+%28%28TITLE-ABS-KEY%28%22wound+healing%22%29+OR+TITLE-ABS-KEY%28%22regeneration%22%29+OR+TITLE-ABS-KEY%28%22skin+repair%22%29+OR+TITLE-ABS-KEY%28%22cutaneous+repair%22%29+OR+TITLE-ABS-KEY%28%22skin+healing%22%29+OR+TITLE-ABS-KEY%28%22cutaneous+healing%22%29%29%29+AND+%28%28TITLE-ABS-KEY%28%22skin%22%29+OR+TITLE-ABS-KEY%28%22dermis%22%29+OR+TITLE-ABS-KEY+%28%22epidermis%22%29+OR+TITLE-ABS-KEY%28%22subcutaneous+tissue%22%29+OR+TITLE-ABS-KEY%28%22hypodermis%22%29+OR+TITLE-ABS-KEY%28%22granulation+tissue%22%29+OR+TITLE-ABS-KEY%28%22keratinocytes%22%29+OR+TITLE-ABS-KEY%28%22fibroblasts%22%29+OR+TITLE-ABS-KEY+%28%22integumentary+system%22%29+OR+TITLE-ABS-KEY%28%22skin+injuries%22%29+OR+TITLE-ABS-KEY%28%22skin+fibrosis%22%29+OR+TITLE-ABS-KEY%28%22skin+scars%22%29%29%29&relpos=648&citeCnt=7&searchTerm=) \| \| --- \| \| [Exogenously-administered Leptin increases early incisional wound angiogenesis in an experimental animal model](https://www-scopus.ez35.periodicos.capes.gov.br/record/display.uri?eid=2-s2.0-35649014253&origin=resultslist&sort=plf-f&src=s&nlo=&nlr=&nls=&mltAll=t&sid=8f682cda9d1e11ee420f15ef65811fea&sot=comb&sdt=cl&cluster=scoexactkeywords%2c%22Animal+Experiment%22%2ct%2bscolang%2c%22English%22%2ct&sl=1001&s=%28%28TITLE-ABS-KEY%28%22peptides%22%29+OR+TITLE-ABS-KEY%28%22antioxidant+peptides%22%29+OR+TITLE-ABS-KEY+%28%22antimicrobial+peptides%22%29+OR+TITLE-ABS-KEY%28%22angiogenic+peptides%22%29+OR+TITLE-ABS-KEY+%28%22animal+peptides%22%29+OR+TITLE-ABS-KEY%28%22natural+peptides%22%29+OR+TITLE-ABS-KEY%28%22bioactive+peptides%22%29+OR+TITLE-ABS-KEY%28%22biological+peptides%22%29+OR+TITLE-ABS-KEY%28%22isolated+peptides%22%29+OR+TITLE-ABS-KEY%28%22extracted+peptides%22%29%29%29+AND+%28%28TITLE-ABS-KEY%28%22wound+healing%22%29+OR+TITLE-ABS-KEY%28%22regeneration%22%29+OR+TITLE-ABS-KEY%28%22skin+repair%22%29+OR+TITLE-ABS-KEY%28%22cutaneous+repair%22%29+OR+TITLE-ABS-KEY%28%22skin+healing%22%29+OR+TITLE-ABS-KEY%28%22cutaneous+healing%22%29%29%29+AND+%28%28TITLE-ABS-KEY%28%22skin%22%29+OR+TITLE-ABS-KEY%28%22dermis%22%29+OR+TITLE-ABS-KEY+%28%22epidermis%22%29+OR+TITLE-ABS-KEY%28%22subcutaneous+tissue%22%29+OR+TITLE-ABS-KEY%28%22hypodermis%22%29+OR+TITLE-ABS-KEY%28%22granulation+tissue%22%29+OR+TITLE-ABS-KEY%28%22keratinocytes%22%29+OR+TITLE-ABS-KEY%28%22fibroblasts%22%29+OR+TITLE-ABS-KEY+%28%22integumentary+system%22%29+OR+TITLE-ABS-KEY%28%22skin+injuries%22%29+OR+TITLE-ABS-KEY%28%22skin+fibrosis%22%29+OR+TITLE-ABS-KEY%28%22skin+scars%22%29%29%29&relpos=602&citeCnt=11&searchTerm=) \| \| [Impact of single-dose application of TGF-β, copper peptide, stanozolol and ascorbic acid in hydrogel on midline laparatomy wound healing in a diabetic mouse model](https://www-scopus.ez35.periodicos.capes.gov.br/record/display.uri?eid=2-s2.0-84863544443&origin=resultslist&sort=plf-f&src=s&nlo=&nlr=&nls=&mltAll=t&sid=8f682cda9d1e11ee420f15ef65811fea&sot=comb&sdt=cl&cluster=scoexactkeywords%2c%22Animal+Experiment%22%2ct%2bscolang%2c%22English%22%2ct&sl=1001&s=%28%28TITLE-ABS-KEY%28%22peptides%22%29+OR+TITLE-ABS-KEY%28%22antioxidant+peptides%22%29+OR+TITLE-ABS-KEY+%28%22antimicrobial+peptides%22%29+OR+TITLE-ABS-KEY%28%22angiogenic+peptides%22%29+OR+TITLE-ABS-KEY+%28%22animal+peptides%22%29+OR+TITLE-ABS-KEY%28%22natural+peptides%22%29+OR+TITLE-ABS-KEY%28%22bioactive+peptides%22%29+OR+TITLE-ABS-KEY%28%22biological+peptides%22%29+OR+TITLE-ABS-KEY%28%22isolated+peptides%22%29+OR+TITLE-ABS-KEY%28%22extracted+peptides%22%29%29%29+AND+%28%28TITLE-ABS-KEY%28%22wound+healing%22%29+OR+TITLE-ABS-KEY%28%22regeneration%22%29+OR+TITLE-ABS-KEY%28%22skin+repair%22%29+OR+TITLE-ABS-KEY%28%22cutaneous+repair%22%29+OR+TITLE-ABS-KEY%28%22skin+healing%22%29+OR+TITLE-ABS-KEY%28%22cutaneous+healing%22%29%29%29+AND+%28%28TITLE-ABS-KEY%28%22skin%22%29+OR+TITLE-ABS-KEY%28%22dermis%22%29+OR+TITLE-ABS-KEY+%28%22epidermis%22%29+OR+TITLE-ABS-KEY%28%22subcutaneous+tissue%22%29+OR+TITLE-ABS-KEY%28%22hypodermis%22%29+OR+TITLE-ABS-KEY%28%22granulation+tissue%22%29+OR+TITLE-ABS-KEY%28%22keratinocytes%22%29+OR+TITLE-ABS-KEY%28%22fibroblasts%22%29+OR+TITLE-ABS-KEY+%28%22integumentary+system%22%29+OR+TITLE-ABS-KEY%28%22skin+injuries%22%29+OR+TITLE-ABS-KEY%28%22skin+fibrosis%22%29+OR+TITLE-ABS-KEY%28%22skin+scars%22%29%29%29&relpos=397&citeCnt=3&searchTerm=) \| \| [Oral administration of marine collagen peptides prepared from chum salmon (*Oncorhynchus keta*) improves wound healing following cesarean section in rats](https://www-scopus.ez35.periodicos.capes.gov.br/record/display.uri?eid=2-s2.0-84929493003&origin=resultslist&sort=plf-f&src=s&nlo=&nlr=&nls=&mltAll=t&sid=8f682cda9d1e11ee420f15ef65811fea&sot=comb&sdt=cl&cluster=scoexactkeywords%2c%22Animal+Experiment%22%2ct%2bscolang%2c%22English%22%2ct&sl=1001&s=%28%28TITLE-ABS-KEY%28%22peptides%22%29+OR+TITLE-ABS-KEY%28%22antioxidant+peptides%22%29+OR+TITLE-ABS-KEY+%28%22antimicrobial+peptides%22%29+OR+TITLE-ABS-KEY%28%22angiogenic+peptides%22%29+OR+TITLE-ABS-KEY+%28%22animal+peptides%22%29+OR+TITLE-ABS-KEY%28%22natural+peptides%22%29+OR+TITLE-ABS-KEY%28%22bioactive+peptides%22%29+OR+TITLE-ABS-KEY%28%22biological+peptides%22%29+OR+TITLE-ABS-KEY%28%22isolated+peptides%22%29+OR+TITLE-ABS-KEY%28%22extracted+peptides%22%29%29%29+AND+%28%28TITLE-ABS-KEY%28%22wound+healing%22%29+OR+TITLE-ABS-KEY%28%22regeneration%22%29+OR+TITLE-ABS-KEY%28%22skin+repair%22%29+OR+TITLE-ABS-KEY%28%22cutaneous+repair%22%29+OR+TITLE-ABS-KEY%28%22skin+healing%22%29+OR+TITLE-ABS-KEY%28%22cutaneous+healing%22%29%29%29+AND+%28%28TITLE-ABS-KEY%28%22skin%22%29+OR+TITLE-ABS-KEY%28%22dermis%22%29+OR+TITLE-ABS-KEY+%28%22epidermis%22%29+OR+TITLE-ABS-KEY%28%22subcutaneous+tissue%22%29+OR+TITLE-ABS-KEY%28%22hypodermis%22%29+OR+TITLE-ABS-KEY%28%22granulation+tissue%22%29+OR+TITLE-ABS-KEY%28%22keratinocytes%22%29+OR+TITLE-ABS-KEY%28%22fibroblasts%22%29+OR+TITLE-ABS-KEY+%28%22integumentary+system%22%29+OR+TITLE-ABS-KEY%28%22skin+injuries%22%29+OR+TITLE-ABS-KEY%28%22skin+fibrosis%22%29+OR+TITLE-ABS-KEY%28%22skin+scars%22%29%29%29&relpos=245&citeCnt=16&searchTerm=) \| \| [Whey peptides improve wound healing following caesarean section in rats](https://www-scopus.ez35.periodicos.capes.gov.br/record/display.uri?eid=2-s2.0-78649982375&origin=resultslist&sort=plf-f&src=s&nlo=&nlr=&nls=&mltAll=t&sid=8f682cda9d1e11ee420f15ef65811fea&sot=comb&sdt=cl&cluster=scoexactkeywords%2c%22Animal+Experiment%22%2ct%2bscolang%2c%22English%22%2ct&sl=1001&s=%28%28TITLE-ABS-KEY%28%22peptides%22%29+OR+TITLE-ABS-KEY%28%22antioxidant+peptides%22%29+OR+TITLE-ABS-KEY+%28%22antimicrobial+peptides%22%29+OR+TITLE-ABS-KEY%28%22angiogenic+peptides%22%29+OR+TITLE-ABS-KEY+%28%22animal+peptides%22%29+OR+TITLE-ABS-KEY%28%22natural+peptides%22%29+OR+TITLE-ABS-KEY%28%22bioactive+peptides%22%29+OR+TITLE-ABS-KEY%28%22biological+peptides%22%29+OR+TITLE-ABS-KEY%28%22isolated+peptides%22%29+OR+TITLE-ABS-KEY%28%22extracted+peptides%22%29%29%29+AND+%28%28TITLE-ABS-KEY%28%22wound+healing%22%29+OR+TITLE-ABS-KEY%28%22regeneration%22%29+OR+TITLE-ABS-KEY%28%22skin+repair%22%29+OR+TITLE-ABS-KEY%28%22cutaneous+repair%22%29+OR+TITLE-ABS-KEY%28%22skin+healing%22%29+OR+TITLE-ABS-KEY%28%22cutaneous+healing%22%29%29%29+AND+%28%28TITLE-ABS-KEY%28%22skin%22%29+OR+TITLE-ABS-KEY%28%22dermis%22%29+OR+TITLE-ABS-KEY+%28%22epidermis%22%29+OR+TITLE-ABS-KEY%28%22subcutaneous+tissue%22%29+OR+TITLE-ABS-KEY%28%22hypodermis%22%29+OR+TITLE-ABS-KEY%28%22granulation+tissue%22%29+OR+TITLE-ABS-KEY%28%22keratinocytes%22%29+OR+TITLE-ABS-KEY%28%22fibroblasts%22%29+OR+TITLE-ABS-KEY+%28%22integumentary+system%22%29+OR+TITLE-ABS-KEY%28%22skin+injuries%22%29+OR+TITLE-ABS-KEY%28%22skin+fibrosis%22%29+OR+TITLE-ABS-KEY%28%22skin+scars%22%29%29%29&relpos=465&citeCnt=4&searchTerm=) \| | 5 |
| Wound healing not evaluated |  | \| [Acceleration of soft tissue repair by a thrombin-derived oligopeptide](https://www.ncbi.nlm.nih.gov/pubmed/1405599) \| \| --- \| \| [Annexin A12-26 treatment improves skin heterologous transplantation by modulating inflammation and angiogenesis processes](https://www2.scopus.com/record/display.uri?eid=2-s2.0-85053122625&origin=resultslist&sort=plf-f&src=s&nlo=&nlr=&nls=&mltAll=t&sid=4d2dea855575f6fbdb403e5ce008a978&sot=comb&sdt=cl&cluster=scoexactkeywords%2c%22Animal+Experiment%22%2ct%2bscolang%2c%22English%22%2ct&sl=1001&s=%28%28TITLE-ABS-KEY%28%22peptides%22%29+OR+TITLE-ABS-KEY%28%22antioxidant+peptides%22%29+OR+TITLE-ABS-KEY+%28%22antimicrobial+peptides%22%29+OR+TITLE-ABS-KEY%28%22angiogenic+peptides%22%29+OR+TITLE-ABS-KEY+%28%22animal+peptides%22%29+OR+TITLE-ABS-KEY%28%22natural+peptides%22%29+OR+TITLE-ABS-KEY%28%22bioactive+peptides%22%29+OR+TITLE-ABS-KEY%28%22biological+peptides%22%29+OR+TITLE-ABS-KEY%28%22isolated+peptides%22%29+OR+TITLE-ABS-KEY%28%22extracted+peptides%22%29%29%29+AND+%28%28TITLE-ABS-KEY%28%22wound+healing%22%29+OR+TITLE-ABS-KEY%28%22regeneration%22%29+OR+TITLE-ABS-KEY%28%22skin+repair%22%29+OR+TITLE-ABS-KEY%28%22cutaneous+repair%22%29+OR+TITLE-ABS-KEY%28%22skin+healing%22%29+OR+TITLE-ABS-KEY%28%22cutaneous+healing%22%29%29%29+AND+%28%28TITLE-ABS-KEY%28%22skin%22%29+OR+TITLE-ABS-KEY%28%22dermis%22%29+OR+TITLE-ABS-KEY+%28%22epidermis%22%29+OR+TITLE-ABS-KEY%28%22subcutaneous+tissue%22%29+OR+TITLE-ABS-KEY%28%22hypodermis%22%29+OR+TITLE-ABS-KEY%28%22granulation+tissue%22%29+OR+TITLE-ABS-KEY%28%22keratinocytes%22%29+OR+TITLE-ABS-KEY%28%22fibroblasts%22%29+OR+TITLE-ABS-KEY+%28%22integumentary+system%22%29+OR+TITLE-ABS-KEY%28%22skin+injuries%22%29+OR+TITLE-ABS-KEY%28%22skin+fibrosis%22%29+OR+TITLE-ABS-KEY%28%22skin+scars%22%29%29%29&relpos=50&citeCnt=1&searchTerm=) \| \| [Blood vessel occlusion in peri-burn tissue is secondary to erythrocyte aggregation and mitigated by a fibronectin-derived peptide that limits burn injury progression](https://www-scopus.ez35.periodicos.capes.gov.br/record/display.uri?eid=2-s2.0-84964655336&origin=resultslist&sort=plf-f&src=s&nlo=&nlr=&nls=&mltAll=t&sid=8f682cda9d1e11ee420f15ef65811fea&sot=comb&sdt=cl&cluster=scoexactkeywords%2c%22Animal+Experiment%22%2ct%2bscolang%2c%22English%22%2ct&sl=1001&s=%28%28TITLE-ABS-KEY%28%22peptides%22%29+OR+TITLE-ABS-KEY%28%22antioxidant+peptides%22%29+OR+TITLE-ABS-KEY+%28%22antimicrobial+peptides%22%29+OR+TITLE-ABS-KEY%28%22angiogenic+peptides%22%29+OR+TITLE-ABS-KEY+%28%22animal+peptides%22%29+OR+TITLE-ABS-KEY%28%22natural+peptides%22%29+OR+TITLE-ABS-KEY%28%22bioactive+peptides%22%29+OR+TITLE-ABS-KEY%28%22biological+peptides%22%29+OR+TITLE-ABS-KEY%28%22isolated+peptides%22%29+OR+TITLE-ABS-KEY%28%22extracted+peptides%22%29%29%29+AND+%28%28TITLE-ABS-KEY%28%22wound+healing%22%29+OR+TITLE-ABS-KEY%28%22regeneration%22%29+OR+TITLE-ABS-KEY%28%22skin+repair%22%29+OR+TITLE-ABS-KEY%28%22cutaneous+repair%22%29+OR+TITLE-ABS-KEY%28%22skin+healing%22%29+OR+TITLE-ABS-KEY%28%22cutaneous+healing%22%29%29%29+AND+%28%28TITLE-ABS-KEY%28%22skin%22%29+OR+TITLE-ABS-KEY%28%22dermis%22%29+OR+TITLE-ABS-KEY+%28%22epidermis%22%29+OR+TITLE-ABS-KEY%28%22subcutaneous+tissue%22%29+OR+TITLE-ABS-KEY%28%22hypodermis%22%29+OR+TITLE-ABS-KEY%28%22granulation+tissue%22%29+OR+TITLE-ABS-KEY%28%22keratinocytes%22%29+OR+TITLE-ABS-KEY%28%22fibroblasts%22%29+OR+TITLE-ABS-KEY+%28%22integumentary+system%22%29+OR+TITLE-ABS-KEY%28%22skin+injuries%22%29+OR+TITLE-ABS-KEY%28%22skin+fibrosis%22%29+OR+TITLE-ABS-KEY%28%22skin+scars%22%29%29%29&relpos=195&citeCnt=6&searchTerm=) \| \| [Effect of peptide bioregulator on healing of excision wounds in old animals](https://www-scopus.ez35.periodicos.capes.gov.br/record/display.uri?eid=2-s2.0-77954426324&origin=resultslist&sort=plf-f&src=s&nlo=&nlr=&nls=&mltAll=t&sid=8f682cda9d1e11ee420f15ef65811fea&sot=comb&sdt=cl&cluster=scoexactkeywords%2c%22Animal+Experiment%22%2ct%2bscolang%2c%22English%22%2ct&sl=1001&s=%28%28TITLE-ABS-KEY%28%22peptides%22%29+OR+TITLE-ABS-KEY%28%22antioxidant+peptides%22%29+OR+TITLE-ABS-KEY+%28%22antimicrobial+peptides%22%29+OR+TITLE-ABS-KEY%28%22angiogenic+peptides%22%29+OR+TITLE-ABS-KEY+%28%22animal+peptides%22%29+OR+TITLE-ABS-KEY%28%22natural+peptides%22%29+OR+TITLE-ABS-KEY%28%22bioactive+peptides%22%29+OR+TITLE-ABS-KEY%28%22biological+peptides%22%29+OR+TITLE-ABS-KEY%28%22isolated+peptides%22%29+OR+TITLE-ABS-KEY%28%22extracted+peptides%22%29%29%29+AND+%28%28TITLE-ABS-KEY%28%22wound+healing%22%29+OR+TITLE-ABS-KEY%28%22regeneration%22%29+OR+TITLE-ABS-KEY%28%22skin+repair%22%29+OR+TITLE-ABS-KEY%28%22cutaneous+repair%22%29+OR+TITLE-ABS-KEY%28%22skin+healing%22%29+OR+TITLE-ABS-KEY%28%22cutaneous+healing%22%29%29%29+AND+%28%28TITLE-ABS-KEY%28%22skin%22%29+OR+TITLE-ABS-KEY%28%22dermis%22%29+OR+TITLE-ABS-KEY+%28%22epidermis%22%29+OR+TITLE-ABS-KEY%28%22subcutaneous+tissue%22%29+OR+TITLE-ABS-KEY%28%22hypodermis%22%29+OR+TITLE-ABS-KEY%28%22granulation+tissue%22%29+OR+TITLE-ABS-KEY%28%22keratinocytes%22%29+OR+TITLE-ABS-KEY%28%22fibroblasts%22%29+OR+TITLE-ABS-KEY+%28%22integumentary+system%22%29+OR+TITLE-ABS-KEY%28%22skin+injuries%22%29+OR+TITLE-ABS-KEY%28%22skin+fibrosis%22%29+OR+TITLE-ABS-KEY%28%22skin+scars%22%29%29%29&relpos=506&citeCnt=0&searchTerm=) \| | 4 |
| **Total** |  |  | **108** |
